# Novel Maleimide Linkers Based on a Piperazine Motif for Strongly Increased Aqueous Solubility

**DOI:** 10.1021/acsomega.4c10825

**Published:** 2025-01-31

**Authors:** Martijn Dijkstra, Hemma Schueffl, Anja Federa, Caroline Kast, Alexander Unterlercher, Bernhard K. Keppler, Petra Heffeter, Christian R. Kowol

**Affiliations:** † University of Vienna, Faculty of Chemistry, Institute of Inorganic Chemistry, Waehringer Str. 42, 1090 Vienna, Austria; ‡ University of Vienna, Vienna Doctoral School in Chemistry (DoSChem), Waehringer Str. 42, 1090 Vienna, Austria; § Center for Cancer Research and Comprehensive Cancer Center, Medical University of Vienna, Borschkegasse 8a, 1090 Vienna, Austria; ∥ Research Cluster “Translational Cancer Therapy Research”, 1090 Vienna, Austria

## Abstract

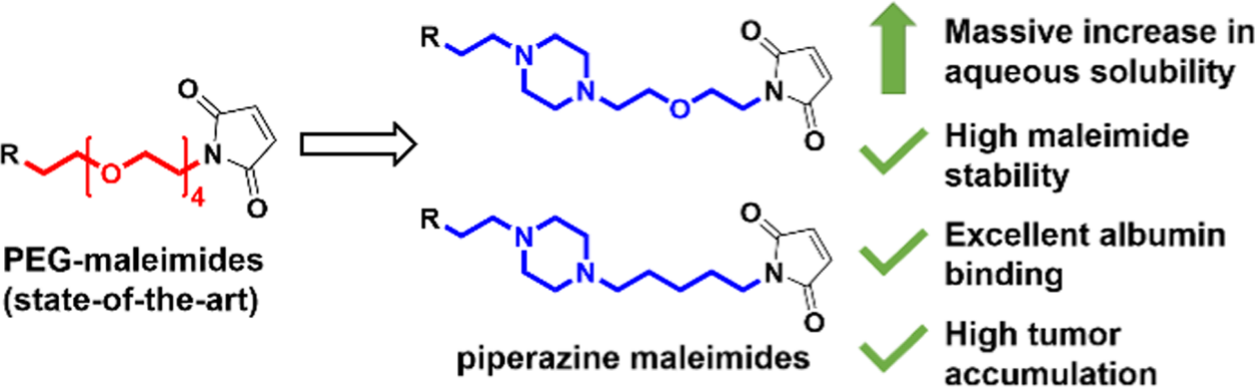

Maleimides remain very popular conjugation moieties in the fields of bio(in)organic chemistry and biotechnology. They are particularly interesting for endogenous albumin binding in the bloodstream to exploit the enhanced permeability and retention (EPR) effect and to increase tumor accumulation of anticancer drugs. However, during drug development, insufficient aqueous solubility is frequently a limiting factor. In the present study, four new maleimide linkers were synthesized containing a water-soluble piperazine scaffold. Respective maleimide-platinum(IV)-acetato complexes demonstrated similar hydrolytic stability, albumin-binding kinetics, *in vivo* serum pharmacokinetics and tissue distribution compared to a reference platinum(IV)-PEG4-maleimide complex. To test the aqueous solubility, platinum(IV)-maleimide complexes containing the highly lipophilic drug ibuprofen were synthesized. Indeed, the compounds containing the new piperazine linkers displayed increased solubility (up to 370 mM) in different aqueous media, whereas the PEG4-maleimide reference was only marginally soluble. Finally, the synthetic toolbox of the new piperazine maleimides was also expanded to pure organic derivatives by conjugation to valine-citrulline-*para*-aminobenzyl–OH derivatives via peptide and thiourea bonds.

## Introduction

The thiol-Michael addition (i.e., thiol–ene click reaction) is a versatile and powerful conjugation tool widely applied in materials chemistry^1,2^ and biochemistry.^3−5^ Probably the most famous thiol-Michael addition is the reaction with maleimides, frequently used in connecting (pro)drugs to cysteine residues of e.g., proteins^6,7^ or antibodies.^8,9^ Cysteine conjugation via maleimides is usually employed for the synthesis of antibody-drug conjugates (ADCs), which spearhead the field of novel targeted anticancer drugs.^10,11^ The clinical potential of ADCs for cancer therapy is illustrated by prominent FDA approvals such as trastuzumab deruxtecan, enfortumab vedotin, or brentuximab vedotin.^12^ Additionally, maleimides are extensively used as passive tumor-targeting moieties for endogenous conjugation of drugs to the cysteine-34 residue of human serum albumin (HSA) in the bloodstream after intravenous application. This leads to increased accumulation of drug conjugates in the tumor microenvironment via the enhanced permeability and retention (EPR) effect.^13^ A famous example is the tumor-targeted doxorubicin derivative aldoxorubicin, which advanced into phase III clinical trials for treating patients with soft tissue sarcomas.^14^

Sufficient aqueous solubility is a crucial factor of drug design that has long proven to be a formidable obstacle in the successful clinical translation of many promising pipeline drugs and lead candidates.^15^ In particular, poor solubility is a major limitation in small molecule drug development associated with low bioavailability and even the risk of cell or tissue precipitation-associated toxicity.^16^ In the past decades, numerous strategies were developed to improve the solubility of drugs, which can be mainly divided in physical modifications, such as micronization or solid dispersion,^17^ host–guest complexation techniques^18,19^ and chemical modifications.^20,21^ Nitrogen heterocycles in particular play a vital role in medicinal chemistry, not only as the structural backbone of about 75% of all approved drugs,^22,23^ but also as a unit to improve aqueous solubility. In particular, piperazines rank as the third most commonly used saturated nitrogen heterocycles in drug discovery,^23^ and the piperazine motif is encountered in many approved drugs,^24^ such as the tyrosine kinase inhibitors imatinib^25^ and nintedanib.^26^

In our group, we demonstrated in recent years that albumin-targeted platinum(IV)-maleimide complexes have distinctly improved anticancer activity compared to oxaliplatin, due to a significantly increased plasma half-life and enhanced intratumoral accumulation via the EPR effect.^27,28^ The antitumor activity could be even further improved by the development of tumor-targeted dual-action platinum(IV) prodrugs bearing additional bioactive ligands for synergistic effects.^29,30^ However, highly functionalized maleimide-bearing platinum(IV) complexes often lack sufficient aqueous solubility for intravenous administration in *in vivo* studies. This is especially evident for prodrugs based on carboplatin, where high dosages are applicable due to the low toxicity. Currently, poly(ethylene glycol) (PEG) maleimide linkers are state-of-the-art in increasing the solubility, but often, the desired aqueous solubility can still not be reached. Thus, encouraged by our previous experiences in the development of maleimide-based drugs, and inspired by the pharmaceutical value of the piperazine scaffold, we envisioned that the development of novel, water-soluble maleimides and drug derivatives thereof could significantly expand the medicinal chemist’s toolbox. In the present study, we report on the synthesis of a series of maleimide linkers based on a piperazine scaffold,^31^ and attached them to platinum(IV) prodrugs bearing the nonsteroidal anti-inflammatory drug (NSAID) ibuprofen.^32,33^ The *in vitro* antitumor activity of cisplatin(IV) and oxaliplatin(IV)-NSAID complexes has been well-studied in the past due to the potential synergism between cyclooxygenase-2 inhibition and platinum drugs. Furthermore, ibuprofen is highly lipophilic and thus an ideal candidate to investigate the impact of the piperazine linker moiety on drug solubility. In parallel, the new maleimides were attached to platinum(IV)-acetato complexes for the investigation of the hydrolytic stability, albumin binding and serum pharmacokinetics/organ distribution *in vivo* in comparison to the common PEG4-containing maleimide derivative. Finally, we demonstrate as proof-of-principle that the new maleimide linkers can be modified with different functional groups, allowing coupling to other carboxylic acids or amino groups via peptide or thiourea moieties. Therefore, the cathepsin B-cleavable linker valine-citrulline-*para*-aminobenzyl–OH (H_2_N-ValCitPAB–OH) was used, which is extensively employed as an intermediate in the synthesis of antitumor ADCs.^34^

## Results and Discussion

### Synthesis of Piperazine Maleimides and Their Platinum(IV) Complexes

**Scheme 1:**
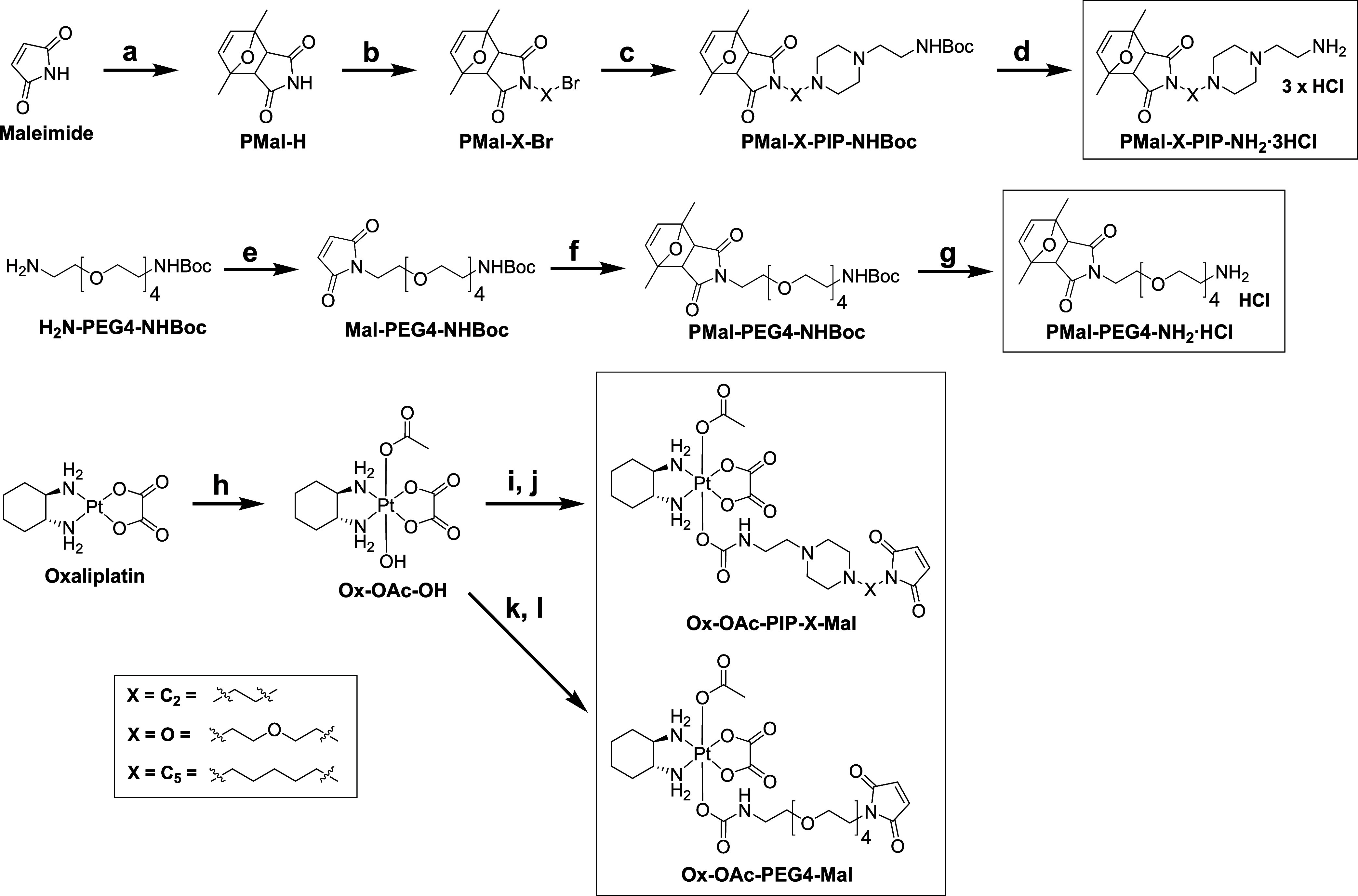
Synthetic Routes for Piperazine Maleimides and Their Platinum(IV)–Acetato Complexes^a^ Reaction conditions: (a) 2,5-dimethylfurane, acetonitrile (ACN), 60 °C; (b) dibromoalkane, dimethylformamide (DMF), 50 °C (yield: X = C_2_: 67%; O: 64%; C_5_: 62%); (c) 1-(2-Boc-aminoethyl)-piperazine, triethylamine (TEA), DMF (yield: X = C_2_: 52%; O: 65%; C_5_: 73%); (d) 1.25M HCl, EtOH (yield: X = C_2_: 99%; O: 95%; C_5_: 95%); (e) methyl 2,5-dioxo-2,5-dihydro-1*H*-pyrrole-1-carboxylate, saturated NaHCO_3_ solution, 0 °C–RT (directly used in the next step); (f) 2,5-dimethylfurane, acetonitrile (ACN), 60 °C (yield: 79%); (g) 1.25M HCl, EtOH (yield: 91%); (h) H_2_O_2_, (50 wt %), acetic acid (AcOH); (i) PMal-X-PIP-NH_2_·3HCl (prior neutralized), DMF (Yield: X = C_2_: 72%; O: 72%; C_5_: 73%); (j) dimethyl sulfoxide (DMSO), 90 °C (yield: X = C_2_: 60%; O: 82%; C_5_: 61%); (k) PMal-PEG4-NH_2_·HCl (prior neutralized), DMF (yield: 68%); (l) DMSO, 90 °C (yield: 68%).

In the first step, a convergent and straightforward synthetic pathway toward piperazine maleimides was designed that bear a terminal primary amine, which is an indispensable functional group for medicinal chemists.^35^ To this end, the sensitive maleimide moiety was protected via the formation of a Diels–Alder cycloadduct by reacting it with an excess of 2,5-dimethylfurane at 60 °C (PMal-H, Scheme 1). Subsequently, different bromoalkyl chains were introduced via a substitution reaction with PMal-H using various dibromoalkanes (Br-X-Br), namely BrCH_2_CH_2_Br (“C_2_”), Br(CH_2_)_5_Br (“C_5_”), or BrCH_2_CH_2_OCH_2_CH_2_Br (“O”) to yield PMal-C_2_-Br, PMal-C_5_-Br and PMal-O-Br, respectively. These intermediates were then used for the synthesis of amine-functionalized piperazine maleimides (PMal-X-PIP-NHBoc) via a second substitution reaction with commercially available 1-(2-Boc-aminoethyl)-piperazine, which after Boc-deprotection yielded the final compounds PMal-X-PIP-NH_2_·3HCl. In parallel, a reference maleimide derivative based on a PEG linker was synthesized, also bearing a terminal amine. To that end, (14-amino-3,6,9,12-tetraoxatetradecyl)carbamate (H_2_N-PEG4-NHBoc) was reacted with methyl 2,5-dioxo-2,5-dihydro-1*H*-pyrrole-1-carboxylate in saturated sodium bicarbonate solution to generate Mal-PEG4-NHBoc (Scheme 1). The maleimide moiety was again protected via a reaction with 2,5-dimethylfurane at 60 °C (PMal-PEG4-NHBoc), which after Boc-deprotection afforded PMal-PEG4-NH_2_·HCl.

With the novel PMal-X-PIP-NH_2_·3HCl and PMal-PEG4-NH_2_·HCl compounds in hand, a panel of platinum(IV) complexes with an axial acetato ligand was developed (Scheme 1). To that end, oxaliplatin was synthesized as described in literature^36^ and oxidized^37^ asymmetrically to Ox-OAc-OH. Subsequently, Ox-OAc-OH was activated with the peptide-coupling agent *N*,*N*′-disuccinimidyl carbonate (DSC), after which each of the prior neutralized maleimide ligands was attached via a carbamate linkage. In the last step, the maleimide moiety was deprotected by release of 2,5-dimethylfurane via a retro-Diels–Alder reaction at 90 °C to obtain the final complexes **Ox-OAc-PIP-C**_**2**_-**Mal**, **Ox-OAc-PIP-O-Mal**, **Ox-OAc-PIP-C**_**5**_**-Mal**, and **Ox-OAc-PEG4-Mal**.

### Maleimide Hydrolytic Stability of Platinum(IV) Complexes

**Figure 1:**
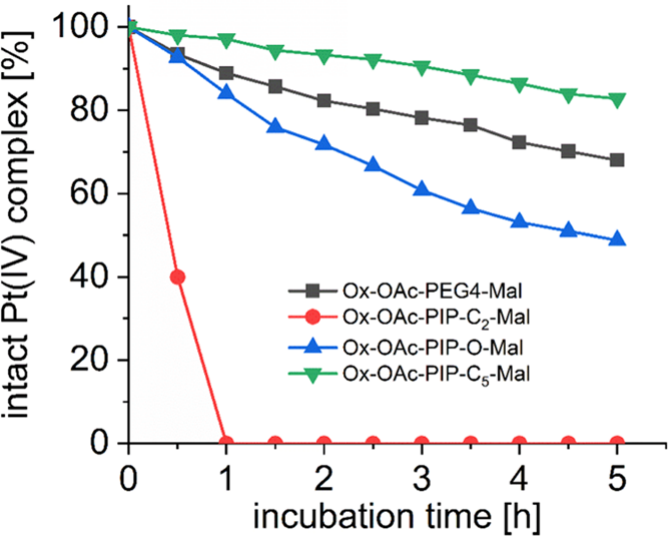
Hydrolytic stability of 1 mM **Ox-OAc-PEG4-Mal**, **Ox-OAc-PIP-C**_**2**_**-Mal**, **Ox-OAc-PIP-O-Mal**, and **Ox-OAc-PIP-C**_**5**_**-Mal** in 150 mM phosphate buffer (pH = 7.4) at 20 °C over 5 h, measured with UHPLC.

It is known from literature that most maleimides are prone to hydrolysis at physiological pH.^38^ Consequently, the hydrolytic stabilities of the three oxaliplatin(IV)-acetato complexes bearing the piperazine maleimides were studied in comparison to the reference complex **Ox-OAc-PEG4-Mal**. The hydrolysis of the platinum(IV)-acetato complexes was investigated in phosphate buffer at pH 7.4 and 20 °C over 5 h and the amount of remaining intact parental complex was monitored with ultrahigh-performance liquid chromatography (UHPLC) (Figure 1). **Ox-OAc-PIP-C**_**2**_-**Mal** hydrolyzed rapidly, with only traces of intact platinum(IV)-maleimide remaining after 1 h incubation time. In comparison, **Ox-OAc-PIP-O-Mal** showed ∼50% intact maleimide after 5 h incubation and **Ox-OAc-PIP-C**_**5**_**-Mal** even 80%, demonstrating that the nature of the linker between piperazine and maleimide has a strong influence on the rate of hydrolysis. These dramatic differences are in line with some model maleimide compounds reported in literature, also containing piperazine moieties.^38^ The strong inductive effects of the protonated quaternary nitrogen of the piperazine can explain the extremely fast hydrolysis of **Ox-OAc-PIP-C**_**2**_**-Mal**. This effect is distinctly reduced when the linker length between maleimide and piperazine is increased via the introduction of a “–(CH_2_)_2_O(CH_2_)_2_–” or “–(CH_2_)_5_–” fragment. The hydrolytic stability of the reference complex **Ox-OAc-PEG4-Mal** with ∼70% intact complex after 5 h was in between the rates of **Ox-OAc-PIP-O-Mal** and **Ox-OAc-PIP-C**_**5**_**-Mal**, showing that the piperazine motif is still affecting the maleimide hydrolysis.

### Albumin Binding and *In Vivo* Pharmacokinetics of Platinum(IV) Complexes

**Figure 2:**
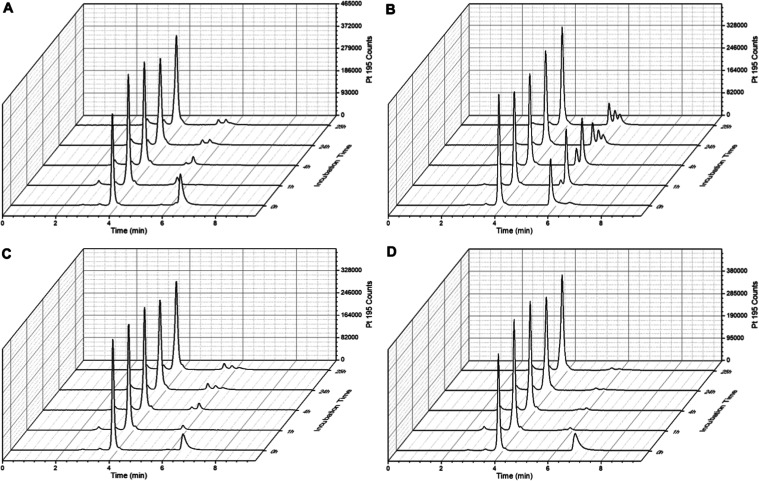
^195^Pt-traces of incubation of 100 μM (A) **Ox-OAc-PEG4-Mal**, (B) **Ox-OAc-PIP-C**_**2**_**-Mal**, (C) **Ox-OAc-PIP-O-Mal**, (D) **Ox-OAc-PIP-C**_**5**_**-Mal** in FCS (containing 150 mM phosphate buffer, pH = 7.4) at 37 °C over 25 h, measured with SEC-ICP-MS.

Subsequently, it was of interest how the observed divergent maleimide hydrolysis rates impact the albumin binding kinetics. Therefore, the different platinum(IV) complexes were incubated in phosphate-buffered fetal calf serum (FCS) at 37 °C over 25 h and ^195^Pt traces were measured via size-exclusion chromatographyinductively coupled plasma mass spectrometry (SEC-ICP-MS). Serum proteins (including albumin) have a retention time of 2–4 min, with the albumin fraction eluting at 4.0 min (Figure S1) as part of the high-molecular weight fraction (HMWF) and unbound platinum(IV) complexes elute in the low-molecular weight fraction (LMWF) with retention times usually >5 min. Already at time point 0, ∼60% of **Ox-OAc-PEG4-Mal** (*t*_R_ = 6.4 min) was bound to albumin at 4.0 min in the HMWF, which increased to ∼90% after 1 h of incubation (Figure 2A). The remaining 10% of the parental complex (at *t*_R_ > 5 min) most likely either contained a hydrolyzed maleimide moiety, unable to bind albumin, or were conjugated to low-molecular weight sulfur-containing compounds. The formed platinum(IV)-albumin conjugate remained stable for more than 25 h. Also **Ox-OAc-PIP-C**_**2**_-**Mal** showed ∼60% albumin binding at time point 0, which, however, did not further increase over time (Figure 2B). In the LMWF, only a small peak of still intact **Ox-OAc-PIP-C**_**2**_-**Mal** (*t*_R_ 6.5 min) could be observed, while a major signal (∼35%) of most likely already hydrolyzed maleimide compound appeared at *t*_R_ 5.8 min. This is in very good agreement with the very fast hydrolysis of the maleimide moiety shown above (see Figure 1). The albumin-binding kinetics of **Ox-OAc-PIP-O-Mal** and **Ox-OAc-PIP-C**_**5**_**-Mal** were faster (75 and 69% bound at time point 0, respectively) compared to **Ox-OAc-PEG4-Mal**, and a nearly complete conjugation of ∼95% already after 1 h incubation could be observed (Figure 2C,D).

**Figure 3:**
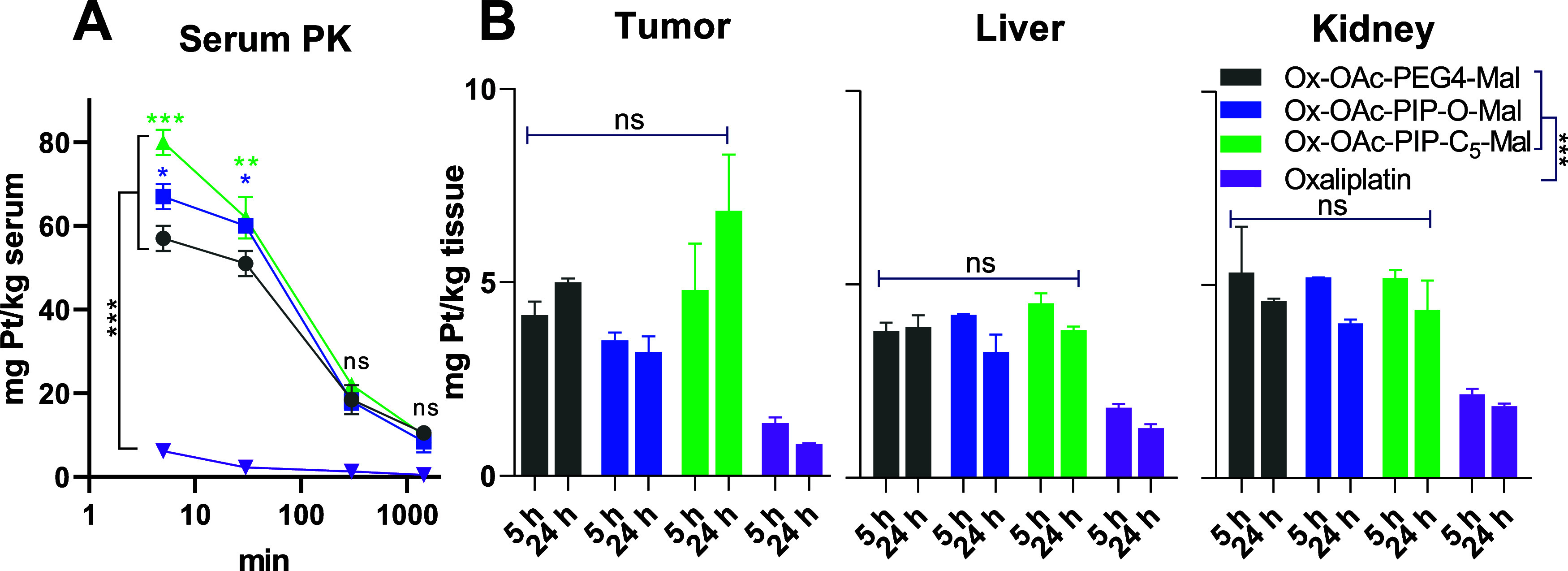
Pharmacological behavior of the tested drugs in blood and organs. Animals (*n* = 2 per treatment group and time point) were treated once i.v. with concentrations equimolar to 9 mg/kg oxaliplatin. Blood samples were taken at intervals between 5 min and 24 h. Serum was generated by centrifugation after clotting at room temperature. Organ and tumor samples were collected either after 5 or 24 h. Platinum levels of all samples were measured via ICP-MS: (A) serum samples and (B) tumor, liver and kidney samples. Statistical significance for: (A) was tested by two-way ANOVA comparing the platinum(IV)-maleimide drugs to oxaliplatin and Dunnett’s multiple comparisons test between **Ox-OAc-PEG4-Mal** and the two PIP-functionalized drugs. (B) was tested by two-way ANOVA comparing the platinum(IV)-maleimide drugs to oxaliplatin as well the platinum(IV)-maleimide drugs to each other. (***p < 0.001, **p < 0.01 and *p < 0.05, ns = not significant). The oxaliplatin data were used from Schueffl et al.^39^

As a next step, we were interested whether the insertion of the piperazine moiety influences the pharmacological behavior of the drugs *in vivo*. To get more insights into the pharmacokinetics (PK) and biodistribution profiles, **Ox-OAc-PIP-O-Mal** and **Ox-OAc-PIP-C**_**5**_**-Mal** were investigated in comparison to **Ox-OAc-PEG4-Mal** in Balb/c mice bearing subcutaneous CT26 tumors. We excluded **Ox-OAc-PIP-C**_**2**_**-Mal** from these studies, because of the insufficient hydrolytic stability. Indeed, the maleimide-functionalization resulted in a distinctly prolonged plasma half-life time and enhanced drug accumulation in the malignant tissue compared to free oxaliplatin, well in accordance with literature (Figure 3).^28,39^ With regard to the plasma half-life, the two piperazine-containing complexes exhibited similar patterns compared to **Ox-OAc-PEG4-Mal** (Figure 3A). Only at the first two time points (5 and 30 min), **Ox-OAc-PIP-O-Mal** and **Ox-OAc-PIP-C**_**5**_**-Mal** treatment resulted in significantly higher platinum levels in the serum, which is in good agreement with their faster albumin binding (Figure 2). Also with regard to the biodistribution, the piperazine-containing complexes revealed a similar behavior compared to **Ox-OAc-PEG4-Mal**(Figures 3B and S2). Only in case of the tumor tissue, **Ox-OAc-PIP-C**_**5**_**-Mal** showed a trend toward enhanced accumulation. This is well in agreement with the elevated plasma platinum concentrations of **Ox-OAc-PIP-C**_**5**_**-Mal** over **Ox-OAc-PIP-O-Mal** and **Ox-OAc-PEG4-Mal**, which most probably originates from the higher hydrolytic stability of the maleimide unit observed for **Ox-OAc-PIP-C**_**5**_**-Mal** (Figure 1) enabling more efficient albumin binding. Of note, the data on **Ox-OAc-PEG4-Mal** were in good agreement with previously published data of PEG4-maleimide-containing oxaliplatin(IV) complexes.^28,39^

### Massive Aqueous Solubility Enhancement of Platinum(IV)-Ibuprofen Complexes Bearing Piperazine Maleimides

**Scheme 2:**
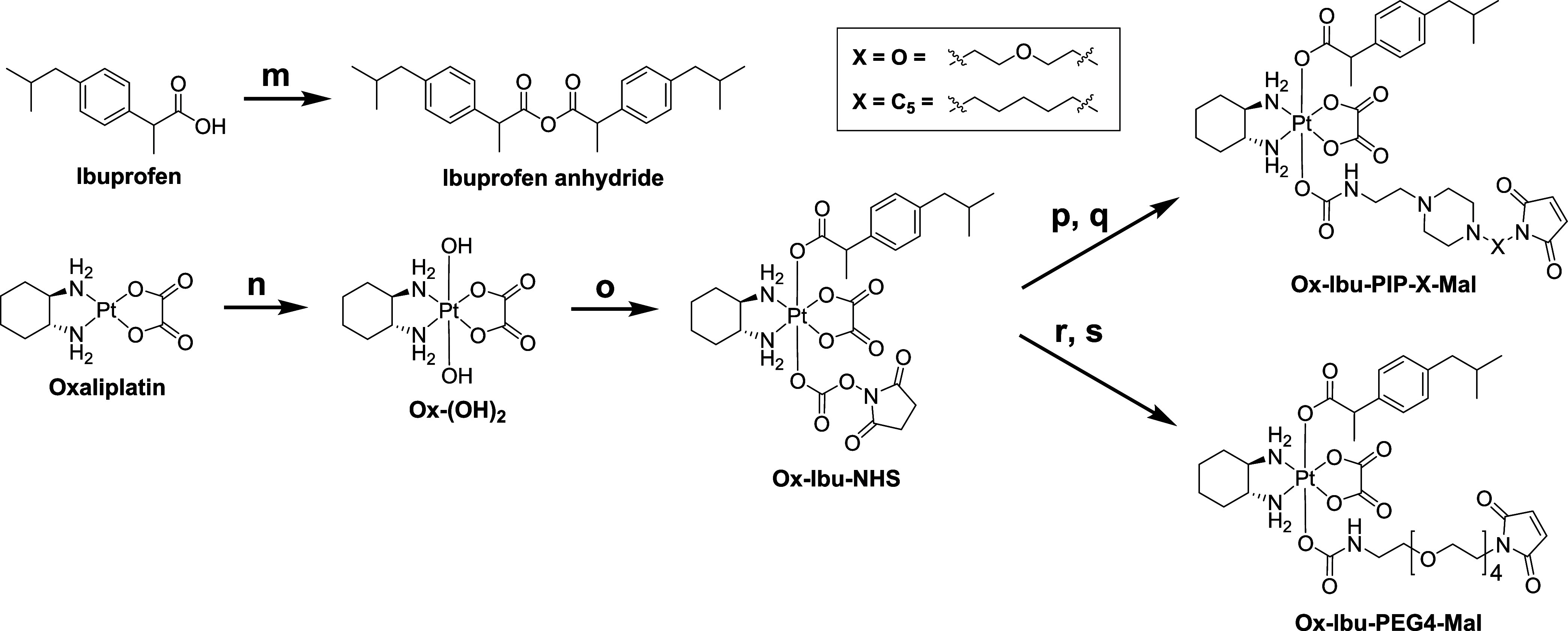
Synthetic Routes for the Platinum(IV)–Ibuprofen Complexes^a^ Reaction conditions: (m) *N*,*N*′-dicyclohexylcarbodiimide (DCC), dichloromethane (DCM) (yield: quant.); (n) H_2_O_2_ (50 wt %), MQ-H_2_O; (o) ibuprofen anhydride, DSC, DMF (yield: 73%); (p) PMal-X-PIP-NH_2_·3HCl (prior neutralized), DMF (yield: X = O: 71%; C_5_: 88%); (q) DMSO, 90 °C (yield: X = O: 90%; C_5_: 83%); (r) PMal-PEG4-NH_2_·HCl (prior neutralized), DMF (yield: 62%); (s) DMSO, 90 °C (yield: 70%).

To test our hypothesis that the piperazine scaffold can indeed strongly improve the aqueous solubility of the maleimides and drug derivatives thereof, we synthesized a series of ibuprofen platinum(IV) complexes. The NSAID inhibitor ibuprofen is highly lipophilic and, consequently, ideal for investigating the impact on aqueous solubility of our novel piperazine-containing maleimides compared to a reference PEG4 derivative (Scheme 2). Of note, some ibuprofen platinum(IV) compounds are already known in literature.^40^ Again, Mal-C_2_-PIP was excluded because of its insufficient hydrolytic stability. In more detail, ibuprofen was converted to ibuprofen anhydride via an adapted literature procedure,^41^ added dropwise to Ox-(OH)_2,_ after which an excess of DSC was added to afford the precursor complex Ox-Ibu-NHS. **Ox-Ibu-PIP-O-Mal**, **Ox-Ibu-PIP-C**_**5**_**-Mal**, and **Ox-Ibu-PEG4-Mal** were subsequently synthesized as the final platinum(IV)-ibuprofen complexes via a similar DSC-mediated pathway as described for the **Ox-OAc-PIP-X-Mal** complexes.

The solubilities of the **Ox-Ibu-PIP-X-Mal** complexes in comparison to **Ox-Ibu-PEG4-Mal** were studied in different biologically relevant media (Table 1). Initially, the solubility was investigated in 5% glucose, 0.9% NaCl and citrate buffer (pH 5), which are clinically used as standard solutions for intravenous injection of many (anticancer) drugs. In both media, **Ox-Ibu-PEG4-Mal** was, even with intensive sonication, only soluble to concentrations up to 0.2 mM. In contrast, both **Ox-Ibu-PIP-O-Mal** and **Ox-Ibu-PIP-C**_**5**_**-Mal** revealed extremely enhanced solubility, with **Ox-Ibu-PIP-O-Mal** showing the highest solubility in 0.9% NaCl up to 270 mM, which corresponds to an increase by a factor of ∼1700 compared to **Ox-Ibu-PEG4-Mal**. **Ox-Ibu-PIP-C**_**5**_**-Mal** was less soluble by a factor ∼10, but was still 30- to 130-fold better soluble compared to **Ox-Ibu-PEG4-Mal**. The disubstituted piperazine motif contains two tertiary amines with p*K*_a_ values of approximately 9 and 5.^42^ Thus, the impact of the pH value on the solubilities of the **Ox-Ibu-PIP-X-Mal** complexes was investigated. Commercially available 0.9% NaCl has a pH of roughly 5, but when acidified to pH 3, the solubilities of both **Ox-Ibu-PIP-X-Mal** complexes increased by a factor of 1.5. Conversely, when 0.9% NaCl was adjusted to pH 7, the solubility values dropped by a factor of ∼2.5–6.0 compared to pH 5, in line with the lower extent of piperazine protonation. Expectedly, using phosphate buffer or phosphate-buffered saline (PBS) at pH 7.4 further decreased the solubility. Nevertheless, **Ox-Ibu-PIP-O-Mal** was still 160- to 220-fold better soluble than **Ox-Ibu-PEG4-Mal**. For the piperazine-containing complexes, the solubility varied by a factor of 10 depending on the exact pH value, whereas the solubility of **Ox-Ibu-PEG4-Mal**, lacking protonatable functional groups, remained relatively unchanged (factor ∼2).

**Table 1:** Solubility of **Ox-Ibu-PIP-O-Mal** and **Ox-Ibu-PIP-C**_**5**_**-Mal** in Comparison to **Ox-Ibu-PEG4-Mal** in Different Biologically Relevant Media

		**Ox-Ibu-PEG4-Mal**	**Ox-Ibu-PIP-O-Mal**		**Ox-Ibu-PIP-C**_**5**_**-Mal**	
entry	medium	conc. (mM)	conc. (mM)	factor^a^	conc. (mM)	factor^a^
1	5% glucose	0.21	230	1100	20	100
2	0.9% NaCl	0.16	270	1700	20	130
3	citrate buffer (pH 5)	0.12	110	920	10	90
4	0.9% NaCl (pH 3)	0.15	370	2400	30	210
5	0.9% NaCl (pH 7)	0.13	70	490	5	40
6	PB (pH 7.4)	0.13	30	220	2	10
7	PBS (pH 7.4)	0.22	40	160	3	10

a Factors were calculated via dividing the measured concentrations of the respective **Ox-Ibu-PIP-X-Mal** complex and that of **Ox-Ibu-PEG4-Mal** in the same solvent. All solubilities were tested at least in duplicate.

When conducting an *in vivo* antitumor activity experiment of oxaliplatin(IV) prodrugs, often an equimolar amount to the maximum tolerated dose of oxaliplatin (normally 9 mg/kg = 4.5 mM) is applied intravenously. This would be impossible for **Ox-Ibu-PEG4-Mal** with a maximum solubility of ∼0.2 mM. Even when using solubilizers like 1,2-propandiol (propylene glycol), PEG400 or (hydroxypropyl)methyl cellulose (HPMC) polymer, this concentration could be by far not reached. In contrast, both **Ox-Ibu-PIP-C**_**5**_**-Mal** and **Ox-Ibu-PIP-O-Mal** complexes easily achieved the required solubilities (20–270 mM) without additives. Thus, these building blocks are highly interesting for significantly improving the solubility of maleimide-bearing drugs.

### Synthesis and Aqueous Solubility Enhancement of Valine-citrulline-*p*-aminobenzoyl Derivatives: Expansion to Organic Drugs

**Scheme 3:**
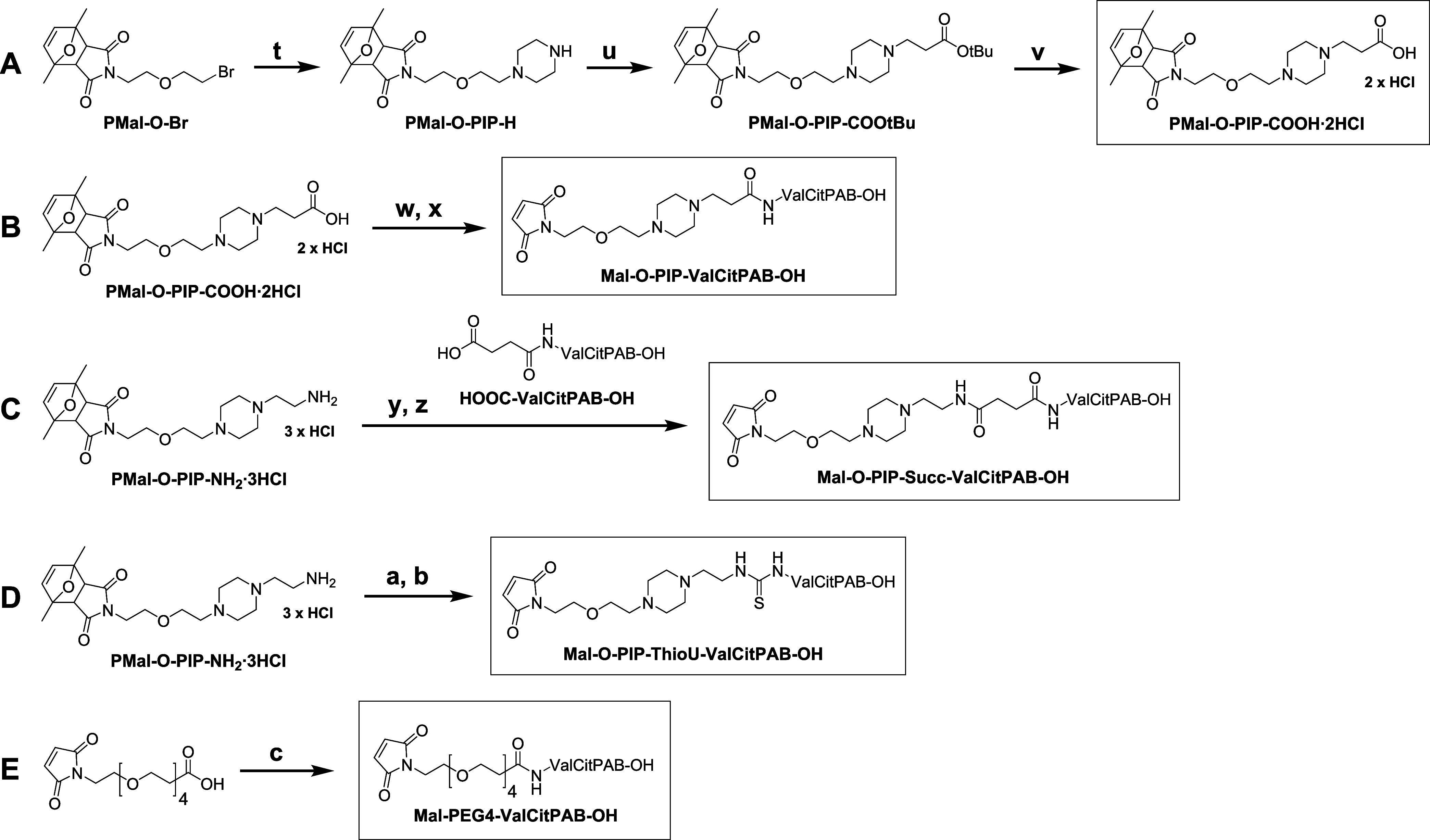
Synthetic Routes for PMal-O-PIP-COOH·2HCl and All ValCitPAB–OH Derivatives^a^ Reaction conditions: (t) Piperazine, K_2_CO_3_, DMF (yield: 60%); (u) *tert*-butyl bromopropionate, TEA, chloroform (CHCl_3_), 0 °C–RT (yield: 52%); (v) 4M HCl, dioxane (yield: 96%); (w) H_2_N-ValCitPAB–OH, EDC-HCl, HOBt, TEA, DMF (yield: 51%); (x) DMSO, 90 °C (yield: 65%); (y) HOOC-ValCitPAB–OH, TBTU, TEA, DMSO (yield: 70%); (z) DMSO, 90 °C (yield: 64%); (a) H_2_N-ValCitPAB–OH, TCDI, TEA, DMSO (yield: 64%); (b) DMSO, 90 °C (yield: 51%); (c) H_2_N-ValCitPAB–OH, HATU, DIPEA, DMSO.

Encouraged by the impressive aqueous solubility enhancement and the promising pharmacological properties, we wanted to further expand the versatility of our novel synthetic toolbox from metal complexes to organic molecules (e.g., for conjugation to peptides, proteins or antibodies). As a proof of concept, the valine-citrulline-*p*-aminobenzoyl fragment (*S*)*-*2-((*S*)-2-amino-3-methylbutanamido)-*N*-(4-(hydroxymethyl)phenyl)-5-ureidopentanamide (H_2_N-ValCitPAB–OH) was employed, which is a cleavable linker unit used in many (pro)drug delivery strategies.^34^ The focus was set on peptide and thiourea connections, which are privileged structures in medicinal chemistry.^43,44^ To this end, first a new piperazine maleimide derivative with a terminal carboxylic acid functionality (PMal-O-PIP-COOH) was synthesized (Scheme 3A). Monosubstitution of PMal–O-Br with an excess piperazine and consecutive reaction with *tert*-butyl bromopropionate yielded PMal-O-PIP-COOtBu, which was deprotected to afford PMal-O-PIP-COOH·2HCl (Scheme 3). PMal-O-PIP-COOH·2HCl was neutralized in excess TEA, mixed with the peptide-coupling agent 1-ethyl-3-(3-(dimethylamino)propyl)carbodiimide hydrochloride (EDC·HCl), hydroxybenzotriazole (HOBt) and H_2_N-ValCitPAB–OH to obtain PMal-O-PIP-ValCitPAB–OH. Finally, the maleimide moiety was deprotected at 90 °C to obtain **Mal-O-PIP-ValCitPAB–OH** (Scheme 3B).

To carry out the complementary reaction of PMal-O-PIP-NH_2_ with a carboxylic acid functionality, first H_2_N-ValCitPAB–OH was acylated with succinic anhydride to generate HOOC-ValCitPAB–OH. Subsequent activation with the peptide-coupling agent 2-(1*H*-benzotriazole-1-yl)-1,1,3,3-tetramethylammonium tetrafluoroborate (TBTU) and addition of prior neutralized PMal-O-PIP-NH_2_·3HCl afforded PMal-O-PIP-Succ-ValCitPAB–OH. Final maleimide deprotection at 90 °C generated **Mal-O-PIP-Succ-ValCitPAB–OH** (Scheme 3C).

As a third method, neutralized PMal-O-PIP-NH_2_·3HCl was directly connected to H_2_N-ValCitPAB–OH via a thiourea moiety in a one-pot synthesis using 1,1′-thiocarbonyldiimidazole (TCDI) to afford PMal-O-PIP-ThioU-ValCitPAB–OH. Deprotection of the maleimide yielded **Mal-O-PIP-ThioU-ValCitPAB–OH** (Scheme 3D).

Finally, the aqueous solubility enhancement of the three **Mal-X-PIP-ValCit-PAB–OH** linkers was investigated in 5% glucose and 0.9% NaCl solutions in comparison to a reference ValCit-PAB–OH linker containing a PEG4-maleimide (**Mal-PEG4-ValCitPAB–OH**; Scheme 3E; prepared as previously described in literature^45^). Although the reference ValCit linker **Mal-PEG4-ValCitPAB–OH** displayed already high solubility in the tested media itself (24 mM in 5% glucose and 35 mM in 0.9% NaCl) and, thus, is not an ideal reference compound, the three **Mal-X-PIP-ValCit-PAB–OH** derivatives were again distinctly better soluble with factors of ∼30 for 5% glucose (up to 600 mM) and ∼40 for 0.9% NaCl (up to 1300 mM; Table S1). It can be expected that upon derivatization of maleimide-containing ValCit linkers with e.g., lipophilic bioactive moieties, the introduction of the piperazine scaffold can again strongly boost the aqueous solubility compared to respective PEG4-derivatives, necessary to achieve sufficient solubility for biological applications.

## Conclusions

Maleimides are crucial in drug development due to their ability to react specifically with thiol groups, allowing precise conjugation to proteins (e.g., ADCs) and enhanced tumor accumulation via binding to endogenous serum albumin. However, especially for the development of maleimide-bearing small molecules, which are applied intravenously, insufficient aqueous solubility is a major concern. Thus, in this study, four new maleimide linkers (PMal-C_2_-PIP-NH_2_, PMal-O-PIP-NH_2_, PMal-C_5_-PIP-NH_2_, and PMal-O-PIP-COOH) were synthesized based on a water-soluble piperazine motif as well as three platinum(IV)-acetato complexes (**Ox-OAc-PIP-C**_**2**_-**Mal**, **Ox-OAc-PIP-O**-**Mal**, and **Ox-OAc-PIP-C**_**5**_-**Mal**). **Ox-OAc-PIP-C**_**2**_-**Mal** hydrolyzed extremely fast in PB at pH 7.4, which was also reflected by the inefficient albumin binding kinetics. In contrast, the hydrolysis and albumin-binding properties of **Ox-OAc-PIP-O-Mal** and **Ox-OAc-PIP-C**_**5**_**-Mal** were well comparable or even better than the reference complex **Ox-OAc-PEG4**-**Mal**. Also *in vivo* serum pharmacokinetics and tissue distribution studies of **Ox-OAc-PIP-O-Mal** and **Ox-OAc-PIP-C**_**5**_**-Mal** revealed the desired very high platinum serum levels and strong tumor accumulation. For solubility investigations, the two platinum(IV)-ibuprofen complexes (**Ox-Ibu-PIP-O**-**Mal** and **Ox-Ibu-PIP-C**_**5**_**-Mal**) were additionally synthesized. These compounds showed extensively increased aqueous solubility in different aqueous solutions (up to 370 mM), whereas the reference complex **Ox-Ibu-PEG4**-**Mal** displayed only marginal solubility in all tested media, far below the required dose for *in vivo* studies.

Finally, we expanded the synthetic usability of the piperazine maleimides also to pure organic molecules. Therefore, PMal-O-PIP-COOH and PMal-O-PIP-NH_2_ were connected to the well-known cathepsin B-cleavable linker ValCitPAB–OH via peptide and thiourea connections using a variety of coupling agents. Also, these new derivatives showed the desired increase in solubility compared to the PEG4 reference.

Taken together, we successfully developed new types of maleimide linkers with distinctly increased aqueous solubility, that expand the modern medicinal chemist’s toolbox for the development of thiol-targeting therapeutics.

## Experimental Section

### Materials and Methods

Potassium tetrachloridoplatinate was purchased from Johnson Matthey (Switzerland). Water for synthesis was taken from a reverse osmosis system and distilled twice before use. For HPLC measurements, Milli-Q water (18.2 MΩ·cm, Merck Milli-Q Advantage, Darmstadt, Germany) was used. H_2_N-ValCitPAB–OH and other chemicals and solvents were purchased from commercial suppliers (Sigma-Aldrich, Merck, Acros, Fluka, and Fisher Scientific). PMal-H,^46^ oxaliplatin, Ox(OH)_2_, Ox–OH-OAc^36,37^ and Mal-PEG4-ValCitPAB–OH^45^ were synthesized as described in the literature. Ibuprofen anhydride was synthesized via an adapted literature procedure.^41^ Reactions were conducted under atmospheric conditions and at room temperature (RT) unless stated otherwise. Electrospray ionization mass spectra were recorded on a Bruker amazon SL ion trap mass spectrometer at the Mass Spectrometry Centre of the University of Vienna in the positive and/or negative mode by direct infusion. One- and two-dimensional ^1^H NMR and ^13^C NMR spectra were recorded on a Bruker AV Neo 500 or AV III 600 spectrometer at 298 K. For ^1^H and ^13^C NMR spectra, the solvent residual peak was taken as an internal reference. The ^1^H NMR spectra of all novel compounds and additional ^13^C NMR spectra of the PMal-X-PIP ligands (X = O, C_5_), final platinum(IV) complexes and final Mal-ValCit linkers are depicted in Figures S3–S37. Purification by preparative reverse phase (RP) HPLC was performed on an Agilent 1200 series system using a Waters XBridge C18 column (19 mm × 250 mm). Elemental analysis measurements were carried out on a PerkinElmer 2400 CHN elemental analyzer at the Microanalytical Laboratory of the University of Vienna and are within ±0.4%, confirming >95% purity. The content of TFA and water can vary between different batches of the same compound.

### Synthesis

#### General Procedure A: Synthesis of PMal-X-Br

PMal-H was dissolved in DMF (0.1 mM) at RT and K_2_CO_3_ (5 equiv) was added. A solution of Br-X-Br in DMF (2 mM) was added dropwise over 30 min with the aid of a syringe pump. After final addition, the reaction mixture was left stirring at 50 °C for an additional 1.5 h. DMF was removed under high vacuum at 50 °C and the remaining crude was partitioned between demi-H_2_O and ethyl acetate (EtOAc). The total organic layer was collected, dried over Mg_2_SO_4_, vacuum filtrated and concentrated under reduced pressure.

#### General Procedure B: Synthesis of PMal-X-PIP-NHBoc

PMal-X-Br (2 equiv) was dissolved in dry DMF (1 mM) under argon at RT. 1-(2-Boc-aminoethyl)-piperazine (1 equiv) and TEA (5 equiv) were added and the resulting mixture was stirred for 24 h at RT. DMF was removed under high vacuum and the crude partitioned between demi-H_2_O and DCM. The total organic layer was dried over Mg_2_SO_4_, vacuum filtrated and concentrated under reduced pressure.

#### General Procedure C: Synthesis of PMal-X-PIP-NH_2_·3HCl

PMal-X-PIP-NHBoc was dissolved in 1.25 M HCl in EtOH (0.05 mM) and stirred overnight at RT. Solvent was removed under reduced pressure and the remaining solids were dried for another 24 h under high vacuum.

#### General Procedure D: Syntheses of Ox-OAc-PIP-X-PMal or Ox-OAc-PEG4-PMal

PMal-PEG4-NH_2_·HCl or PMal-X-PIP-NH_2_·3HCl was neutralized with triethylamine (10 equiv) in EtOAc (0.5 mM). The solids were filtered off and the filtrate was concentrated under reduced pressure to afford PMal-PEG4-NH_2_ or PMal-X-Pip-NH_2_ which was used in the next step without further purification. Ox-OAc–OH was dissolved in dry DMF (0.5 mM) under argon at RT. Subsequently, DSC (1.2 equiv) was added and the reaction mixture was left stirring for 5 h at RT in the dark. PMal-PEG4-NH_2_ or PMal-X-PIP-NH_2_ (1.7 equiv) was added and the mixture was stirred for another 24 h at RT in the dark, after which DMF was removed under high vacuum at 40 °C.

#### General Procedure E: Synthesis of Ox-Ibu-PIP-X-PMal or Ox-Ibu-PEG4-PMal

PMal-PEG4-NH_2_·HCl or PMal-X-PIP-NH_2_·3HCl was neutralized with TEA (10 equiv) in EtOAc (0.5 mM). The solids were filtered off and the filtrate was concentrated under reduced pressure to afford PMal-PEG4-NH_2_ or PMal-X-PIP-NH_2_ which was used in the next step without further purification. Ox-IBu- was dissolved in dry DMF (0.5 mM) under Argon at RT. Subsequently, PMal-PEG-NH_2_ or PMal-X-PIP-NH_2_ (1.7 equiv) was added and the mixture was stirred for another 24 h at RT in the dark, after which DMF was removed under high vacuum at 40 °C.

#### General Procedure K: Deprotection of 2,5-Dimethylfurane Protected Maleimides

Substrate was dissolved in DMSO (0.5 mM) and stirred at 90 °C in open air with hourly monitoring of conversion via HPLC. After completion, DMSO was thoroughly removed under high vacuum (50 °C) and the crude product was purified via preparative RP HPLC.

##### 2-(2-Bromoethyl)-4,7-dimethyl-3a,4,7,7a-tetrahydro-1*H*-4,7-epoxyisoindole-1,3(2*H*)-dione (PMal-C_2_–Br)

Synthesized according to general procedure A based on PMal-H (0.99 g, 5.11 mmol). The crude product was purified by silica flash column chromatography (1.5:98.5 acetone:DCM) to afford the title compound as a light yellow crystalline solid (1.02 g, 67%). ^1^H NMR (500 MHz, DMSO-*d*_6_) δ 6.38 (s, 2H, = C*H*_Furane_), 3.80–3.72 (t, *J* = 6.4 Hz, 2H, C*H*_2_N(CO)_2_), 3.58–3.51 (t, *J* = 6.4 Hz, 2H, C*H*_2_Br), 2.93 (s, 2H, C*H*_Furane_), 1.55 (s, 6H, C*H*_3,Furane_) ppm. MS (*m*/*z*): calcd. C_12_H_15_BrNO_3_ (M + H)^+^, 300.02; found, 300.04.

##### 2-(2-(2-Bromoethoxy)ethyl)-4,7-dimethyl-3a,4,7,7a-tetrahydro-1*H*-4,7-epoxyisoindole-1,3(2*H*)-dione (PMal–O-Br)

Synthesized according to general procedure A based on PMal-H (1.47 g, 7.62 mmol). The crude product was purified by silica flash column chromatography (5:95 acetone:DCM) to afford the title compound as a clear yellow oil (1.67 g, 64%). ^1^H NMR (500 MHz, DMSO-*d*_6_) δ 6.36 (s, 2H, = C*H*_Furane,exo_), 6.24 (s, 2H, = C*H*_Furane,endo_), 3.69–3.64 (m, 2H, C*H*_2_N(CO)_2_), 3.54–3.48 (m, 6H, C*H*_PEG_ + C*H*_2_Br), 3.28 (s, 2H, C*H*_Furane,endo_), 2.90 (s, 2H, C*H*_Furane,exo_), 1.63 (s, 6H, C*H*_3,Furane,endo_), 1.53 (s, 6H, C*H*_3,Furane,exo_) ppm. MS (*m*/*z*): calcd. C_14_H_18_BrNO_4_Na (M + Na)^+^, 366.03; found, 366.08.

##### 2-(5-Bromopentyl)-4,7-dimethyl-3a,4,7,7a-tetrahydro-1*H*-4,7-epoxyisoindole-1,3(2*H*)-dione (PMal-C_5_–Br)

Synthesized according to general procedure A based on PMal-H (995 mg, 5.15 mmol). The crude product was purified by silica flash column chromatography (2:98 acetone:DCM) to afford the title compound as a clear yellow oil (1.01 g, 62%). ^1^H NMR (500 MHz, DMSO-*d*_6_) δ 6.36 (s, 2H, = C*H*_Furane,exo_), 3.50–3.45 (t, *J* = 6.6 Hz, 2H, C*H*_2_N(CO)_2_), 3.38–3.31 (m, 2H, C*H*_2_Br, under H_2_O peak), 2.87 (s, 2H, C*H*_Furane,exo_), 1.82–1.74 (p, *J* = 7.1 Hz, 2H, C*H*_2_), 1.53 (s, 6H, C*H*_3,Furane,exo_), 1.50–1.42 (p, *J* = 7.2 Hz, 2H, C*H*_2_), 1.35–1.27 (p, *J* = 7.6 Hz, 2H, C*H*_2_) ppm. MS (*m*/*z*): calcd. C_15_H_20_BrNO_3_ (M + Na)^+^, 364.05; found, 364.07.

##### *tert*-Butyl(2-(4-(2-(4,7-dimethyl-1,3-dioxo-1,3,3a,4,7,7a-hexahydro-2*H*-4,7-epoxyisoindol-2-yl)ethyl)piperazin-1-yl)ethyl)carbamate (PMal-C_2_–PIP-NHBoc)

Synthesized according to general procedure B based on PMal-C_2_–Br (900.45 mg, 3 mmol). The crude product was purified by silica flash column chromatography (5:95 MeOH:DCM) to afford the title compound as a colorless wax (386 mg, 52%). ^1^H NMR (500 MHz, DMSO-*d*_6_) δ 6.66–6.57 (m, 1H, N*H*_Ca_), 6.36 (s, 2H, = C*H*_Furane_), 3.45–3.39 (t, *J* = 6.48 Hz, 2H, C*H*_2_N(CO)_2_), 3.04–2.95 (m, 2H, C*H*_2_NH_Ca_), 2.87 (s, 2H, C*H*_Furane_), 2.44–2.28 (m, 12H, C*H*_2_N_Pip_), 1.54 (s, 6H, C*H*_3,Furane_), 1.36 (s, 9H, C*H*_3,tBu_) ppm. MS (*m*/*z*): calcd. C_23_H_37_N_4_O_5_ (M + H)^+^, 449.28; found, 449.17.

##### *tert*-Butyl(2-(4-(2-(2-(4,7-dimethyl-1,3-dioxo-1,3,3a,4,7,7a-hexahydro-2*H*-4,7-epoxyisoindol-2-yl)ethoxy)ethyl)piperazin-1-yl)ethyl)carbamate (PMal-O-PIP-NHBoc)

Synthesized according to general procedure B based on PMal–O-Br (658 mg, 1.91 mmol). The crude product was purified by silica flash column chromatography (8:92 MeOH:DCM) to afford the title compound as a colorless wax (306 mg, 65%). ^1^H NMR (500 MHz, DMSO-*d*_6_) δ 6.64–6.58 (m, 1H, N*H*_Ca_), 6.36 (s, 2H, = C*H*_Furane_), 3.52–3.47 (t, *J* = 5.72 Hz, 2H, C*H*_2_N(CO)_2_), 3.45–3.40 (m, 4H, C*H*_PEG_), 3.02–2.95 (m, 2H, C*H*_2_NH_Ca_), 2.88 (s, 2H, C*H*_Furane_), 2.41–2.22 (m, 12H, C*H*_2_N_Pip_), 1.53 (s, 6H, C*H*_3,Furane_), 1.36 (s, 9H, C*H*_3,tBu_) ppm. ^13^C NMR (126 MHz, DMSO-*d*_6_) δ 174.8 (2x*C*O_Mal_), 155.5 (*C*O_Ca,Boc_), 140.6 (2x = *C*H_Furane_), 87.9 (2x*C*H_q,furane_), 77.4 (*C*_q,Boc_), 66.4 (2x*C*H_PEG_), 57.3 (2x*C*H_2_N_Pip_), 57.2 (2x*C*H_2_N_Pip_), 53.1 (*C*H_2_N_Pip_), 52.7 (*C*H_2_N_Pip_), 52.1 (2x*C*H_Furane_), 37.4 (*C*H_2_NH_Ca,Boc_ + *C*H_2_N(CO)_2_), 28.2 (*C*H_3,tBu_), 15.6 (2x*C*H_3,furane_) ppm. MS (*m*/*z*): calcd. C_25_H_40_N_4_O_6_ (M + H)^+^, 493.30; found, 493.32.

##### *tert*-Butyl(2-(4-(5-(4,7-dimethyl-1,3-dioxo-1,3,3a,4,7,7a-hexahydro-2*H*-4,7-epoxyisoindol-2-yl)pentyl)piperazin-1-yl)ethyl)carbamate (PMal-C_5_–PIP-NHBoc)

Synthesized according to general procedure B based on PMal-C_5_–Br (440 mg, 1.28 mmol). The crude product was purified by silica flash column chromatography (4:96 MeOH:DCM) to afford the title compound as a colorless wax (220 mg, 73%). ^1^H NMR (500 MHz, DMSO-*d*_6_) δ 6.71–6.57 (m, 1H, N*H*_Ca_), 6.37 (s, 2H, = C*H*_Furane_), 3.38–3.31 (m, 2H, C*H*_2_N(CO)_2_, under H_2_O peak), 3.07–2.95 (m, 2H, C*H*_2_NH_Ca_), 2.87 (s, 2H, C*H*_Furane_), 2.41–2.22 (m, 12H, C*H*_2_N_Pip_), 1.53 (s, 6H, C*H*_3,Furane_), 1.49–1.33 (m, 13H, C*H*_2_ + C*H*_3,tBu_), 1.22–1.14 (m, 2H, C*H*_2_) ppm. ^13^C NMR (126 MHz, DMSO-*d*_6_) δ 174.9 (2x*C*O_Mal_), 155.5 (*C*O_Ca,Boc_), 140.6 (2x = *C*H_Furane_), 86.8 (2x*C*H_q,furane_), 77.5 (*C*_q,Boc_), 55.8 (3x*C*H_2_N_Pip_), 54.9 (3x*C*H_2_N_Pip_), 52.1 (2x*C*H_Furane_), 37.4 (*C*H_2_NH_Ca,Boc_ + *C*H_2_N(CO)_2_), 28.1 (*C*H_3,tBu_), 26.8 (2x*C*H_2_), 23.6 (*C*H_2_), 15.7 (2x*C*H_3,furane_) ppm. MS (*m*/*z*): calcd. C_26_H_42_N_4_O_5_ (M + H)^+^, 491.67; found, 491.71.

##### 2-(2-(4-(2-Aminoethyl)piperazin-1-yl)ethyl)-4,7-dimethyl-3a,4,7,7a-tetrahydro-1*H*-4,7-epoxyisoindole-1,3(2*H*)-dione Trihydrochloride (PMal-C_2_–PIP-NH_2_·3HCl)

Synthesized according to general procedure C based on PMal-C_2_–PIP-NHBoc (390 mg, 0.87 mmol). The title compound was obtained as an off-white solid (393 mg, 99%). ^1^H NMR (500 MHz, D_2_O) δ 6.45 (s, 2H, = C*H*_Furane_), 3.95–3.85 (t, *J* = 6.3 Hz, 2H, C*H*_2_N(CO)_2_), 3.45–3.27 (m, 6H, C*H*_pip_), 3.18 (s, 2H, C*H*_Furane_), 3.17–3.11 (t, *J* = 6.2 Hz, 2H, C*H*_2_NH_2_), 3.01–2.76 (m, 6H, C*H*_2_N_Pip_), 1.63 (s, 6H, C*H*_3,Furane_) ppm. MS (*m*/*z*): calcd. C_18_H_29_N_4_O_3_ (M + H)^+^, 349.22; found, 349.25.

##### 2-(2-(2-(4-(2-Aminoethyl)piperazin-1-yl)ethoxy)ethyl)-4,7-dimethyl-3a,4,7,7a-tetrahydro-1*H*-4,7-epoxyisoindole-1,3(2*H*)-dione Trihydrochloride (PMal-O-PIP-NH_2_·3HCl)

Synthesized according to general procedure C based on PMal-O-PIP-NHBoc (200.2 mg, 0.41 mmol). The title compound was obtained as an off-white solid (195 mg, 95%). ^1^H NMR (500 MHz, D_2_O) δ 6.45 (s, 2H, = C*H*_Furane,exo_), 6.45 (s, 2H, = C*H*_Furane,endo_), 3.80–3.74 (t, *J* = 5.0 Hz, 2H, C*H*_2_N(CO)_2_), 3.71–3.67 (t, *J* = 5.0 Hz, 2H, C*H*_PEG_), 3.66–3.62 (t, *J* = 4.8 Hz, 2H, C*H*_PEG_), 3.46–3.24 (m, 6H, C*H*_2_N_Pip_), 3.20–3.07 (m, 4H, C*H*_Furane_ + C*H*_2_NH_2_), 3.03–2.71 (m, 6H, C*H*_2_N_Pip_), 1.74 (s, 6H, C*H*_3,Furane,endo_), 1.63 (s, 6H, C*H*_3,Furane,exo_) ppm. ^13^C NMR (126 MHz, D_2_O) δ 177.8 (2x*C*O_Mal_), 140.5 (2x = *C*H_Furane_), 88.0 (2x*C*H_q,furane_), 67.6 (*C*H_PEG_), 63.8 (*C*H_PEG_), 57.4 (3x*C*H_2_N_Pip_), 56.0 (3x*C*H_2_N_Pip_), 52.4 (2x*C*H_Furane_), 38.2 (*C*H_2_NH_2_)_,_ 35.0 (*C*H_2_N(CO)_2_), 15.1 (2x*C*H_3,furane_) ppm MS (*m*/*z*): calcd. C_20_H_32_N_4_O_4_ (M + H)^+^, 393.25; found, 393.25.

##### 2-(2-(2-(4-(2-Aminoethyl)piperazin-1-yl)ethoxy)ethyl)-4,7-dimethyl-3a,4,7,7a-tetrahydro-1*H*-4,7-epoxyisoindole-1,3(2*H*)-dione Trihydrochloride (PMal-C_5_–PIP-NH_2_·3HCl)

Synthesized according to general procedure C based on PMal-C_5_–PIP-NHBoc (220 mg, 0.45 mmol). The title compound was obtained as an off-white crystalline solid (214 mg, 95%). ^1^H NMR (500 MHz, D_2_O) δ 6.44 (s, 2H, = C*H*_Furane,exo_), 3.53–2.63 (m, 18H, C*H*_2_N(CO)_2_ + C*H*_2_N_Pip_ + C*H*_Furane,exo_ + C*H*_2_NH_2_), 1.76–1.66 (m, 2H, C*H*_2_), 1.65–1.53 (m, 8H, C*H*_3,Furane,exo_ + C*H*_2_), 1.33–1.22 (m, 2H, C*H*_2_) ppm. ^13^C NMR (126 MHz, D_2_O) δ 180 (2x*C*O_Mal_), 140.5 (2x = *C*H_Furane_), 88.0 (2x*C*H_q,furane_), 56.5 (6x*C*H_2_N_Pip_), 52.3 (2x*C*H_Furane_), 38.0 (*C*H_2_NH_2_ + *C*H_2_N(CO)_2_), 35.1 (*C*H_2_), 26.2 (*C*H_2_), 22.8 (*C*H_2_), 15.0 (2x*C*H_3,furane_) ppm MS (*m*/*z*): calcd. C_21_H_34_N_4_O_3_ (M + H)^+^, 391.27; found, 391.29.

##### *tert*-Butyl(14-(4,7-dimethyl-1,3-dioxo-1,3,3a,4,7,7a-hexahydro-2*H*-4,7-epoxyisoindol-2-yl)-3,6,9,12-tetraoxatetradecyl)carbamate (PMal-PEG4-NHBoc)

tert-Butyl(14-amino-3,6,9,12-tetraoxatetradecyl)carbamate (1.3 g, 3.8 mmol) was dissolved in saturated NaHCO_3_ solution (35 mL) and cooled down to 0 °C in an ice bath. Methyl 2,5-dioxo-2,5-dihydro-1*H*-pyrrole-1-carboxylate (1000 mg, 6.5 mmol) was added and the solution was stirred for 15 min at 0 °C, then for 60 min at RT. Demi-H_2_O was added (70 mL), and the aqueous phase was extracted with CHCl_3_ (3 × 200 mL). The total organic phase was dried over Mg_2_SO_4_, vacuum filtrated and concentrated under reduced pressure to afford Mal-PEG4-NHBoc as a colorless oil, which was used in the next step without further analysis and purification. Mal-PEG4-NHBoc was dissolved in acetonitrile (5 mL), dimethylfurane (4.1 mL, 38 mmol) was added and the mixture was stirred for 24 h at 60 °C. The solvent was removed under reduced pressure and the crude purified by silica flash column chromatography (3:97 MeOH:DCM) to afford the title compound as a clear, yellow oil (1.53 g, 79%). ^1^H NMR (500 MHz, DMSO-*d*_6_) δ 6.81–6.71 (m, 1H, N*H*_Ca_), 6.36 (s, 2H, = C*H*_Furane,exo_), 6.23 (s, 2H, = C*H*_Furane,endo_), 3.53–3.35 (m, 18H, C*H*_PEG_), 3.27 (s, 2H, C*H*_Furane,endo_), 3.07–3.02 (m, 2H, C*H*_2_NH_Ca_), 2.89 (s, 2H, C*H*_Furane,exo_), 1.62 (s, 6H, C*H*_3,Furane,endo_), 1.53 (s, 6H, C*H*_3,Furane,exo_), 1.37 (s, 9H, C*H*_3,tBu_) ppm. ^13^C NMR (126 MHz, DMSO-*d*_6_) δ 174.8 (2x*C*O_Mal_), 155.5 (*C*O_Ca,Boc_), 140.6 (2x = *C*H_Furane_), 86.9 (2x*C*H_q,furane_), 77.6 (*C*_q,Boc_), 69.8 (2x*C*H_PEG_), 69.7 (2x*C*H_PEG_), 69.5 (*C*H_PEG_), 69.4 (*C*H_PEG_), 69.3 (*C*H_PEG_), 69.1 (*C*H_PEG_), 66.4 (*C*H_PEG_), 66.2 (*C*H_PEG_), 52.1 (2x*C*H_Furane_), 37.4 (*C*H_2_NH_Ca,Boc_), 37.1 (*C*H_2_N(CO)_2_), 28.2 (*C*H_3,tBu_), 15.6 (2x*C*H_3,furane_) ppm. MS (*m*/*z*): calcd. C_25_H_40_N_2_O_9_Na (M + Na)^+^, 535.26; found, 535.28.

##### 2-(14-Amino-3,6,9,12-tetraoxatetradecyl)-4,7-dimethyl-3a,4,7,7a-tetrahydro-1*H*-4,7-epoxyisoindole-1,3(2*H*)-dione (PMal-PEG4-NH_2_·HCl)

PMal-PEG4-NHBoc (312.9 mg, 0.61 mmol) was dissolved in 1.25 M HCl in EtOH (12 mL) and stirred at room temperature for 4 h. The solvent was removed thoroughly under reduced pressure to afford the title compound as a clear, light brown oil (248 mg, 91%). ^1^H NMR (500 MHz, DMSO-*d*_6_) δ 6.37 (s, 2H, = C*H*_Furane,exo_), 6.22 (s, 2H, = C*H*_Furane,endo_), 3.61–3.58 (t, *J* = 5.6 Hz, 2H, C*H*_2_N(CO)_2_), 3.57–3.44 (m, 16H, C*H*_PEG_), 3.28 (s, 2H, C*H*_Furane,endo_), 3.00–2.92 (m, 2H, C*H*_2_NH_2_), 2.89 (s, 2H, C*H*_Furane,exo_), 1.63 (s, 6H, C*H*_3,Furane,endo_), 1.53 (s, 6H, C*H*_3,Furane,exo_) ppm. ^13^C NMR (126 MHz, DMSO-d6) δ 174.8 (2x*C*O_Mal_), 140.6 (2x = *C*H_Furane_), 86.9 (2x*C*H_q,furane_), 69.8 (*C*H_PEG_), 69.7 (3x*C*H_PEG_), 69.6 (*C*H_PEG_), 69.4 (*C*H_PEG_), 69.3 (*C*H_PEG_), 66.6 (*C*H_PEG_), 66.5 (*C*H_PEG_), 66.2 (*C*H_PEG_), 52.1 (2x*C*H_Furane_), 38.5 (*C*H_2_NH_2_), 37.4 (*C*H_2_N(CO)_2_), 15.7 (2x*C*H_3,furane_) ppm. MS (*m*/*z*): calcd. C_20_H_33_N_2_O_7_ (M + H)^+^, 413.49; found, 413.52.

##### (OC-6–34)-Acetato-[(1*R*,2*R*)-1,2-cyclohexanediamino][(2-(4-(2-(4,7-dimethyl-1,3-dioxo-1,3,3a,4,7,7a-hexahydro-2*H*-4,7-epoxyisoindol-2-yl)ethyl)piperazin-1-yl)ethyl)carbamato]oxalatoplatinum(IV) (Ox-OAc-PIP-C_2_–PMal)

Synthesized according to general procedure D based on Ox-OAc–OH (31 mg, 0.065 mmol). The crude product was purified via preparative RP-HPLC (0–0.5 min A:B 95:5, 0.5–15 min linear gradient to A:B 50:50, 15–18 min A:B 5:95, 18–21 min A:B 95:5/A:H_2_O (+0.1% CF_3_COOH), B:MeCN (+0.1% CF_3_COOH)) and lyophilized to afford the title compound as a white solid (40 mg, 72%). ^1^H NMR (500 MHz, DMSO-*d*_6_) δ 9.19 (m, 1H, N*H*_2_), 8.63–8.15 (m, 3H, N*H*_2_), 7.11–6.90 + 6.47–6.38 (m, 1H, N*H*_Ca_), 6.37 (s, 2H, = C*H*_Furane_), 3.31–2.60 (m, 18H, C*H*_2_N(CO)_2_ + C*H*_2_NH_Ca_ + C*H*_2_N_Pip_ + C*H*_Furane_), 2.59–2.50 (m, 2H, C*H*_cHex_, under DMSO peak), 2.19–2.07 (m, 2H, C*H*_cHex_), 1.96 (s, 3H, PtOCOC*H*_3_), 1.58–1.29 (m, 10H, C*H*_cHex_ + C*H*_3,Furane_), 1.25–1.04 (m, 2H, C*H*_cHex_) ppm. MS (*m*/*z*): calcd. C_29_H_45_N_6_O_11_Pt (M + H)^+^, 848.27; found, 848.17.

##### (OC-6–34)-Acetato-[(1*R*,2*R*)-1,2-cyclohexanediamino][(2-(4-(2-(2-(4,7-dimethyl-1,3-dioxo-1,3,3a,4,7,7a-hexahydro-2*H*-4,7-epoxyisoindol-2-yl)ethoxy)ethyl)piperazin-1-yl)ethyl)carbamato]oxalatoplatinum(IV) (Ox-OAc-PIP-O-PMal)

Synthesized according to general procedure D based on Ox-OAc–OH (45 mg, 0.093 mmol). The crude product was purified via preparative RP HPLC (14% MeCN (+0.1% CF_3_COOH) in H_2_O (+0.1% CF_3_COOH); isocratic) and lyophilized to afford the title compound as a white solid (61 mg, 72%). ^1^H NMR (500 MHz, DMSO-*d*_6_) δ 9.63–9.20 (m, 1H, N*H*_2_), 8.66–8.45 (bs, 1H, N*H*_2_), 8.41–8.13 (m, 2H, N*H*_2_), 6.93–6.76 (bs, 1H, N*H*_Ca_), 6.38 (s, 2H, = C*H*_Furane, exo_), 6.24 (s, 2H, = C*H*_Furane,endo_), 3.67–2.87 (m, 22H, C*H*_2_N(CO)_2_ + C*H*_2_NH_Ca_ + C*H*_PEG_ + C*H*_2_N_Pip_ + C*H*_Furane_), 2.62–2.53 (m, 2H, C*H*_cHex_), 2.18–2.07 (m, 2H, C*H*_cHex_), 1.95 (s, 3H, PtOCOC*H*_3_), 1.63 (s, 2H, C*H*_3,Furane,endo_), 1.58–1.29 (m, 10H, C*H*_cHex_ + C*H*_3,Furane,exo_), 1.26–1.06 (m, 2H, C*H*_cHex_) ppm. MS (*m*/*z*): calcd. C_31_H_49_N_6_O_12_Pt (M + H)^+^, 892.31; found, 892.34.

##### (OC-6–34)-Acetato-[(1*R*,2*R*)-1,2-cyclohexanediamino][(2-(4-(5-(4,7-dimethyl-1,3-dioxo-1,3,3a,4,7,7a-hexahydro-2*H*-4,7-epoxyisoindol-2-yl)pentyl)piperazin-1-yl)ethyl)carbamato]oxalatoplatinum(IV) (Ox-OAc-PIP-C_5_–PMal)

Synthesized according to general procedure D based on Ox-OAc–OH (50 mg, 0.1 mmol). The crude product was purified via preparative RP-HPLC (0–0.5 min A:B 95:5, 0.5–11 min linear gradient to A:B 5:85, 11–14 min A:B 5:95, 14–17 min A:B 95:5/A:H_2_O (+0.1% CF_3_COOH), B:MeCN (+0.1% CF_3_COOH)) and lyophilized to afford the title compound as a white solid (69 mg, 73%). ^1^H NMR (500 MHz, DMSO-*d*_6_) δ 9.74–9.13 (m, 1H, N*H*_2_), 8.70–8.47 (bs, 1H, N*H*_2_), 8.41–8.12 (m, 2H, N*H*_2_), 6.90–6.67 (bs, 1H, N*H*_Ca_), 6.38 (s, 2H, = C*H*_Furane_), 3.67–2.87 (m, 18H, C*H*_2_N(CO)_2_ + C*H*_2_NH_Ca_ + C*H*_2_N_Pip_ + C*H*_Furane_), 2.64–2.52 (m, 2H, C*H*_cHex_), 2.18–2.07 (m, 2H, C*H*_cHex_), 1.96 (s, 3H, PtOCOC*H*_3_), 1.65–1.56 (m, 2H, C*H*_cHex_), 1.55–1.29 (m, 11H, C*H*_cHex_ + C*H*_2_ + C*H*_3,Furane_), 1.39–1.30 (m, 1H, C*H*_cHex_), 1.27–1.07 (m, 4H, C*H*_cHex_ + C*H*_2_) ppm. MS (*m*/*z*): calcd. C_31_H_49_N_6_O_12_Pt (M + H)^+^, 892.31; found, 892.34.

##### (OC-6–34)-Acetato-[(1*R*,2*R*)-1,2-cyclohexanediamino][(14-(4,7-dimethyl-1,3-dioxo-1,3,3a,4,7,7a-hexahydro-2*H*-4,7-epoxyisoindol-2-yl)-3,6,9,12-tetraoxatetradecyl)carbamato]oxalatoplatinum(IV) (Ox-OAc-PEG4-PMal)

Synthesized according to general procedure D based on Ox-OAc–OH (45.9 mg, 0.097 mmol). The crude product was purified via preparative RP-HPLC (0–0.5 min A:B 95:5, 0.5–15 min linear gradient to A:B 50:50, 15–18 min A:B 5:95, 18–21 min A:B 95:5/A:H_2_O (+0.1% CF_3_COOH), B:MeCN (+0.1% CF_3_COOH)) and lyophilized to afford the title compound as a white solid (60.2 mg, 68%). ^1^H NMR (500 MHz, DMSO-*d*_6_) δ 9.77–9.34 (m, 1H, N*H*_2_), 8.67–8.49 (m, 1H, N*H*_2_), 8.37–8.15 (m, 2H, N*H*_2_), 6.77–6.67 + 6.34–6.29 (m, 1H, N*H*_Ca_), 6.37 (s, 2H, = C*H*_Furane,exo_), 6.22 (s, 2H, = C*H*_Furane,endo_), 3.51–3.31 (m, 18H, C*H*_2_N(CO)_2_ + C*H*_PEG_, under H_2_O peak), 3.27 (s, 2H, C*H*_Furane,endo_), 3.11–2.99 (m, 2H, C*H*_2_NH_Ca_), 2.89 (s, 2H, C*H*_Furane,exo_), 2.60–2.52 (m, 2H, C*H*_cHex_), 2.17–2.07 (m, 2H, C*H*_cHex_), 1.94 (s, 3H, PtOCOC*H*_3_), 1.62 (s, 2H, C*H*_3,Furane,endo_), 1.56–1.31 (m, 9H, C*H*_cHex_ + C*H*_3,Furane,exo_), 1.24–1.08 (m, 2H, C*H*_cHex_) ppm. MS (*m*/*z*): calcd. C_31_H_49_N_4_O_15_Pt (M + H)^+^, 912.83; found, 912.85.

##### (OC-6–34)-Acetato-[(1*R*,2*R*)-1,2-cyclohexanediamino][(2-(4-(2-(2,5-dioxo-2,5-dihydro-1*H*-pyrrol-1-yl)ethyl)piperazin-1-yl)ethyl)carbamato]oxalatoplatinum(IV) (Ox-OAc-PIP-C_2_-Mal)

Synthesized according to general procedure K based on Ox-OAc-PIP-C_2_–PMal (27.3 mg, 0.032 mmol) with 3 h of stirring. The crude was purified via preparative RP HPLC (0–0.5 min A:B 95:5, 0.5–15 min linear gradient to A:B 72:28, 15–18 min A:B 5:95, 18–21 min A:B 95:5/A:H_2_O (+0.1% CF_3_COOH), B:MeCN (+0.1% CF_3_COOH)) and lyophilized to afford the title compound as a white solid (15 mg, 60%). ^1^H NMR (500 MHz, DMSO-*d*_6_) δ 9.40–9.07 (m, 1H, N*H*_2_), 8.59–8.44 (bs, 1H, N*H*_2_), 8.41–8.16 (m, 2H, N*H*_2_), 7.04 (s, 2H, C*H*_Mal_), 7.01–6.88 + 6.45–6.36 (m, 1H, N*H*_Ca_), 3.68–3.54 (m, 2H, C*H*_2_N(CO)_2_), 3.49–2.66 (m, 14H,C*H*_2_NH_Ca_ + C*H*_2_N_Pip_), 2.66–2.53 (m, 2H, C*H*_cHex_), 2.20–2.08 (m, 2H, C*H*_cHex_), 1.96 (s, 3H, PtOCOC*H*_3_), 1.56–1.29 (m, 4H, C*H*_cHex_), 1.22–1.09 (m, 2H, C*H*_cHex_) ppm. ^13^C NMR* (126 MHz, DMSO-d6) δ 178.2 (PtO*C*OCH_3_), 171.0 (2x*C*O_Mal_), 164.3 (*C*O_Ox_), 163.4 (*C*O_Ox_), 163.3 (*C*O_Ca_), 134.8 (2x*C*H_Mal_), 61.2 (*C*H_cHex_), 60.8 (*C*H_cHex_), 55.3* (3xC*H*_2_N_Pip_), 54.0* (3xC*H*_2_N_Pip_), 36.4 (*C*H_2_NH_Ca_, ppm value taken from [^1^H–^13^C] HSQC crosspeak), 33.9 (*C*H_2_N(CO)_2_, ppm value taken from [^1^H–^13^C] HSQC crosspeak), 31.0 (*C*H_cHex_), 30.8 (*C*H_cHex_), 23.6 (*C*H_cHex_), 23.4 (*C*H_cHex_), 22.8 (PtOCO*C*H_3_) ppm. MS (*m*/*z*): calcd. C_23_H_37_N_6_O_10_Pt (M + H)^+^, 752.22; found, 752.25 EA calcd. C_25_H_40_N_4_O_14_Pt·2TFA·1.5H_2_O; C, 32.21; H, 4.1; N, 8.35; found; C, 31.95; H, 3.8; N, 8.73.

##### (OC-6–34)-Acetato-[(1*R*,2*R*)-1,2-cyclohexanediamino][(2-(4-(2-(2-(2,5-dioxo-2,5-dihydro-1*H*-pyrrol-1-yl)ethoxy)ethyl)piperazin-1-yl)ethyl)carbamato]oxalatoplatinum(IV) (Ox-OAc-PIP-O-Mal)

Synthesized according to general procedure K based on Ox-OAc-PIP-O-PMal (12.7 mg, 0.014 mmol) with 2 h of stirring. The crude was purified via preparative RP-HPLC (0–0.5 min A:B 95:5, 0.5–5.5 min linear gradient to A:B 45:55, 5.5–8.5 min A:B 5:95, 8.5–11.5 min A:B 95:5/A:H_2_O (+0.1% HCOOH), B:MeCN (+0.1% HCOOH)) and lyophilized to afford the title compound as a white solid (9.1 mg, 82%). ^1^H NMR (500 MHz, DMSO-*d*_6_) δ 9.55–9.20 (m, 1H, N*H*_2_), 8.64–8.46 (bs, 1H, N*H*_2_), 8.41–8.15 (m, 2H, N*H*_2_), 7.05 (s, 2H, C*H*_mal_), 6.93–6.78 + 6.41–6.31 (m, 1H, N*H*_Ca_), 3.70–3.63 (m, 2H, C*H*_2_N(CO)_2_), 3.62–3.58 (t, 2H, *J* = 5.2 Hz, C*H*_PEG_), 3.57–3.53 (t, 2H, *J* = 5.3 Hz, C*H*_PEG_), 3.46–2.66 (m, 14H, C*H*_2_NH_Ca_ + C*H*_2_N_Pip_), 2.65–2.53 (m, 2H, C*H*_cHex_), 2.19–2.07 (m, 2H, C*H*_cHex_), 1.95 (s, 3H, PtOCOC*H*_3_), 1.56–1.29 (m, 4H, C*H*_cHex_), 1.23–1.08 (m, 2H, C*H*_cHex_) ppm. ^13^C NMR* (126 MHz, DMSO-*d*_6_) δ 178.2 (PtO*C*OCH_3_), 171.1 (2x*C*O_Mal_), 164.3 (*C*O_Ox_), 163.5 (*C*O_Ox_), 163.4 (*C*O_Ca_), 134.7 (2x C*H*_Mal_), 67.3 (C*H*_PEG_), 64.94 *C*H_2_N(CO)_2_, ppm value taken from [^1^H–^13^C] HSQC crosspeak, 61.3 (*C*H_cHex_), 60.8 (*C*H_cHex_), 36.6 (C*H*_2_NH_Ca_ + C*H*_PEG_), 31.0 (*C*H_cHex_), 30.8 (*C*H_cHex_), 23.6 (*C*H_cHex_), 23.4 (*C*H_cHex_), 22.8 (PtOCO*C*H_3_) ppm. MS (*m*/*z*): calcd. C_25_H_40_N_6_O_11_Pt (M + H)^+^, 796.25; found, 796.28. EA calcd. C_25_H_40_N_6_O_11_Pt·2TFA·1.5H_2_O; C, 33.15; H, 4.32; N, 8; found; C, 32.88; H, 4.04; N, 8.09. *Signals belonging to C*H*_2_N_Pip_ did not show up in ^13^C NMR.

##### (OC-6–34)-Acetato-[(1*R*,2*R*)-1,2-cyclohexanediamino][(2-(4-(5-(2,5-dioxo-2,5-dihydro-1*H*-pyrrol-1-yl)pentyl)piperazin-1-yl)ethyl)carbamato]oxalatoplatinum(IV) (Ox-OAc-PIP-C_5_-Mal)

Synthesized according to general procedure K based on Ox-OAc-PIP-C_5_–PMal (11.7 mg, 0.013 mmol) with 3 h of stirring. The crude was purified via preparative RP-HPLC (0–0.5 min A:B 95:5, 0.5–15 min linear gradient to A:B 60:40, 15–18 min A:B 5:95, 18–21 min A:B 95:5/A:H_2_O (+0.1% HCOOH), B:MeCN (+0.1% HCOOH)) and lyophilized to afford the title compound as a an off white solid (6.3 mg, 61%). ^1^H NMR (500 MHz, DMSO-d6) δ 9.59–9.22 (m, 1H, N*H*_2_), 8.64–8.46 (bs, 1H, N*H*_2_), 8.40–8.14 (m, 2H, N*H*_2_), 7.02 (s, 2H, C*H*_Mal_), 6.88–6.76 + 6.41–6.29 (m, 1H, N*H*_Ca_), 3.42–3.38 (t, 2H, *J* = 6.9 Hz, C*H*_2_N(CO)_2_), 3.37–2.64 (m, 14H, C*H*_2_NH_Ca_ + C*H*_2_N_Pip_), 2.62–2.54 (m, 2H, C*H*_cHex_), 2.18–2.08 (m, 2H, C*H*_cHex_), 1.96 (s, 3H, PtOCOC*H*_3_), 1.65–1.56 (m, 2H, C*H*_2_), 1.56–1.31 (m, 6H, C*H*_2_ + C*H*_cHex_), 1.27–1.08 (m, 4H, C*H*_2_ + C*H*_cHex_) ppm. ^13^C NMR (126 MHz, DMSO-*d*_6_) δ 178.3 (PtO*C*OCH_3_), 171.1 (2x*C*O_Mal_), 164.3 (*C*O_Ox_), 163.5 (*C*O_Ox_), 163.4 (*C*O_Ca_), 134.5 (2x C*H*_Mal_), 61.2 (*C*H_cHex_), 60.8 (*C*H_cHex_), 55.14 (C*H*_2_N_Pip_, ppm value taken from [^1^H–^13^C] HSQC crosspeak), 36.8 (C*H*_2_NH_Ca_ + *C*H_2_N(CO)_2_ + C*H*_2_N_Pip_), 31.0 (*C*H_cHex_), 30.8 (*C*H_cHex_), 27.5 (2x*C*H_2_), 23.6 (*C*H_cHex_), 23.4 (*C*H_cHex_), 23.2 (*C*H_2_), 22.9 (PtOCO*C*H_3_) ppm. MS (*m*/*z*): calcd. C_26_H_42_N_6_O_10_Pt (M + H)^+^, 794.27; found, 794.33. EA calcd. C_25_H_40_N_4_O_14_Pt·2.5TFA; C, 34.51; H, 4.16; N, 7.79; found; C, 34.41; H, 4.05; N, 7.73.

##### (OC-6–34)-Acetato-[(1*R*,2*R*)-1,2-cyclohexanediamino][(14-(2,5-dioxo-2,5-dihydro-1*H*-pyrrol-1-yl)-3,6,9,12-tetraoxatetradecyl)carbamato]oxalatoplatinum(IV) (Ox-OAc-PEG4-Mal)

Synthesized according to general procedure K based on Ox-OAc-PEG4-PMal (57 mg, 0.063 mmol) with 3 h of stirring. The crude was purified via preparative RP HPLC (0–0.5 min A:B 95:5, 0.5–15 min linear gradient to A:B 45.55, 15–18 min A:B 5:95, 18–21 min A:B 95:5/A:H_2_O (+0.1% CF_3_COOH), B:MeCN (+0.1% CF_3_COOH)) and lyophilized to afford the title compound as a white solid (35 mg, 68%). ^1^H NMR (500 MHz, DMSO-*d*_6_) δ 9.78–9.38 (m, 1H, N*H*_2_), 8.67–8.51 (m, 1H, N*H*_2_), 8.37–8.16 (m, 2H, N*H*_2_), 7.02 (s, 2H, C*H*_Mal_), 6.74–6.66 + 6.34–6.26 (m, 1H, N*H*_Ca_), 3.58–3.54 (m, 2H, C*H*_2_N(CO)_2_), 3.53–3.49 (m, 2H, C*H*_PEG_), 3.49–3.43 (m, 12H, C*H*_PEG_, under H_2_O peak), 3.37–3.27 (m, 2H, C*H*_PEG_), 3.10–2.96 (m, 2H, C*H*_2_NH_Ca_), 2.61–2.53 (m, 2H, C*H*_cHex_), 2.17–2.08 (m, 2H, C*H*_cHex_), 1.95 (s, 3H, PtOCOC*H*_3_), 1.54–1.46 (m, 2H, C*H*_cHex_), 1.45–1.32 (m, 2H, C*H*_cHex_), 1.21–1.09 (m, 2H, C*H*_cHex_) ppm. ^13^C NMR (126 MHz, DMSO-d6) δ 178.4 (PtO*C*O), 170.9 (2x*C*O_Mal_), 164.2 (*C*O_Ox_), 163.4 (*C*O_Ox_), 163.3 (*C*O_Ca_), 134.6 (2x*C*H_Mal_), 69.8 (C*H*_PEG_), 69.7 (C*H*_PEG_), 69.6 (C*H*_PEG_), 69.5 (C*H*_PEG_), 69.4 (C*H*_PEG_), 69.2 (C*H*_PEG_), 66.9 (2xC*H*_PEG_), 61.2 (*C*H_cHex_), 60.8 (*C*H_cHex_), 40.6 (*C*H_2_NH_Ca_), 36.8 (*C*H_2_N(CO)_2_), 31.0 (*C*H_cHex_), 30.8 (*C*H_cHex_), 29.6 (*C*H(CH_3_)_2_), 23.6 (*C*H_cHex_), 23.5 (*C*H_cHex_), 22.2 (PtOCO*C*H_3_) ppm. MS (*m*/*z*): calcd. C_25_H_40_N_4_O_14_NaPt (M + Na)^+^, 838.21; found, 838.21. EA calcd. C_25_H_40_N_4_O_14_Pt·0.5TFA; C, 35.78; H, 4.68; N, 6.42; found; C, 35.99; H, 4.77; N, 6.68.

##### 2-(4-Isobutylphenyl)propanoic Anhydride (Ibuprofen Anhydride)

Ibuprofen (489.2 mg, 2.37 mmol) and DCC (293.5 mg, 1.42 mmol) were dissolved in DCM (3 mL) and the mixture was stirred for 6 h at RT. Precipitated dicyclohexylurea (DCU) was filtered off and the filtrate was concentrated under reduced pressure. EtOAc (3 mL) was added, residual DCU was filtered off, and the filtrate was again concentrated under reduced pressure (repeated twice) to afford the title compound as an opaque oil (470 mg, quantitative). ^1^H NMR (500 MHz, CDCl_3_) δ 7.08–7.00 (m, 8H, C*H*_Ar_), 3.70–3.62 (m, 2H, C*H*CH_3_), 2.46–2.43 (d, *J* = 7.3 Hz, 4H, C*H*_2_CH), 1.89–1.80 (septet, *J* = 6.7 Hz, 2H, C*H*(CH_3_)_2_), 1.44–1.39 (m, 6H, C*H*_3_), 0.94–0.87 (d, *J* = 6.7 Hz, 12H, C*H*_3,i-Pr_) ppm. MS (*m*/*z*): calcd. C_26_H_34_O_3_Na (M + Na)^+^, 417.24; found, 417.27.

##### (OC-6–34)-[(1*R*,2*R*)-1,2-Cyclohexanediamino]-[2-(4-isobutylphenyl)propanoato]-oxalato-[2,5-dioxopyrrolidin-1-yl hydrogen carbonato]platinum(IV) (Ox-Ibu-NHS)

A solution of ibuprofen anhydride (60,2 mg, 0.15 mmol) in dry DMSO (0.5 mL) was added dropwise over 17 h to a stirring suspension of Ox(OH)_2_ (54.6 mg, 0.127 mmol) in dry DMSO (0.8 mL) under Argon at RT with the aid of a syringe pump. The reaction mixture was stirred for an additional 1 h after final addition, after which DSC (65.1 mg, 0.25 mmol) was added. The total reaction mixture was left stirring for another 2 h at RT, after which the crude was directly purified via preparative RP-HPLC (47% MeCN (+0.1% HCOOH) in H_2_O (+0.1% HCOOH); isocratic) and lyophilized to afford the title compound as a white solid (70.5 mg, 73%). ^1^H NMR (500 MHz, DMSO-*d*_6_) δ 8.64–8.49 (m, 1H, N*H*_2_), 8.36–8.21 (m, 1H, N*H*_2_), 8.08–7.95 (m, 1H, N*H*_2_), 7.94–8.82 (m, 1H, N*H*_2_), 7.18–7.11 (m, 2H, C*H*_Ar_), 7.07–7.00 (m, 2H, C*H*_Ar_), 3.76–3.68 (m, 1H, C*H*CH_3_), 2.72 (s, 4H, C*H*_2,Succ_), 2.40–2.35 (d, *J* = 7.2 Hz, 2H, C*H*_2,ibu_), 2.31–2.05 (m, 3H, C*H*_cHex_), 2.01–1.89 (m, 1H, C*H*_cHex_)_,_ 1.83–1–73 (septet, *J* = 6.7 Hz, 1H, C*H*(CH_3_)_2_), 1.57–1–41 (m, 3H, C*H*_cHex_), 1.34–1.25 (m, 4H, C*H*_3_CH + C*H*_cHex_), 1.18–1–06 (m, 1H, C*H*_cHex_), 1.03–0.89 (m, 1H, C*H*_cHex_), 0.84–0.81 (d, *J* = 6.5 Hz, 6H, C*H*_3,i-Pr_) ppm. MS (*m*/*z*): calcd. C_26_H_35_N_3_O_11_NaPt (M + Na)^+^, 783.18; found, 783.23.

##### (OC-6–34)-[(1*R*,2*R*)-1,2-Cyclohexanediamino]-[2-(4-isobutylphenyl)propanoato]-[(2-(4-(2-(2-(4,7-dimethyl-1,3-dioxo-1,3,3a,4,7,7a-hexahydro-2*H*-4,7-epoxyisoindol-2-yl)ethoxy)ethyl)piperazin-1-yl)ethyl)carbamato]oxalatoplatinum(IV) (Ox-Ibu-PIP-O-PMal)

Synthesized according to general procedure E based on Ox-Ibu-NHS (42.1 mg, 0.055 mmol). The crude product was purified via preparative RP-HPLC (0–0.5 min A:B 95:5, 0.5–15 min linear gradient to A:B 40:60, 11–14 min A:B 5:95, 14–17 min A:B 95:5/A:H_2_O (+0.1% CF_3_COOH), B:MeCN (+0.1% CF_3_COOH)) and lyophilized to afford the title compound as a white solid (40.9 mg, 71%). ^1^H NMR (500 MHz, DMSO-*d*_6_) δ 9.45–9.02 (bs, 1H, N*H*_2_), 8.79–8.56 (m, 1H, N*H*_2_), 8.31–7.97 (m, 2H, N*H*_2_), 7.19–7.14 (m, 2H, C*H*_Ar_), 7.07–7.00 (m, 2H, C*H*_Ar_), 6.94–6.74 (m, 1H, N*H*_Ca_), 6.37 (s, 2H, = C*H*_Furane, exo_), 6.24 (s, 2H, = C*H*_Furane, exo_), 3.78–3.60 (m, 1H, C*H*CH_3_, under H_2_O peak), 3.52–2.90 (m, 22H, C*H*_2_N(CO)_2_ + C*H*_2_NH_Ca_ + C*H*_PEG_ + C*H*_2_N_Pip_ + C*H*_Furane_, under H_2_O peak), 2.41–2.36 (d, *J* = 7.0 Hz, 2H, C*H*_2,ibu_), 2.3–1.94 (m, 4H, C*H*_cHex_), 1.83–1.73 (septet, *J* = 6.7 Hz, 1H, C*H*(CH_3_)_2_), 1.64 (s, 2H, C*H*_3,Furane,endo_), 1.53 (s, 2H, CH_3,Furane,exo_), 1.51–1.42 (C*H*_cHex_), 1.36–1–20 (m, 5H, C*H*_cHex_ + C*H*_3_CH), 1.15–1–04 (m, 1H, C*H*_cHex_), 1.03–0.90 (m, 1H, C*H*_cHex_), 0.88–0.80 (d, *J* = 6.9 Hz, 6H, C*H*_3,i-Pr_) ppm. MS (*m*/*z*): calcd. C_43_H_64_N_6_O_11_Pt (M + H)^+^, 1036.43; found, 1036.33.

##### (OC-6–34)-[(1*R*,2*R*)-1,2-Cyclohexanediamino]-[2-(4-isobutylphenyl)propanoato]-[(2-(4-(5-(4,7-dimethyl-1,3-dioxo-1,3,3a,4,7,7a-hexahydro-2*H*-4,7-epoxyisoindol-2-yl)pentyl)piperazin-1-yl)ethyl)carbamato]oxalatoplatinum(IV) (Ox-Ibu-PIP-C_5_–PMal)

Synthesized according to general procedure E based on Ox-Ibu-NHS (32 mg, 0.042 mmol). The crude product was purified via preparative RP HPLC (40% MeCN (+0.1% CF_3_COOH) in H_2_O (+0.1% CF_3_COOH); isocratic) and lyophilized to afford the title compound as a white solid (38.1 mg, 88%). ^1^H NMR (500 MHz, DMSO-*d*_6_) δ 9.32–9.00 (bs, 1H, N*H*_2_), 8.88–8.57 (m, 1H, N*H*_2_), 8.31–7.97 (m, 2H, N*H*_2_), 7.19–7.14 (m, 2H, C*H*_Ar_), 7.06–7.00 (m, 2H, C*H*_Ar_), 6.91–6.64 + 6.44–6.27 (m, 3H, N*H*_Ca_ + = C*H*_Furane_), 3.74–3.64 (q, 1H, C*H*CH_3_), 3.38–2.85 (m, 18H, C*H*_2_N(CO)_2_ + C*H*_2_NH_Ca_ + C*H*_2_N_Pip_ + C*H*_Furane_, under H_2_O peak), 2.42–2.34 (d, *J* = 7.2 Hz, 2H, C*H*_2,ibu_), 2.3–1.94 (m, 4H, C*H*_cHex_), 1.84–1.73 (septet, *J* = 6.7 Hz, 1H, C*H*(CH_3_)_2_), 1.63–1.39 (m, 12H, C*H*_2_ + C*H*_cHex_ + C*H*_3,Furane_), 1.38–1–16 (m, 7H, C*H*_2_ + C*H*_3_CH), 1.15–1–04 (m, 1H, C*H*_cHex_), 1.03–0.81 (m, 7H, C*H*_cHex_ + C*H*_3,i-Pr_) ppm. MS (*m*/*z*): calcd. C_43_H_64_N_6_O_11_Pt (M + H)^+^, 1036.43; found, 1036.33.

##### (OC-6–34)-[(1*R*,2*R*)-1,2-Cyclohexanediamino]-[2-(4-isobutylphenyl)propanoato]- [(14-(4,7-dimethyl-1,3-dioxo-1,3,3a,4,7,7a-hexahydro-2*H*-4,7-epoxyisoindol-2-yl)-3,6,9,12- tetraoxatetradecyl)carbamato]oxalatoplatinum(IV) (Ox-Ibu-PEG4-PMal)

Synthesized according to general procedure E based on Ox-Ibu-NHS (62 mg, 0.081 mmol). The crude product was purified via preparative RP HPLC (48% MeCN (+0.1% CF_3_COOH) in H_2_O (+0.1% CF_3_COOH); isocratic) and lyophilized to afford the title compound as a white solid (53.5 mg, 62%). ^1^H NMR (500 MHz, DMSO-*d*_6_) 9.60–9.21 (m, 1H, N*H*_2_), 8.83–8.59 (m, 1H, N*H*_2_), 8.27–7.93 (m, 2H, N*H*_2_), 7.19–7.13 (m, 2H, C*H*_Ar_), 7.07–7.00 (m, 2H, C*H*_Ar_), 6.79–6.72 + 6.34–6.27 (m, 1H, N*H*_Ca_), 6.36 (s, 2H, = C*H*_Furane,exo_), 6.22 (s, 2H, = C*H*_Furane,endo_), 3.72–3.64 (m, 1H, C*H*CH_3_), 3.55–3.29 (m, 18H, C*H*_2_N(CO)_2_ + C*H*_PEG,_ under H_2_O peak), 3.27 (s, 2H, C*H*_Furane,endo_), 3.09–3.00 (m, 2H, C*H*_2_NH_Ca_), 2.89 (s, 2H, = C*H*_Furane,exo_), 2.59–2.50 (m, 1H, C*H*_cHex_, under DMSO peak), 2.40–2.34 (m, 2H, C*H*_2,ibu_), 2.26–1.92 (m, 3H, C*H*_cHex_), 1.83–1.73 (septet, *J* = 6.6 Hz, 1H, C*H*(CH_3_)_2_), 1.62 (s, 6H, C*H*_3,Furane,endo_), 1.53 (s, 6H, C*H*_3,Furane,exo_), 1.49–1.41 (m, 2H, C*H*_cHex_), 1.38–1.05 (m, 6H, C*H*_cHex_ + C*H*_3_CH), 1.05–0.88 (m, 1H, C*H*_cHex_), 0.87–0.81 (d, *J* = 6.5 Hz, 6H, C*H*_3,i-Pr_) ppm. MS (*m*/*z*): calcd. C_42_H_62_N_4_O_15_NaPt (M + Na)^+^, 1080.38; found, 1080.37.

##### (OC-6–34)-[(1*R*,2*R*)-1,2-Cyclohexanediamino]-[2-(4-isobutylphenyl)propanoato]-[(2-(4-(2-(2-(2,5-dioxo-2,5-dihydro-1*H*-pyrrol-1-yl)ethoxy)ethyl)piperazin-1-yl)ethyl)carbamato]oxalatoplatinum(IV) (Ox-Ibu-PIP-O-Mal)

Synthesized according to general procedure K based on Ox-Ibu-PIP-O-PMal (40.9 mg, 0.039 mmol) with 2 h of stirring. The crude was purified via preparative RP HPLC (0–0.5 min A:B 95:5, 0.5–15 min linear gradient to A:B 38:62, 15–18 min A:B 5:95, 18–21 min A:B 95:5/A:H_2_O (+0.1% CF_3_COOH), B:MeCN (+0.1% CF_3_COOH)) and lyophilized to afford the title compound as a white solid (33.3 mg, 90%). ^1^H NMR (500 MHz, DMSO-*d*_6_) δ 9.49–9.02 (m, 1H, N*H*_2_), 8.82–8.52 (m, 1H, N*H*_2_), 8.33–7.95 (m, 2H, N*H*_2_), 7.19–7.13 (m, 2H, C*H*_Ar_), 7.07–7.00 (m, 4H, C*H*_Ar_ + C*H*_Mal_), 6.91–6.64 + 6.41–6.25 (m, 1H, N*H*_Ca_), 3.77–3.51 (m, 7H, C*H*_2_N(CO)_2_ + C*H*_PEG_ + C*H*CH_3_), 3.26–2.61 (m, 14H, C*H*_2_N_Pip_ + C*H*_2_NH_Ca_, under H_2_O peak), 2.41–2.33 (d, *J* = 6.0 Hz, 2H, C*H*_2,ibu_), 2.31–1.93 (m, 4H, C*H*_cHex_), 1.84–1.74 (septet, *J* = 6.6. Hz, 1H, C*H*(CH_3_)_2_), 1.52–1.41 (m, 2H, C*H*_cHex_), 1.40–1.18 (m, 5H, C*H*_3_CH + C*H*_cHex_), 1.16–1.05 (m, 1H, C*H*_cHex_), 1.03–0.89 (m, 1H, C*H*_cHex_), 0.88–0.80 (d, *J* = 6.5 Hz, 6H, C*H*_3,i-Pr_) ppm. ^13^C NMR* (126 MHz, DMSO-d6) δ 181.5 (PtO*C*O), 171.0 (2x *C*O_Mal_), 164.3 (*C*O_Ox_), 163.3 (*C*O_Ox_), 163.2 (*C*O_Ca_), 139.2 (*C*_q,Ar_), 139.0 (*C*_q,Ar_), 134.6 (2x*C*H_Mal_), 128.8 (2x*C*H_Ar_), 127.2 (2x*C*H_Ar_), 67.3 (2xC*H*_PEG_), 70.0 (*C*H_cHex_), 60.9 (*C*H_cHex_), 46.0 (*C*HCH_3_), 44.3 (*C*H_2,Ibu_), 36.6 (*C*H_2_NH_Ca_ + *C*H_2_N(CO)_2_), 31.0 (*C*H_cHex_), 30.8 (*C*H_cHex_), 29.6 (*C*H(CH_3_)_2_), 23.6 (*C*H_cHex_), 23.4 (*C*H_cHex_), 22.2 (CH(*C*H_3_)_2_), 19.0 (CH*C*H_3_) ppm. MS (*m*/*z*): calcd. C_36_H_55_N_6_O_11_Pt (M + H)^+^, 942.35; found, 942.33. EA calcd. C_36_H_54_N_6_O_11_Pt·2TFA·1.5H_2_O; C, 40.14; H, 4.97; N, 7.02; found; C, 40.31; H, 4.84; N, 6.9. *Signals belonging to *C*H_2_N_Pip_ did not show up in ^13^C NMR.

##### (OC-6–34)-[(1*R*,2*R*)-1,2-Cyclohexanediamino]-[2-(4-isobutylphenyl)propanoato]-[(2-(4-(5-(2,5-dioxo-2,5-dihydro-1*H*-pyrrol-1-yl)pentyl)piperazin-1-yl)ethyl)carbamato]oxalatoplatinum(IV) (Ox-Ibu-PIP-C_5_-Mal)

Synthesized according to general procedure K based on Ox-Ibu-PIP-C_5_–PMal (38.1 mg, 0.037 mmol) with 3 h of stirring. The crude was purified via preparative RP HPLC (0–11 min linear gradient from A:B 62:38 to A:B 61:39, 11–14 min A:B 5:95, 14–17 min A:B 62.38/A:H_2_O (+0.1% CF_3_COOH), B:MeCN (+0.1% CF_3_COOH)) and lyophilized to afford the title compound as a white solid (28.7 mg, 83%). ^1^H NMR (500 MHz, DMSO-*d*_6_) 9.26–9.10 (m, 1H, N*H*_2_), 8.82–8.57 (m, 1H, N*H*_2_), 8.27–7.98 (m, 2H, N*H*_2_), 7.19–7.14 (m, 2H, C*H*_Ar_), 7.07–7.00 (m, 4H, C*H*_Ar_ + C*H*_Mal_), 6.83–6.63 + 6.38–6.26 (m, 1H, N*H*_Ca_), 3.73–3.66 (q, 1H, C*H*CH_3_), 3.55.3.31 (m, 2H, C*H*_2_N(CO)_2_, under D_2_O peak), 3.26–2.61 (m, 14H, C*H*_2_N_Pip_ + C*H*_2_NH_Ca_, under D_2_O peak), 2.41–2.34 (m, 2H, C*H*_2,ibu_), 2.33–2.07 (m, 3H, C*H*_cHex_), 2.06–1.94 (m, 1H, C*H*_cHex_), 1.82–1.74 (septet, *J* = 6.7 Hz, 1H, C*H*(CH_3_)_2_), 1.65–1.55 (m, 2H, C*H*_cHex_), 1.56–1.41 (m, 4H, C*H*_2_), 1.38–1.18 (m, 7H, C*H*_3_CH + C*H*_cHex_ + C*H*_2_), 1.15–1.04 (m, 1H, C*H*_cHex_), 1.03–0.89 (m, 1H, C*H*_cHex_), 0.87–0.82 (d, *J* = 6.6. Hz, 6H, C*H*_3,i-Pr_) ppm. ^13^C NMR* (126 MHz, DMSO-*d*_6_) δ 181.5 (PtO*C*O), 171.1 (2x *C*O_Mal_), 164.3 (*C*O_Ox_), 163.4 (*C*O_Ox_), 163.2 (*C*O_Ca_), 139.2 (*C*_q,Ar_), 139.2 (*C*_q,Ar_), 134.5 (2x*C*H_Mal_), 128.8 (2x*C*H_Ar_), 127.7 (2x*C*H_Ar_), 60.9 (2x*C*H_cHex_), 46.0 (*C*HCH_3_), 44.3 (*C*H_2,Ibu_), 37.6 (*C*H_2_NH_Ca_, ppm value taken from [^1^H–^13^C] HSQC crosspeak), 36.8 (*C*H_2_N(CO)_2_), 30.9 (*C*H_cHex_), 29.6 (*C*H(CH_3_)_2_), 27.5 (2x*C*H_2_), 23.6 (*C*H_2_), 23.3 (*C*H_cHex_), 22.2 (CH(*C*H_3_)_2_), 19.1 (*C*H_cHex_), 18.9 (CH*C*H_3_) ppm. MS (*m*/*z*): calcd. C_37_H_57_N_6_O_10_Pt (M + H)^+^, 940.38; found, 940.42. EA calcd. C_37_H_56_N_6_O_10_Pt·2TFA·1.5H_2_O; C, 41.21; H, 5.14; N, 7.03; found; C, 41.17; H, 4.94; N, 6.88. *Signals belonging to *C*H_2_N_Pip_ did not show up in ^13^C NMR.

##### (OC-6–34)-[(1*R*,2*R*)-1,2-Cyclohexanediamino]-[2-(4-isobutylphenyl)propanoato]-[14-(2,5-dioxo-2,5-dihydro-1*H*-pyrrol-1-yl)-(3,6,9,12-tetraoxatetradecyl)carbamato]oxalatoplatinum(IV) (Ox-Ibu-PEG4-Mal)

Synthesized according to general procedure K based on Ox-Ibu-PEG4-PMal (52 mg, 0.049 mmol) with 3 h of stirring. The crude was purified via preparative RP-HPLC (47% MeCN (containing 0.1% CF_3_COOH) in H_2_O (containing 0.1% CF_3_COOH); isocratic) and lyophilized to afford the title compound as a white solid (33 mg, 70%).^1^H NMR (500 MHz, DMSO-*d*_6_) 9.62–9.21 (m, 1H, N*H*_2_), 8.83–8.57 (m, 1H, N*H*_2_), 8.26–7.97 (m, 2H, N*H*_2_), 7.19–7.13 (m, 2H, C*H*_Ar_), 7.07–7.00 (m, 4H, C*H*_Ar_ + C*H*_Mal_), 6.78–6.70 + 6.34–6.26 (m, 1H, N*H*_Ca_), 3.73–3.64 (m, 1H, C*H*CH_3_), 3.58–3.43 (m, 16H, C*H*_2_N(CO)_2_ + C*H*_PEG,_ under H_2_O peak), 3–37–3.30 (m 2H, C*H*_PEG_), 3.09–2.93 (m, 2H, C*H*_2_NH_Ca_), 2.59–2.50 (m, 1H, C*H*_cHex_, under DMSO peak), 2.41–2.36 (m, 2H, C*H*_2,ibu_), 2.26–1.94 (m, 3H, C*H*_cHex_), 1.83–1.74 (septet, J = 6.6 Hz, 1H, C*H*(CH_3_)_2_), 1.49–1.41 (m, 2H, C*H*_cHex_), 1.38–1.18 (m, 5H, C*H*_cHex_ + C*H*_3_CH), 1.15–1.06 (m, 1H, C*H*_cHex_), 1.00–0.88 (m, 1H, C*H*_cHex_), 0.87–0.81 (d, *J* = 6.5 Hz, 6H, C*H*_3,i-Pr_) ppm. ^13^C NMR (126 MHz, DMSO-*d*_6_) δ 181.5 (PtO*C*O), 170.9 (2x *C*O_Mal_), 163.3 (*C*O_Ox_), 163.2 (*C*O_Ox_), 163.1 (*C*O_Ca_), 139.3 (*C*_q,Ar_), 139.0 (*C*_q,Ar_), 134.6 (2x*C*H_Mal_), 128.8 (*C*H_Ar_), 128.7 (*C*H_Ar_), 127.2 (*C*H_Ar_), 127.1 (*C*H_Ar_), 69.8 (C*H*_PEG_), 69.7 (C*H*_PEG_), 69.6 (C*H*_PEG_), 69.5 (C*H*_PEG_), 69.4 (C*H*_PEG_), 69.2 (C*H*_PEG_), 66.9 (2xC*H*_PEG_), 60.9 (*C*H_cHex_), 60.7 (*C*H_cHex_), 46.1 (*C*HCH_3_), 44.3 (*C*H_2,Ibu_), 40.7 (*C*H_2_NH_Ca_), 36.8 (*C*H_2_N(CO)_2_), 31.0 (*C*H_cHex_), 30.8 (*C*H_cHex_), 29.6 (*C*H(CH_3_)_2_), 23.6 (*C*H_cHex_), 23.4 (*C*H_cHex_), 22.2 (CH(*C*H_3_)_2_), 19.1 (CH*C*H_3_) ppm. MS (*m*/*z*): calcd. C_36_H_54_N_4_O_14_NaPt (M + Na)^+^, 984.32; found, 984.33. EA calcd. C_36_H_54_N_4_O_14_Pt·0.5TFA; C, 43.61; H, 5.39; N, 5.5; found; C, 43.66; H, 5.37; N, 5.8.

##### 4,7-Dimethyl-2-(2-(2-(piperazin-1-yl)ethoxy)ethyl)-3a,4,7,7a-tetrahydro-1*H*-4,7-epoxyisoindole-1,3(2*H*)-dione (PMal-O-PIP-H)

PMal–O-Br (340 mg, 0.99 mmol) was dissolved in DMF (3 mL) and added dropwise over 1 h to a stirring solution of piperazine (426 mg, 4.95 mmol) and K_2_CO_3_ (687 mg, 4.95 mmol) in DMF (12 mL) at 50 °C with the aid of a syringe pump. After final addition, the reaction mixture was left stirring overnight at 50 °C, after which the solvent was removed under high vacuum (40 °C). The crude was partitioned between demi-H_2_O (15 mL) and DCM (3 × 30 mL), the total organic layer dried over Mg_2_SO_4_, vacuum filtrated and concentrated under reduced pressure to afford the title compound as a clear, yellow oil (206 mg, 60%).^1^H NMR (500 MHz, DMSO-*d*_6_) δ 6.37 (s, 2H, = C*H*_Furane_), 3.53–3.47 (t, *J* = 5.72 Hz, 2H, C*H*_2_N(CO)_2_), 3.46–3.40 (m, 4H, C*H*_PEG_), 2.89 (s, 2H, C*H*_Furane_), 2.66–2.59 (m, 4H, C*H*_2_N_pip_), 2.38–2.21 (m, 6H, C*H*_2_N_pip_), 1.53 (s, 6H, C*H*_3,Furane_) ppm. MS (*m*/*z*): calcd. C_18_H_27_N_3_O_4_ (M + H)^+^, 350.21; found, 350.24.

##### *tert*-Butyl-3-(4-(2-(2-(4,7-dimethyl-1,3-dioxo-1,3,3a,4,7,7a-hexahydro-2*H*-4,7-epoxyisoindol-2-yl)ethoxy)ethyl)piperazin-1-yl)propanoate (PMal-O-PIP-COOtBu)

PMal-O-PIP-H (205 mg, 0.59 mmol) was dissolved in CHCl_3_ (4 mL) and cooled to 0 °C in an ice-bath. *tert*-Butyl 3-bromopropanoate (148 μL, 0.71 mmol) and TEA (818 μL, 5.9 mmol) were added, the reaction mixture was left stirring for 2 h at 0 °C, then overnight at RT. Solvent was removed under reduced pressure, the crude partitioned between demi-H_2_O (10 mL) and DCM (3 × 20 mL), the total organic layer dried over Mg_2_SO_4_, vacuum filtrated and concentrated under reduced pressure. The crude was purified via silica flash column chromatography (6:94 MeOH:DCM) to afford to afford the title compound as a colorless wax (146 mg, 52%). ^1^H NMR (500 MHz, DMSO-*d*_6_) δ 6.37 (s, 2H, = C*H*_Furane_), 3.53–3.48 (t, *J* = 5.67 Hz, 2H, C*H*_2_N(CO)_2_), 3.46–3.41 (m, 4H, C*H*_PEG_), 2.89 (s, 2H, C*H*_Furane_), 2.51–2.45 (m, 2H, C*H*_2_COOtBu, under DMSO peak), 2.40–2.26 (m, 12H, C*H*_2_N_pip_), 1.53 (s, 6H, C*H*_3,Furane_), 1.39 (s, 9H, C*H*_3,tBu_) ppm. MS (*m*/*z*): calcd. C_25_H_39_N_3_O_6_ (M + H)^+^, 478.29; found, 478.34.

##### 3-(4-(2-(2-(4,7-Dimethyl-1,3-dioxo-1,3,3a,4,7,7a-hexahydro-2*H*-4,7-epoxyisoindol-2-yl)ethoxy)ethyl)piperazin-1-yl)propanoic Acid Dihydrochloride (PMal-O-PIP-COOH·2HCl)

PMal-O-PIP-COOtBu (145 mg, 0.3 mmol) was dissolved in 4 M HCl in dioxane (8 mL) and stirred overnight at RT. Solvent was removed under reduced pressure, crude was redissolved in demi-H_2_O (10 mL) and lyophilized to afforded the title compound as a light yellow crystalline solid (145 mg, 96%). ^1^H NMR (500 MHz, D_2_O) δ 6.45 (s, 2H, = C*H*_Furane_), 3.84–3.78 (m, 2H, C*H*_2_N(CO)_2_), 3.76–3.59 (m, 12H, C*H*_PEG_ + C*H*_2_N_Pip_), 3.48–3.38 (t, *J* = 6.79 Hz, 2H, C*H*_2_N_Pip_), 3.13 (s, 2H, C*H*_Furane_), 2.93–2.86 (t, *J* = 6.79 Hz, 2H, C*H*_2_COOH), 1.63 (s, 6H, C*H*_3,Furane_) ppm. MS (*m*/*z*): calcd. C_21_H_31_N_3_O_6_ (M + H)^+^, 422.17; found, 422.17.

##### 4-(((*S*)-1-(((*S*)-1-((4-(Hydroxymethyl)phenyl)amino)-1-oxo-5-ureidopentan-2-yl)amino)-3-methyl-1-oxobutan-2-yl)amino)-4-oxobutanoic Acid (HOOC-ValCitPAB–OH)

H_2_N-ValCitPAB–OH (100 mg, 0.254 mmol) and succinic anhydride (25.4 mg, 0.254 mmol) were dissolved in dry DMF (2 mL) under Argon at RT. TEA (38.9 μL, 0.28 mmol) was added and the reaction mixture was stirred for 1 h at RT, after which DMF was removed under high vacuum (40 °C). The crude was purified via preparative RP-HPLC (0–0.5 min A:B 95:5, 0.5–5.5 min linear gradient to A:B 45:55, 5.5–8.5 min A:B 5:95, 8.5–11.5 min A:B 95:5/A:H_2_O (+0.1% HCOOH), B:MeCN (+0.1% HCOOH)) and lyophilized to afford the title compound as a white solid (101 mg, 83%). ^1^H NMR (500 MHz, D_2_O) δ 7.42–7.35 (m, 4H, C*H*_Ar_), 4.59 (s, 2H, C*H*_2_OH), 4.41–4.36 (m, 1H, C*H*_α,Cit_), 4.12–4.07 (m, 1H, C*H*_α,Val_), 3.14–3.09 (t, *J* = 6,7 Hz, 2H, C*H*_δ,Cit_), 2.64–2.55 (m, 4H, C*H*_2,Succ_), 2.11–2.02 (m, 1H, C*H*_β,Val_), 1.95–1.76 (m, 2H, C*H*_γ,Cit_), 1.65–1.50 (m, 2H, C*H*_β,Cit_), 0.97–0.88 (m, 6H, C*H*_3,i-Pr_) ppm. MS (*m*/*z*): calcd. C_22_H_33_N_5_O_7_Na (M + Na)^+^, 493.25; found, 493.34.

##### *N*1-(2-(4-(2-(2-(4,7-Dimethyl-1,3-dioxo-1,3,3a,4,7,7a-hexahydro-2*H*-4,7-epoxyisoindol-2-yl)ethoxy)ethyl)piperazin-1-yl)ethyl)-*N*4-((*S*)-1-(((*S*)-1-((4-(hydroxymethyl)phenyl)amino)-1-oxo-5-ureidopentan-2-yl)amino)-3-methyl-1-oxobutan-2-yl)succinamide (PMal-O-PIP-Succ-ValCitPAB–OH)

HOOC-ValCitPAB–OH (24.6 mg, 0.0513 mmol) was dissolved in dry DMSO (0.3 mL) under argon at RT. TEA (10.8 μL, 0.077 mmol) and TBTU (21.5 mg, 0.067 mmol) were added and the reaction mixture was left stirring for 30 min. PMal-O-PIP-NH_2_ (34.2 mg, 0.087 mmol) was added as solution in dry DMSO (0.1 mL) and the resulting mixture was stirred overnight for 18 h. DMSO was removed under high vacuum (40 °C), the crude purified via preparative RP-HPLC (15% MeCN) (containing 0.1% HCOOH) in H_2_O (containing 0.1% HCOOH) and lyophilized to afford the title compound as a white solid (30.7 mg, 70%). ^1^H NMR (500 MHz, D_2_O) δ 7.46–7.36 (m, 4H, C*H*_Ar_), 6.43 (s, 2H, = C*H*_Furane,exo_), 6.30 (s, 2H, = C*H*_Furane,endo_), 4.59 (s, 2H, C*H*_2_OH), 4.43–4.37 (m, 1H, C*H*_α,Cit_), 4.11–4.07 (d, J = 6.6 Hz, 1H, C*H*_α,Val_), 3.71–3.64 (m, 4H, C*H*_PEG_ + C*H*_2_N(CO)_2_), 3.63–3.59 (m, 2H, C*H*_PEG_), 3.48 (s, 2H, C*H*_furane,endo_), 3.39–2.67 (m, 18H, C*H*_2_NH + C*H*_δ,Cit_ + C*H*_2_N_Pip_ + C*H*_Furane,exo_), 2.67–2.48 (m, 4H, C*H*_2,Succ_), 2.16–2.05 (m, 1H, C*H*_β,Val_), 1.98–1.89 (m, 1H, C*H*_γ,Cit_), 1.88–1.76 (m, 1H, C*H*_γ,Cit_), 1.73 (s, 2H, C*H*_3,Furane,endo_), 1.67–1.51 (m, 8H, C*H*_β,Cit_ + C*H*_3,Furane,exo_), 0.99–0.90 (m, *J* = 7.2 Hz, 6H, C*H*_3,i-Pr_) ppm. MS (*m*/*z*): calcd. C_42_H_63_N_9_O_10_ (M + H)^+^, 854.48; found, 854.54.

##### (2*S*)-2-((2*S*)-2-(3-(2-(4-(2-(2-(4,7-Dimethyl-1,3-dioxo-1,3,3a,4,7,7a-hexahydro-2*H*-4,7-epoxyisoindol-2-yl)ethoxy)ethyl)piperazin-1-yl)ethyl)thioureido)-3-methylbutanamido)-*N*-(4-(hydroxymethyl)phenyl)-5-ureidopentanamide (PMal-O-PIP-ThioU-ValCitPAB–OH)

PMal-O-PIP-NH_2_·3HCl (52.7 mg, 0.1 mmol) was dissolved in dry DMF (0.8 mL) under argon at RT. Triethylamine (69.7 μL, 0.65 mmol) was added and the mixture was stirred for 5 min at RT, after which the precipitate was filtered off. TCDI (21.4 mg, 0.12 mmol) was added to the filtrate and the reaction mixture was left stirring for 3 h. H_2_N-ValCitPAB–OH (49.3 mg, 0.13 mmol) and TEA (27.9 μL, 0.2 mmol) were added and the total reaction mixture was left stirring overnight for 18 h. DMF was removed under high vacuum, the crude was purified via preparative RP-HPLC (0–0.5 min A:B 95:5, 0.5–15 min linear gradient to A:B 58:42, 15–18 min A:B 5:95, 18–21 min A:B 95:5/A:H_2_O (containing 0.1% HCOOH), B:MeCN (containing 0.1% HCOOH)) and lyophilized to afford the title compound as a white solid (52.4 mg, 64%). ^1^H NMR (500 MHz, D_2_O) δ 7.45–7.35 (m, 4H, C*H*_Ar_), 6.43 (s, 2H, = C*H*_Furane,exo_), 6.30 (s, 2H, = C*H*_Furane,endo_), 4.59 (s, 2H, C*H*_2_OH), 4.45–4.39 (m, 2H, C*H*_α,Val_ + C*H*_α,Cit_), 3.94–3.53 (m, 8H, C*H*_PEG_ + C*H*_2_N(CO)_2_ + C*H*_2_NHS), 3.46 (s, 2H, C*H*_furane,endo_) 3.42–2.34 (m, 16H, C*H*_δ,Cit_ + C*H*_2_N_Pip_ + C*H*_Furane,exo_), 2.19–2.07 (m, 1H, C*H*_β,Val_), 1.98–1.89 (m, 1H, C*H*_γ,Cit_), 1.87–1.75 (m, 1H, C*H*_γ,Cit_), 1.72 (s, 2H, C*H*_3,Furane,endo_), 1.69–1.50 (m, 8H, C*H*_β,Cit_ + C*H*_3,Furane,exo_), 1.01–0.90 (m, 6H, C*H*_3,i-Pr_) ppm. MS (*m*/*z*): calcd. C_39_H_59_N_9_O_8_S (M + H)^+^, 814.43; found, 814.49.

##### (2*S*)-2-((2*S*)-2-(3-(4-(2-(2-(4,7-Dimethyl-1,3-dioxo-1,3,3a,4,7,7a-hexahydro-2*H*-4,7-epoxyisoindol-2-yl)ethoxy)ethyl)piperazin-1-yl)propanamido)-3-methylbutanamido)-*N*-(4-(hydroxymethyl)phenyl)-5-ureidopentanamide (PMal-O-PIP-ValCitPAB–OH)

PMal-O-PIP-COOH (50.9 mg, 0.103 mmol) was dissolved in dry DMF (0.4 mL) under argon at RT. TEA (86.1 μL, 0.618 mmol) was added and the mixture was left stirring for 10 min at RT, after which the precipitate was filtered off and washed with dry DMF (0.2 mL). EDC·HCl (21.7 mg, 0.113 mmol), HOBt (13.9 mg, 0.103 mmol) and H_2_N-ValCitPAB–OH (19.5 mg, 0.052 mmol) were added and the total reaction mixture was left stirring for another 24 h at RT. DMF was removed under high vacuum (RT), the crude purified via preparative RP HPLC (0–0.5 min A:B 95:5, 0.5–15 min linear gradient to A:B 60:40, 15–18 min A:B 5:95, 18–21 min A:B 95:5/A:H_2_O (+0.1% HCOOH), B:MeCN (+0.1% HCOOH)) and lyophilized to afford the title compound (21 mg, 51%) as an off-white crystalline solid. ^1^H NMR (500 MHz, D_2_O) δ 7.43–7.32 (m, 4H, C*H*_Ar_), 6.43 (s, 2H, = C*H*_Furane_), 4.59 (s, 2H, C*H*_2_OH), 4.43–4.37 (m, 1H, C*H*_α,Cit_), 4.14–4.08 (d, J = 7.2 Hz, 1H, C*H*_α,Val_), 3.72–3.56 (m, 6H, C*H*_PEG_ + C*H*_2_N(CO)_2_), 3.47–2.29 (m, 18H, C*H*_furane_ + C*H*_2_NH + C*H*_δ,Cit_ + C*H*_2_N_Pip_), 2.11–2.01 (m, 1H, C*H*_β,Val_), 1.94–1.75 (m, 2H, C*H*_γ,Cit_), 1.67–1.49 (m, 8H, C*H*_3,Furane_ + C*H*_β,Cit_), 0.98–0.89 (d, J = 6.7 Hz, 6H, C*H*_3,i-Pr_) ppm. MS (*m*/*z*): calcd. C_39_H_59_N_8_O_9_ (M + H)^+^, 783.44; found, 783.42.

##### *N*1-(2-(4-(2-(2-(2,5-Dioxo-2,5-dihydro-1*H*-pyrrol-1-yl)ethoxy)ethyl)piperazin-1-yl)ethyl)-*N*4-((*S*)-1-(((*S*)-1-((4-(hydroxymethyl)phenyl)amino)-1-oxo-5-ureidopentan-2-yl)amino)-3-methyl-1-oxobutan-2-yl)succinamide (Mal-O-PIP-Succ-ValCitPAB–OH)

Synthesized according to general procedure K based on PMal-O-PIP-Succ-ValCitPAB–OH (26.2 mg, 0.03 mmol) with 2 h of stirring. Crude purified via preparative RP HPLC (0–0.5 min A:B 95:5, 0.5–15 min linear gradient to A:B 50:50, 15–18 min A:B, 18–21 min A:B 95:5/A:H_2_O (containing 0.1% HCOOH), B:MeCN (containing 0.1% HCOOH)) and lyophilized to afford the title compound as a white solid (14.5 mg, 64%). ^1^H NMR (500 MHz, D_2_O) δ 7.46–7.35 (m, 4H, C*H*_Ar_), 6.83 (s, 2H, C*H*_Mal_), 4.59 (s, 2H, C*H*_2_OH), 4.43–4.37 (m, 1H, C*H*_α,Cit_), 4.11–4.07 (d, J = 6.8 Hz, 1H, C*H*_α,Val_), 3.79–3.56 (m, 6H, C*H*_2_N(CO)_2_ + C*H*_PEG_), 3.39–2.29 (m, 20H, C*H*_2_NH + C*H*_δ,Cit_ + C*H*_2_N_Pip_ + CH_2,Succ_), 2.16–2.05 (m, 1H, C*H*_β,Val_), 1.98–1.89 (m, 1H, C*H*_γ,Cit_), 1.88–1.76 (m, 1H, C*H*_γ,Cit_), 1,68–1.48 (m, 2H, C*H*_β,Cit_), 0.99–0.90 (m, 6H, C*H*_3,i-Pr_) ppm. ^13^C NMR (126 MHz, D_2_O) δ 175.7 (2x*C*ONH), 174.0 (2x*C*ONH), 173.0 (2x*C*O_Mal_), 172.2 (*C*ONH), 161.4 (*C*ONH_2_), 137.7 (*C*_q,Ar_), 135.8 (*C*_q,Ar_), 134.4 (2x*C*H_Mal_), 128.3 (2x*C*H_Ar_), 121.9 (2x*C*H_Ar_), 68.0 (2x*C*H_PEG_), 63.4 (*C*H_2_OH), 59.9 (C*H*_α,Val_), 55.8 (3xC*H*_2_N_Pip_), 55.4 (3xC*H*_2_N_Pip_), 54.2 (C*H*_α,Cit_), 39.2 (C*H*_δ,Cit_), 37.0 (*C*H_2_N(CO)_2_), 35.3 (*C*H_2_NHCO), 30.5 (*C*H_2,Succ_), 30.2 (*C*H_2,Succ_), 29.8 (C*H*_β,Val_), 28.1 (C*H*_β,Cit_), 25.8 (C*H*_γ,Cit_), 18.3 (C*H*_3,i-Pr_), 17.4 (C*H*_3,i-Pr_) ppm. MS (*m*/*z*): calcd. C_36_H_55_N_9_O_9_ (M + H)^+^, 758.42; found, 758.45.

##### (*S*)-2-((*S*)-2-(3-(2-(4-(2-(2-(2,5-Dioxo-2,5-dihydro-1*H*-pyrrol-1-yl)ethoxy)ethyl)piperazin-1-yl)ethyl)thioureido)-3-methylbutanamido)-*N*-(4-(hydroxymethyl)phenyl)-5-ureidopentanamide (Mal-O-PIP-ThioU-ValCitPAB–OH)

Synthesized according to general procedure K based on PMal-O-PIP-ThioU-ValCitPAB–OH (25 mg, 0.03 mmol) with 1 h of stirring. Crude purified via preparative RP-HPLC (0–0.5 min A:B 95:5, 0.5–15 min linear gradient to A:B 62:38, 15–18 min A:B, 18–21 min A:B 95:5/A:H_2_O (containing 0.1% HCOOH), B:MeCN (containing 0.1% HCOOH)) and lyophilized to afford the title compound as a white solid (11.2 mg, 51%). ^1^H NMR (500 MHz, D_2_O) δ 7.48–7.38 (m, 4H, C*H*_Ar_), 6.87 (s, 2H, C*H*_Mal_), 4.62 (s, 2H, C*H*_2_OH), 4.59–4.45 (m, 2H, C*H*_α,Cit_ + C*H*_α,Val_), 3.94–3.58 (m, 8H, C*H*_PEG_ + C*H*_2_N(CO)_2_ + C*H*_2_NHS), 3.70–3.63 (m, 2H, C*H*_PEG_), 3.40–2.64 (m, 14H, C*H*_δ,Cit_ + C*H*_2_N_Pip_), 2.21–2.13 (m, 1H, C*H*_β,Val_), 2.01–1.93 (m, 2H, C*H*_γ,Cit_), 1.88–1.80 (m, 2H, C*H*_γ,Cit_), 1H, 1.71–1.54 (m, 2H, C*H*_β,Cit_), 1.04–0.97 (d, J = 6.7 Hz, 6H, C*H*_3,i-Pr_) ppm. ^13^C NMR (126 MHz, D_2_O) δ 173.0 (2x*C*O_Mal_ + CS), 172.2 (2x*C*ONH), 161.4 (*C*ONH_2_), 137.7 (*C*_q,Ar_), 135.7 (*C*_q,Ar_), 134.4 (2x*C*H_Mal_), 128.3 (2x*C*H_Ar_), 122.3 (2x*C*H_Ar_), 68.0 (2x*C*H_PEG_), 64.5 (C*H*_α,Val_), 63.4 (*C*H_2_OH), 55.8 (3xC*H*_2_N_Pip_), 55.6 (3xC*H*_2_N_Pip_), 54.0 (C*H*_α,Cit_), 40.1 (C*H*_δ,Cit_), 37.0 (*C*H_2_N(CO)_2_ + *C*H_2_NHS), 30.4 (C*H*_β,Val_), 28.1 (C*H*_β,Cit_), 25.8 (C*H*_γ,Cit_), 18.5 (C*H*_3,i-Pr_), 17.7 (C*H*_3,i-Pr_) ppm. MS (*m*/*z*): calcd. C_33_H_51_N_9_O_7_S (M + H)^+^, 718.37; found, 718.41.

##### (*S*)-2-((*S*)-2-(3-(4-(2-(2-(2,5-Dioxo-2,5-dihydro-1*H*-pyrrol-1-yl)ethoxy)ethyl)piperazin-1-yl)propanamido)-3-methylbutanamido)-*N*-(4-(hydroxymethyl)phenyl)-5-ureidopentanamide (Mal-O-PIP-ValCitPAB–OH)

Synthesized according to general procedure K based on PMal-O-PIP-ValCitPAB–OH (16 mg, 0.02 mmol) with 2 h of stirring. Crude purified via preparative RP HPLC (0–0.5 min A:B 95:5, 0.5–15 min linear gradient to A:B 64:36, 15–18 min A:B, 18–21 min A:B 95:5/A:H_2_O (+0.1% HCOOH), B:MeCN (+0.1% HCOOH)) and lyophilized to afford the title compound as a white solid (9.1 mg, 65%). ^1^H NMR (500 MHz, D_2_O) δ 7.44–7.32 (m, 4H, C*H*_Ar_), 6.83 (s, 2H, C*H*_Mal_), 4.58 (s, 2H, C*H*_2_OH), 4.43–4.36 (m, 1H, C*H*_α,Cit_), 4.14–4.09 (d, *J* = 7.3 Hz, 1H, C*H*_α,Val_), 3.73–3.59 (m, 6H, C*H*_PEG_ + C*H*_2_N(CO)_2_), 3.20–2.39 (m, 16H, C*H*_δ,Cit_ + C*H*_2_N_Pip_ + *C*H_2_CO), 2.11–2.01 (m, 1H, C*H*_β,Val_), 1.93–1.75 (m, 2H, C*H*_γ,Cit_), 1.66–1.48 (m, 2H, C*H*_β,Cit_), 1.03–0.92 (d, *J* = 6.8 Hz, 6H, C*H*_3,i-Pr_) ppm. ^13^C NMR (126 MHz, D_2_O) δ 173.7 (2x*C*ONH), 173.0 (2x*C*O_Mal_), 172.2 (2x*C*ONH), 161.4 (*C*ONH_2_), 137.7 (*C*_q,Ar_), 135.8 (*C*_q,Ar_), 134.6 (2x*C*H_Mal_), 128.3 (2x*C*H_Ar_), 122.1 (2x*C*H_Ar_), 68.0 (2x*C*H_PEG_), 63.4 (*C*H_2_OH), 59.6 (C*H*_α,Val_), 55.8 (3xC*H*_2_N_Pip_), 54.2 (C*H*_α,Cit_), 52.4 (3xC*H*_2_N_Pip_), 38.9 (C*H*_δ,Cit_), 37.0 (*C*H_2_N(CO)_2_ + *C*H_2_CO), 30.1 (C*H*_β,Val_), 28.2 (C*H*_β,Cit_), 25.7 (C*H*_γ,Cit_), 18.4 (C*H*_3,i-Pr_), 17.7 (C*H*_3,i-Pr_) ppm. MS (*m*/*z*): calcd. C_33_H_50_N_8_O_8_ (M + H)^+^, 687.38; found, 687.25.

#### Hydrolytic Stability Experiments

Phosphate buffer (150 mM, pH 7.4) containing 1 mM platinum compound was incubated at 20 °C. The reaction was monitored on a Thermo Scientific Dionex UltiMate 3000 UHPLC-system using a Waters Acquity UPLC BEH C18 1.7 μm 3.0 mm × 50 mm column. Milli-Q water, containing 0.1% formic acid, and acetonitrile containing 0.1% formic acid were used as eluents. A gradient of 5–95% over 5 min was used. The peak area of the parental complex was used to evaluate the current state of the reaction. This was done due to the fact, that in most cases the hydrolysis products did not have a sufficient retention time to be quantified.

### SEC-ICP-MS Measurements

FCS was purchased from Sigma-Aldrich and buffered with 150 mM phosphate pH 7.4 in order to guarantee a stable pH. The platinum(IV) complexes (5 mM) were dissolved in 150 mM phosphate buffer (pH 7.4) and diluted 1:100 in the buffered serum to obtain a final concentration of 100 μM. The samples were incubated in the autosampler at 37 °C and analyzed at 0, 1, 4, 24, and 25 h time points. Between each sample a pure water blank was measured. For SEC-ICP-MS measurements an Agilent 1260 Infinity system coupled to an Agilent 7800 ICP-MS equipped with a dynamic reaction cell was used. Oxygen (purity 5.5, Messer Austria GmbH, Gumpoldskirchen, Austria) was used as reaction gas. HPLC parameters are given in Table S2 and ICP-MS operation parameters are given in Table S3.

### *In Vivo* Experiments

#### Cells

For *in vivo* experiments the murine (Balb/c) CT-26 (colon cancer) tumor model was used, which were cultured in DMEM/F-12 (1:1) medium (purchased from ATCC). The media were supplemented with 10% FCS (purchased from PAA Linz, Austria). All cells were not treated with antibiotics and were regularly checked for mycoplasma contamination. All cell culture media and reagents were purchased from Sigma-Aldrich Austria.

#### Animals

Eight- to 12-week-old Balb/c mice were purchased from Envigo Laboratories (San Pietro al Natisone, Italy). The animals were kept in a pathogen-free conditions, controlled environment with 12 h light–dark cycle and every procedure was done in a laminar airflow cabinet. All experiments were approved by the Ethics Committee for the Care and Use of Laboratory Animals at the Medical University Vienna (BMWF-2022–0.770.291) and performed according to the guidelines from the Austrian Animal Science Association and from the Federation of European Laboratory Animal Science Associations (FELASA).

#### Serum PK and Tumor/Organ Distribution

CT-26 cells (5 × 10^5^ in 50 μL serum-free medium) were injected subcutaneously (s.c.) into the right flank of male Balb/c mice. After 10 days of cell injection, the drugs were applied i.v. (*n* = 4 per treatment group) with a single dose equimolar to 9 mg/kg oxaliplatin dissolved in 0.9% NaCl. For serum PK, blood was collected after 5, 30 min, 5, and 24 h (2 mice per time point) via the facial vein. The serum was separated by centrifugation (2 times 10 min at 900 g) after 10 min clotting at RT. The tumor and organ accumulation was determined after 5 and 24 h (2 animals per time point). To this end, the animals were sacrificed via cervical dislocation and the tumor was harvested. All collected samples were stored at −20 °C and further processed for platinum measurements via ICP-MS. For these measurements, HNO_3_ (67–69%, suprapur for trace metal analysis, NORMATOM; Distributor: VWR international, Austria) and conc. H_2_O_2_ suprapur (Merck, Wasserstoffperoxid 30%) were used without further purification. Digestion of tissue (approximately 15–30 mg gravimetrically weighted) was performed with 2 mL of approximately 20% nitric acid and 100 μL H_2_O_2_ using an open vessel graphite digestion system (coated graphite heating plate, coated sample holder-top for 25 mL vials, PFA vials and PFA lids; Labter, ODLAB; Distributor: AHF Analysentechnik AG; Germany). Digested samples were diluted in Milli-Q water (18.2 MΩ·cm, Milli-Q Advantage, Darmstadt, Germany). The platinum concentration was determined by ICP-MS analysis. Therefore, platinum and rhenium standards were derived from CPI International (Amsterdam, The Netherlands). The total platinum content was determined with a quadrupole-based ICP-MS instrument Agilent 7800 (Agilent Technologies, Tokyo, Japan) equipped with the Agilent SPS 4 autosampler (Agilent Technologies, Tokyo, Japan) and a MicroMist nebulizer at a sample uptake rate of approximately 0.2 mL/min. An RF power of 1550 W was used as well as nickel cones. Argon was used as plasma gas (15 L/min) and as carrier gas (∼1.1 L/min). The dwell time was set to 0.1 s and the measurements were performed in 6 replicates with 100 sweeps. Rhenium served as internal standard for platinum. The Agilent MassHunter software package (Workstation Software, version C.01.04) was used for data processing.

#### Aqueous Solubility Measurements

5% glucose and 0.9% NaCl solutions were purchased from B. Braun. 0.9% NaCl solutions were acidified to pH 3.0 using 1 M HCl and basified to pH 7.0 using 1 M NaOH. Aqueous buffers (citrate, PB, PBS) were prepared in 100 mM concentrations. One mg of compound was weighted using a Sartorius ME215P analytical balance and the solubility was investigated according to the following stepwise interval: +1 μL in the volume range 0–10 μL; +10 μL in the volume range 10–100 μL; +100 μL in the volume range 100–1000 μL; +1000 μL in the volume range 1000–5000 μL. Dissolution of the sample was investigated using a Leica M80 routine stereo microscope (50× magnification) until no solid particles were observed.

## Supporting Information Available

Sulfur traces of FCS, organ distribution (lung, spleen, brain), aqueous solubility of Mal-ValCit linkers, SEC-ICP-MS operation parameters, ^1^H NMR spectra of all novel compounds and additional ^13^C NMR spectra of PMal-X-PIP ligands (X = O, C_5_), final platinum(IV) complexes and final Mal-ValCit linkers (PDF)
