# Supplementary material for: Novel Maleimide Linkers Based on a Piperazine Motif for Strongly Increased Aqueous Solubility
Source: ACS Omega. 2025 Jan 31;10(5):5047–63. doi: 10.1021/acsomega.4c10825 (PMC11822723; doi:10.1021/acsomega.4c10825)
Supplement: Supplementary file 1 — ao4c10825_si_001.pdf [file ao4c10825_si_001.pdf]

Novel maleimide linkers based on a piperazine motif for strongly increased aqueous solubility

Martijn Dijkstra,[a,b] Hemma Schueffl,[c] Anja Federa,[a,b] Caroline Kast,[a] Alexander Unterlercher,[a] Bernhard K. Keppler,[a,d] Petra Heffeter,[c,d] and Christian R. Kowol*[a,d]

a) University of Vienna, Faculty of Chemistry, Institute of Inorganic Chemistry, Waehringer Str. 42, 1090 Vienna, Austria. E-mail: christian.kowol@univie.ac.at.

d) University of Vienna, Vienna Doctoral School in Chemistry (DoSChem), Waehringer Str. 42, 1090 Vienna, Austria.

c) Center for Cancer Research and Comprehensive Cancer Center, Medical University of Vienna, Borschkegasse 8a, 1090 Vienna, Austria.

d) Research Cluster "Translational Cancer Therapy Research", 1090 Vienna, Austria.

Table of contents

Figure/table	Content	Page number
Figure S1	Sulfur traces of fetal calf serum	2
Figure S2	Organ distribution (lung, spleen, brain)	2
Table S1	Aqueous solubility of Mal-PIP-ValCitPAB-OH derivatives	3
Table S2-3	SEC-ICP-MS operation parameters	3
Figures S3-37	^{1}H NMR spectra of all novel compounds + additional ^{13}C NMR spectra of PMal-X-PIP ligands (X= O, C_5) , final platinum(IV) complexes and final Mal-ValCit linkers	4–30

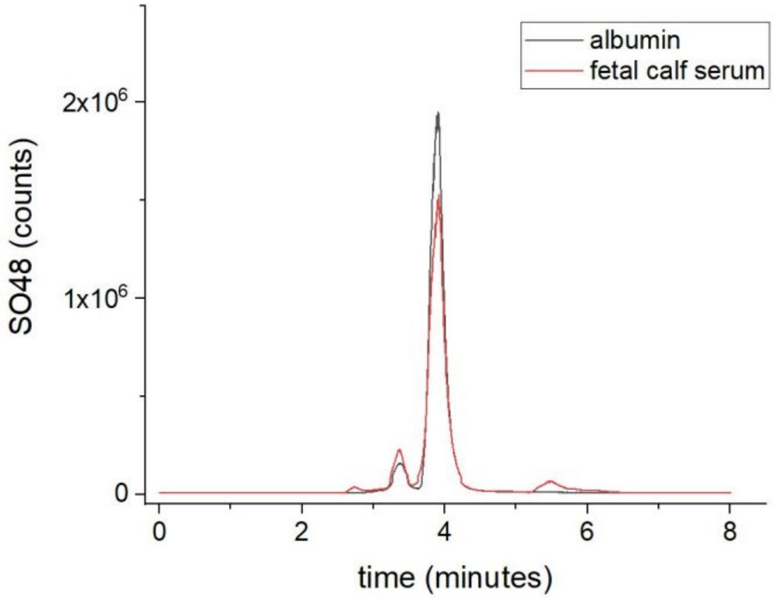

Figure S1. Sulfur traces of fetal calf serum (+150 mM phosphate buffer, pH 7.4) as well as pure albumin in phosphate buffer (50 mM, pH 7.4), both measured by SEC-ICP-MS. The small peak at ~3.3 min corresponds to the albumin dimer.

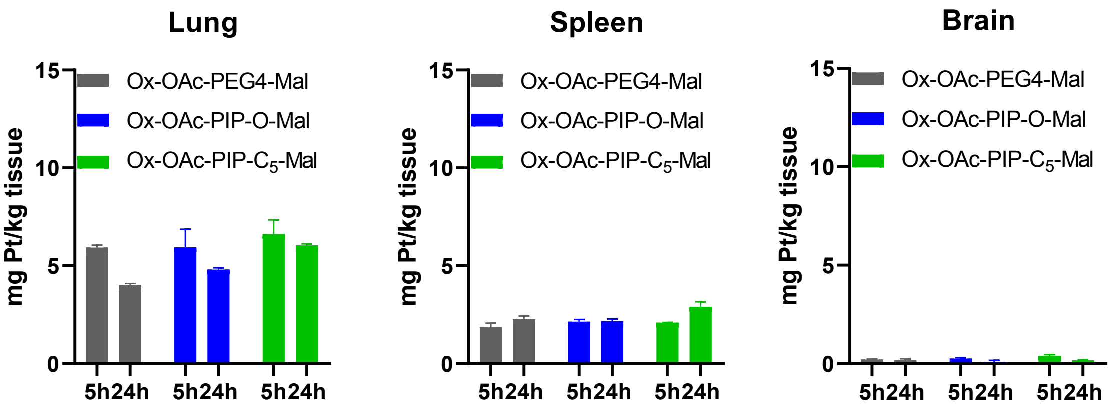

Figure S2. Organ distribution of the tested drugs**.** Animals were treated once i.v. with concentrations equimolar to 9 mg/kg oxaliplatin. Organ samples were collected after 5 h and 24 h. Platinum levels of all samples were measured via ICP-MS.

Table S1. Aqueous solubility of Mal-PIP-ValCitPAB-OH derivatives

Entry	Medium	**Mal-PEG4-ValCitPAB-OH**	**Mal-O-PIP-ValCitPAB-OH**		**Mal-O-PIP-Succ-ValCitPAB-OH**		**Mal-O-PIP-ThioU-ValCitPAB-OH**	
		Conc. (mM)	Conc. (mM)	Factor*	Conc. (mM)	Factor*	Conc. (mM)	Factor*
1	5% glucose	20	700	30	600	30	600	30
2	0.9% NaCl	40	1500	40	1300	40	1400	40

*Factors were calculated *via* dividing the measured concentrations of the respective **Mal-X-PIP-ValcitPAB-OH** derivative and that of **Mal-PEG4-ValCitPAB-OH** in the same solvent. All solubilities were tested at least in duplicate.

Table S2. HPLC operation parameters used in SEC-ICP-MS measurements

Samples	100 µM in FCS (150 mM PB, pH 7.4)
Column	Acquity UPLC BEH 200Å 1.7 µm, 4.6x150 mm
Eluent	50 mM CH_3COONH_4, pH = 6.8
Flow rate	400 µl/min
Column temperature	37°C
Sample temperature	37°C
Injection volume	0.5 µl

Table S3. Flow injection parameters used in SEC-ICP-MS measurements

Samples	50 µM in FCS (150 mM PB, pH 7.4)
Column	n.a.
Eluent	50 mM CH_3COONH_4, pH = 6.8
Flow rate	400 µl/min
Column temperature	37°C
Sample temperature	20°C
Injection volume	2 µl

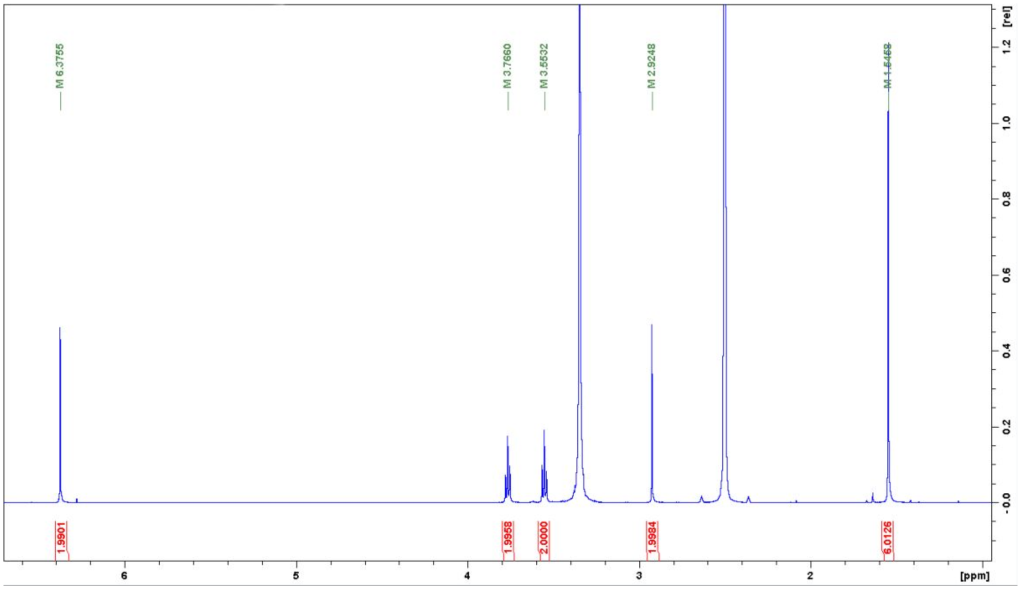

Figure S3. ^{1}H NMR spectrum of **PMal-O-Br**, measured in DMSO-d6.

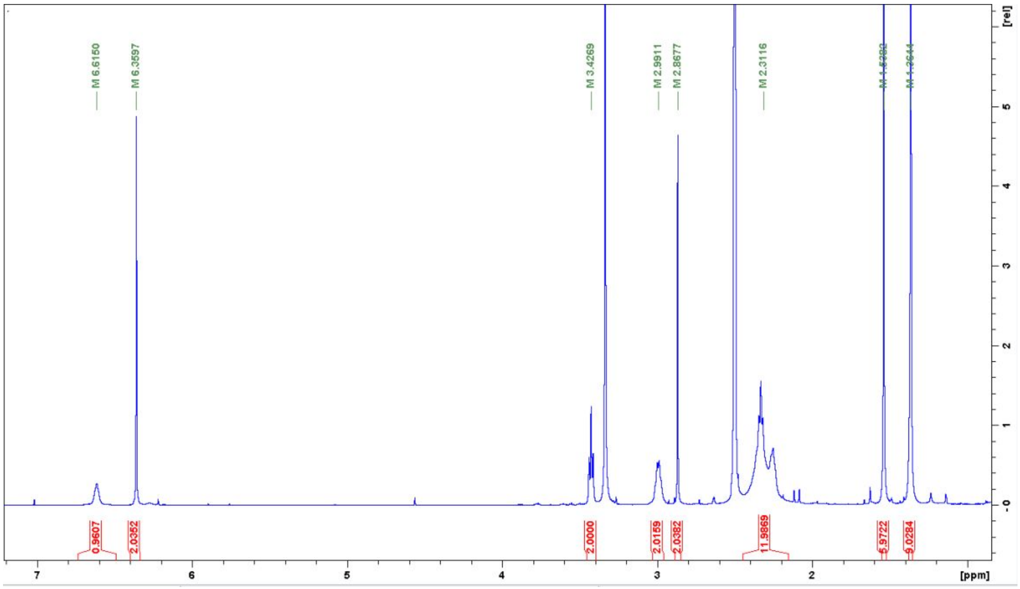

Figure S4. ^{1}H NMR spectrum of **PMal-C$_2$-PIP-NHBoc**, measured in DMSO-d6.

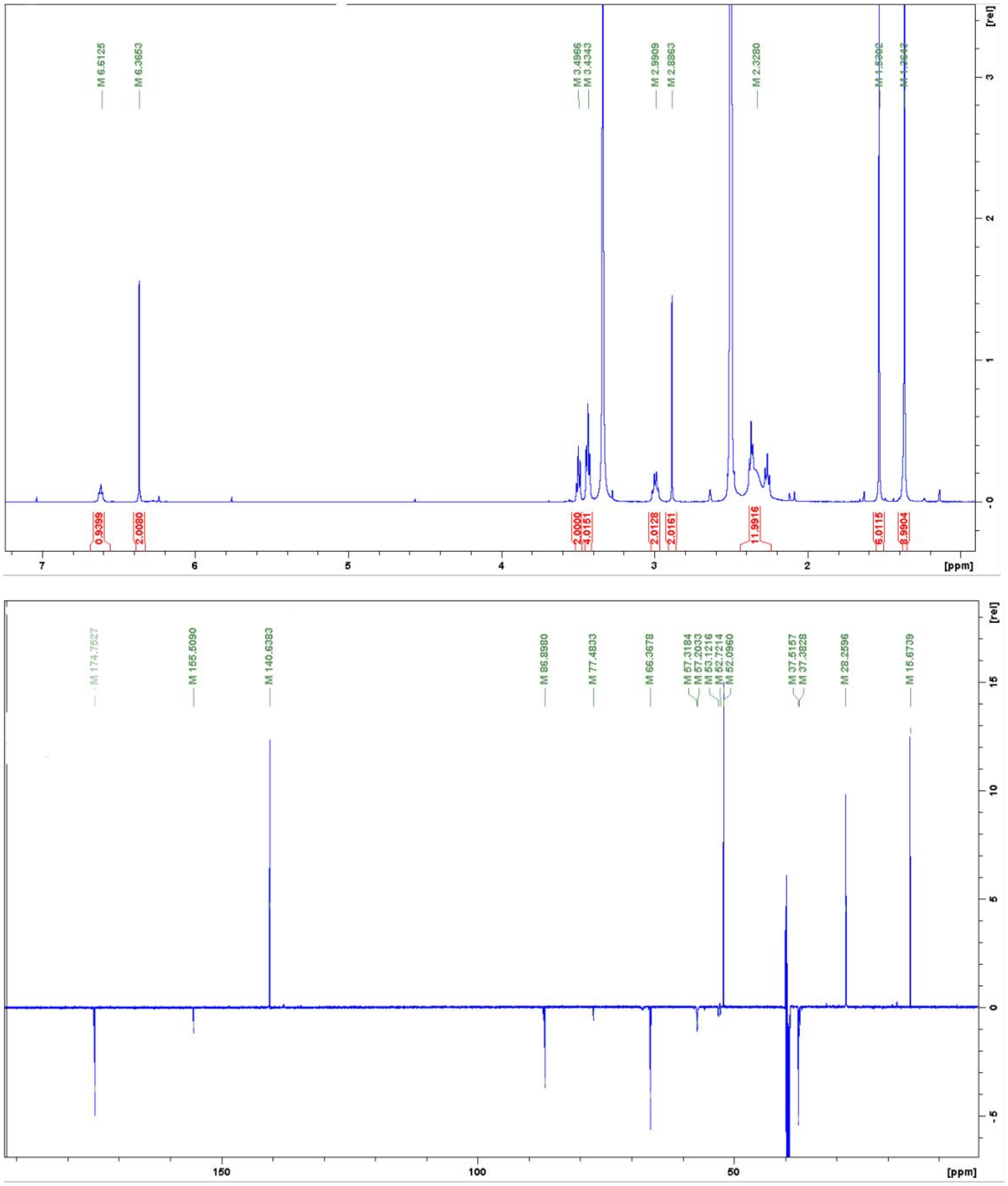

Figure S5. ^{1}H and ^{13}C NMR spectra of **PMal-O-PIP-NHBoc**, measured in DMSO-d6.

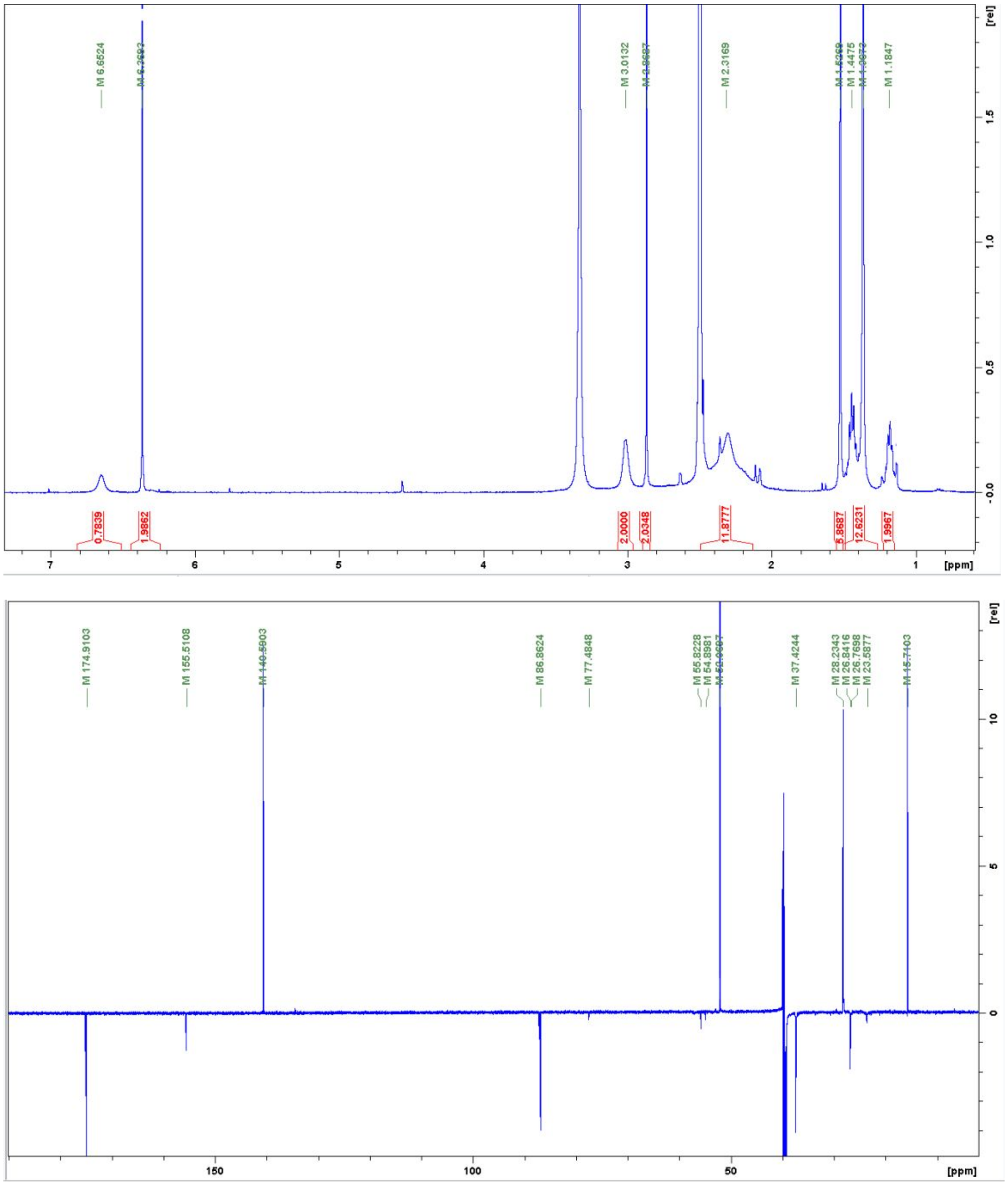

Figure S6. ^{1}H and ^{13}C NMR spectra of **PMal-C$_5$-PIP-NHBoc**, measured in DMSO-d6.

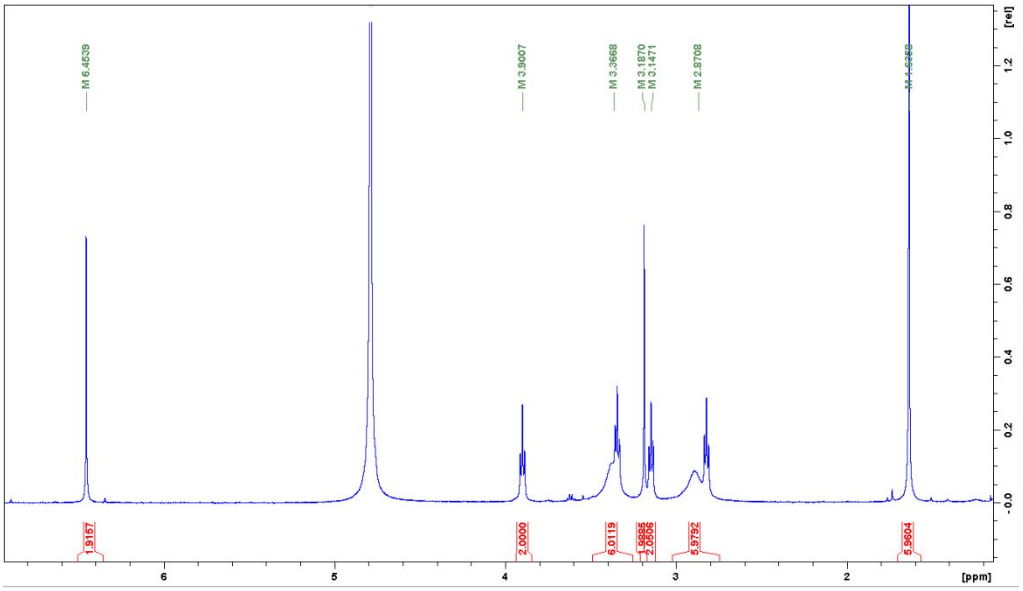

Figure S7. ^{1}H NMR spectrum of **PMal-C$_2$-PIP-NH$_2$·3HCl**, measured in D_2O.

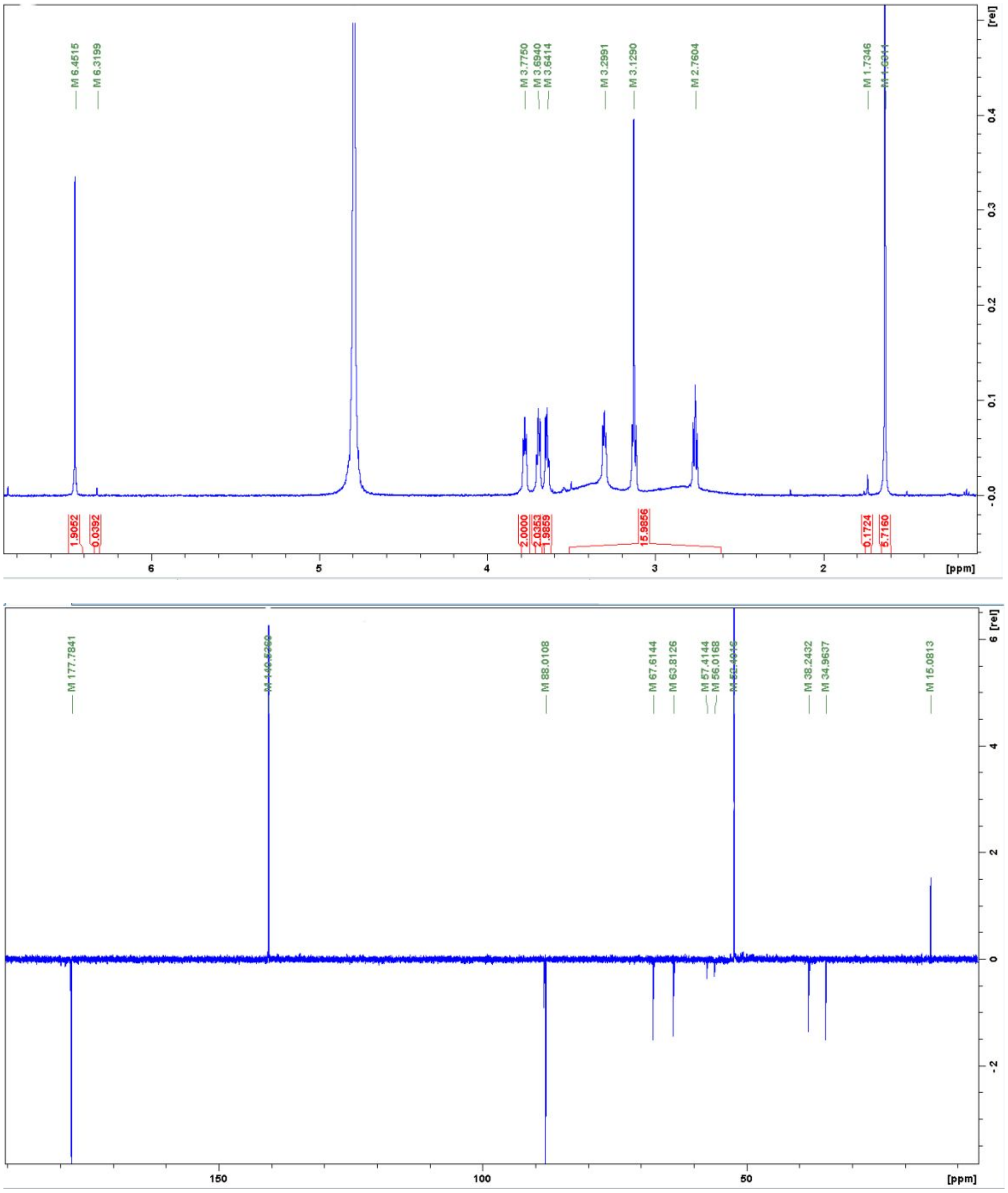

Figure S8. ^{1}H and ^{13}C NMR spectra of **PMal-O-PIP-NH$_2$·3HCl**, measured in D_2O

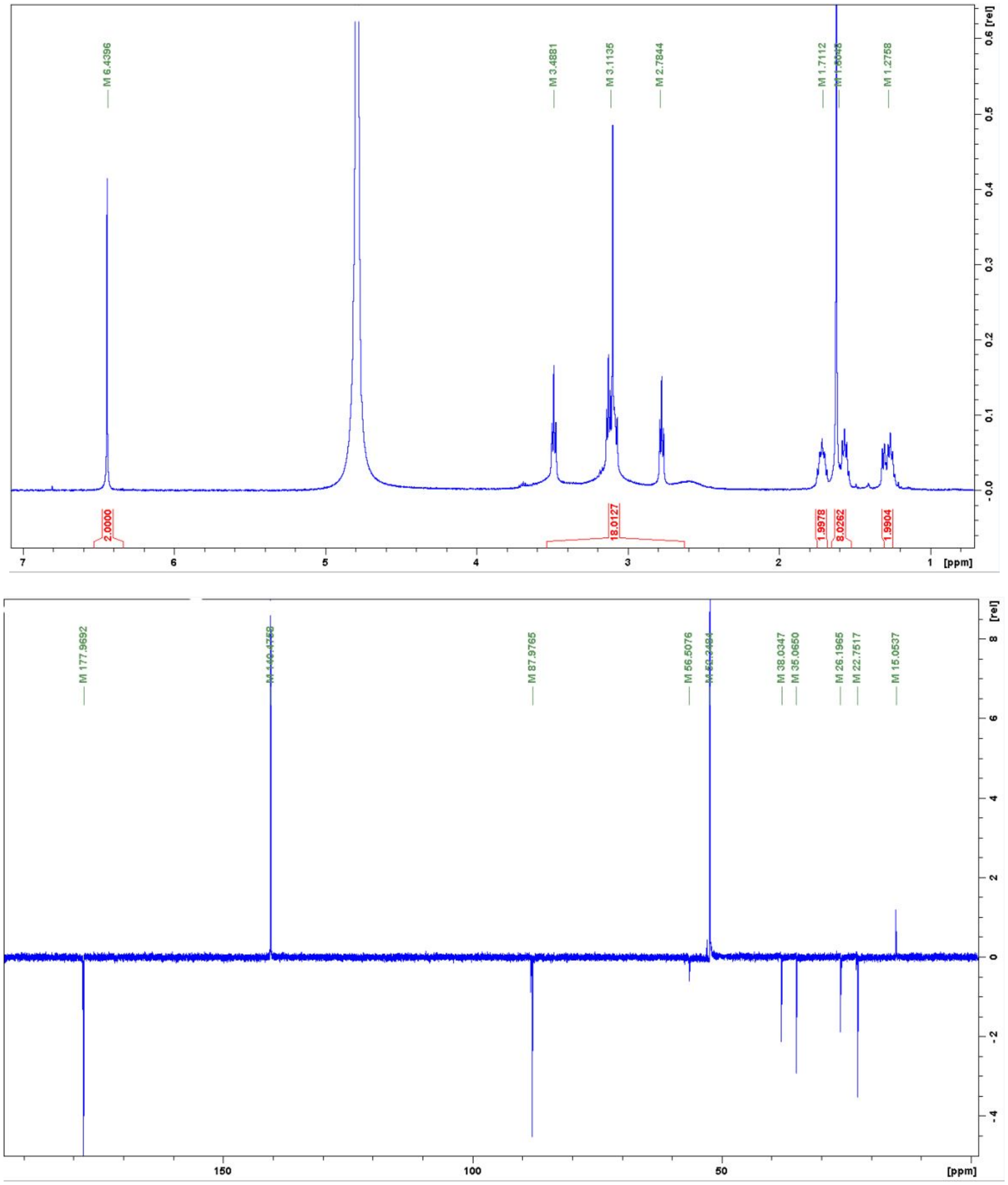

Figure S9. ^{1}H and ^{13}C NMR spectra of **PMal-C$_5$-PIP-NH$_2$·3HCl**, measured in D_2O.

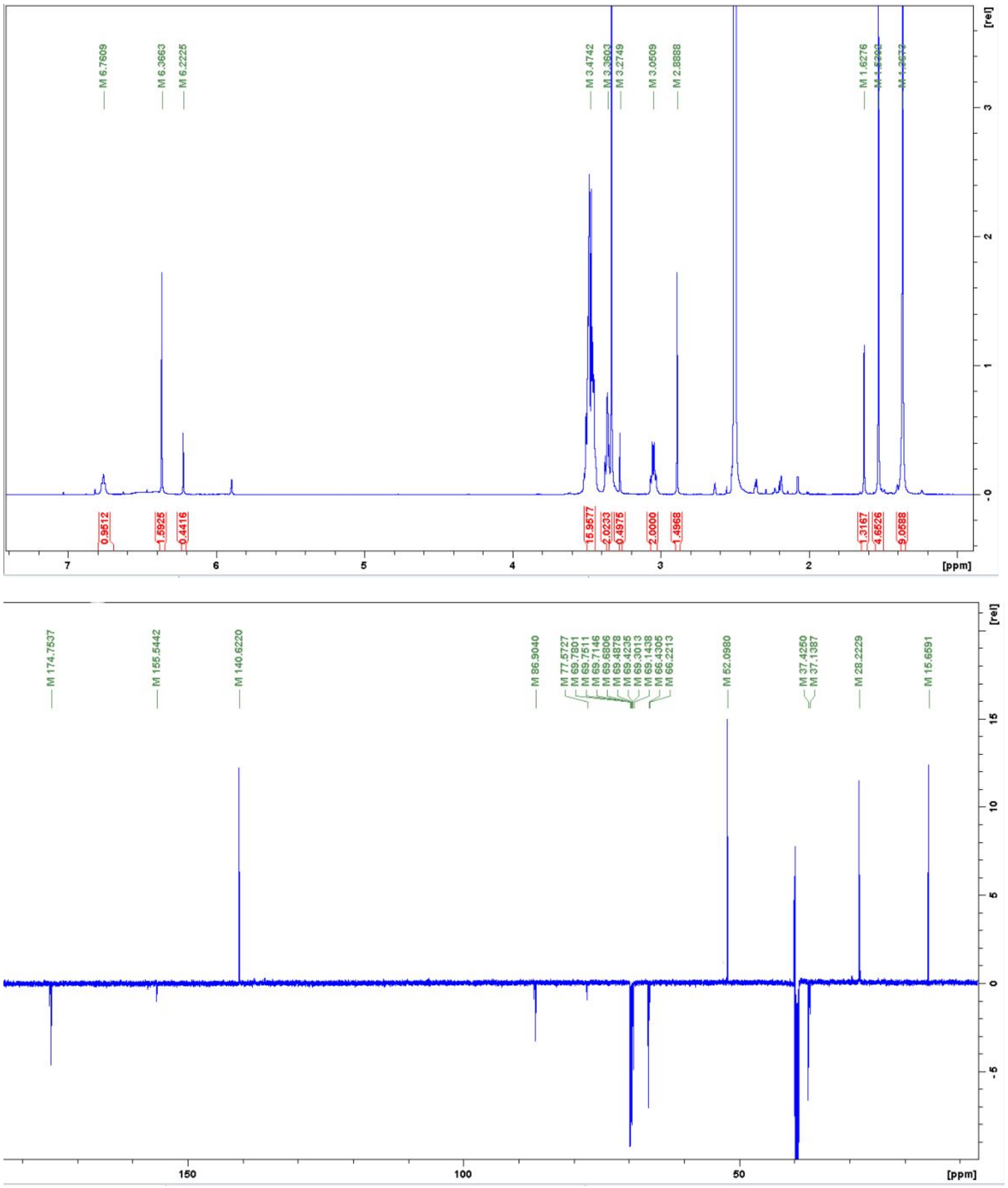

Figure S10. ^{1}H and ^{13}C NMR spectra of **PMal-PEG4-NHBoc**, measured in DMSO-d6.

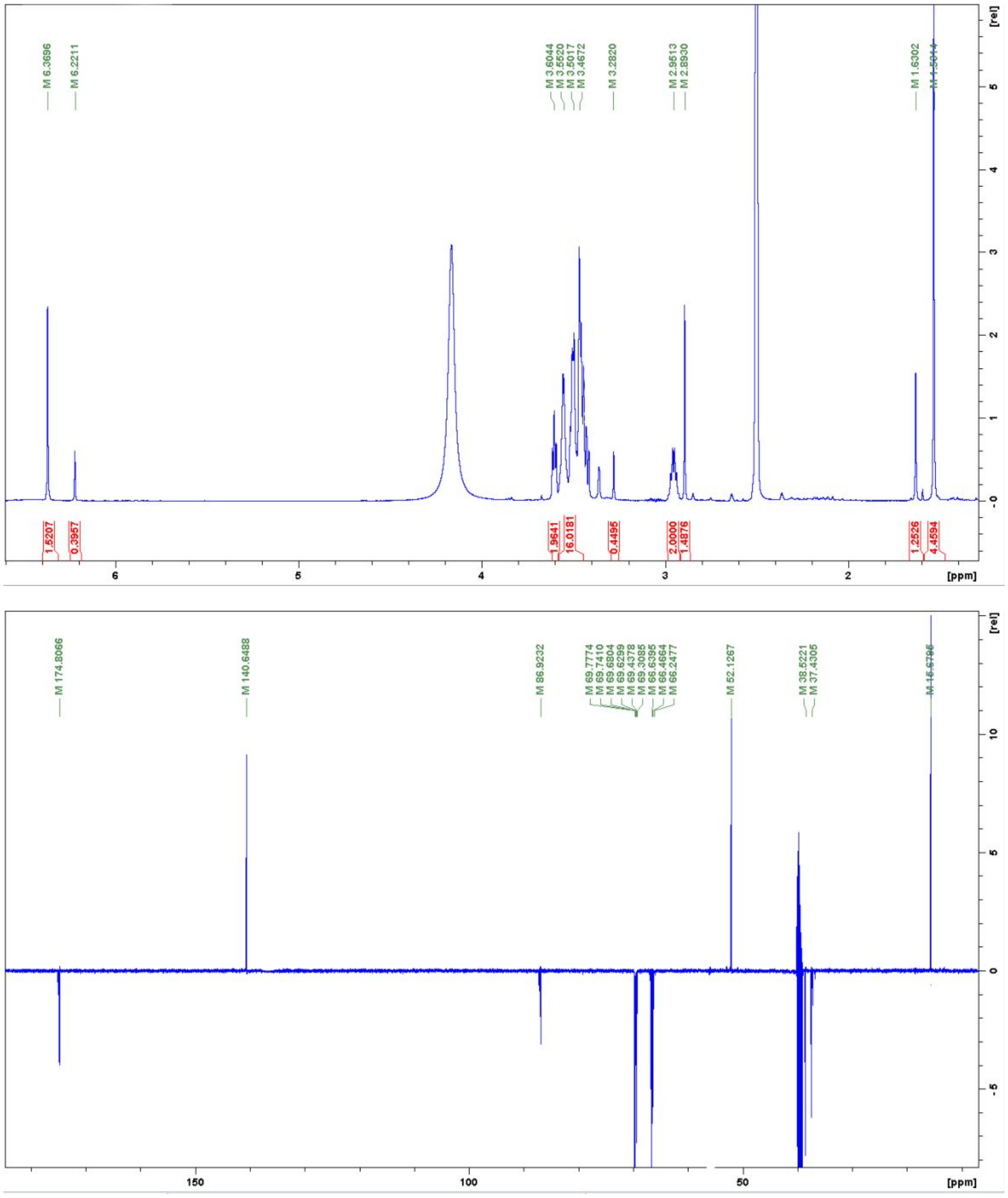

Figure S11. ^{1}H and ^{13}C NMR spectra of **PMal-PEG4- NH$_2$·HCl**, measured in DMSO-d6.

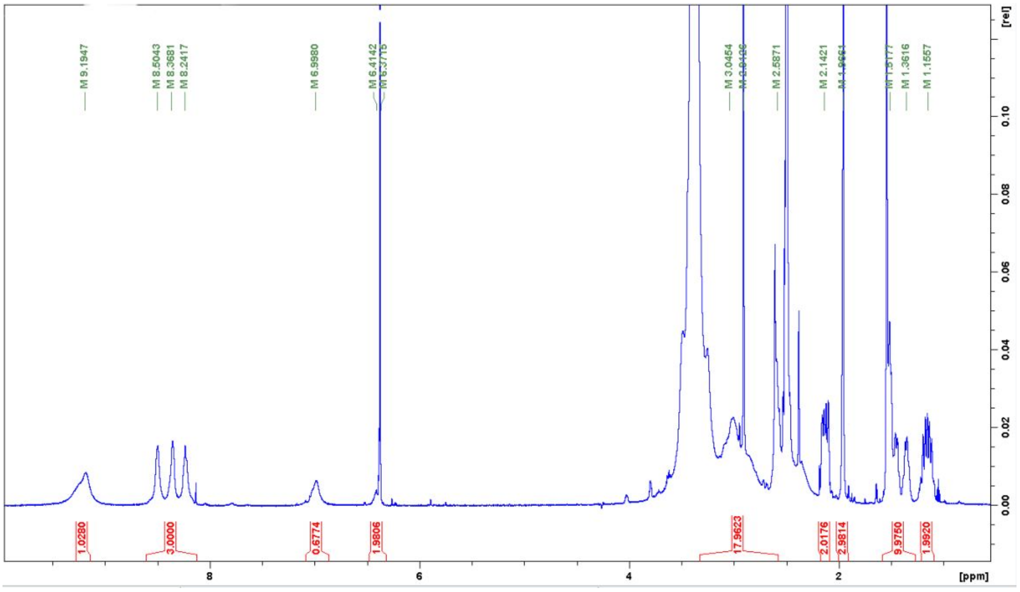

Figure S12. ^{1}H NMR spectrum of **Ox-OAc-PIP-C$_2$-PMal**, measured in DMSO-d6

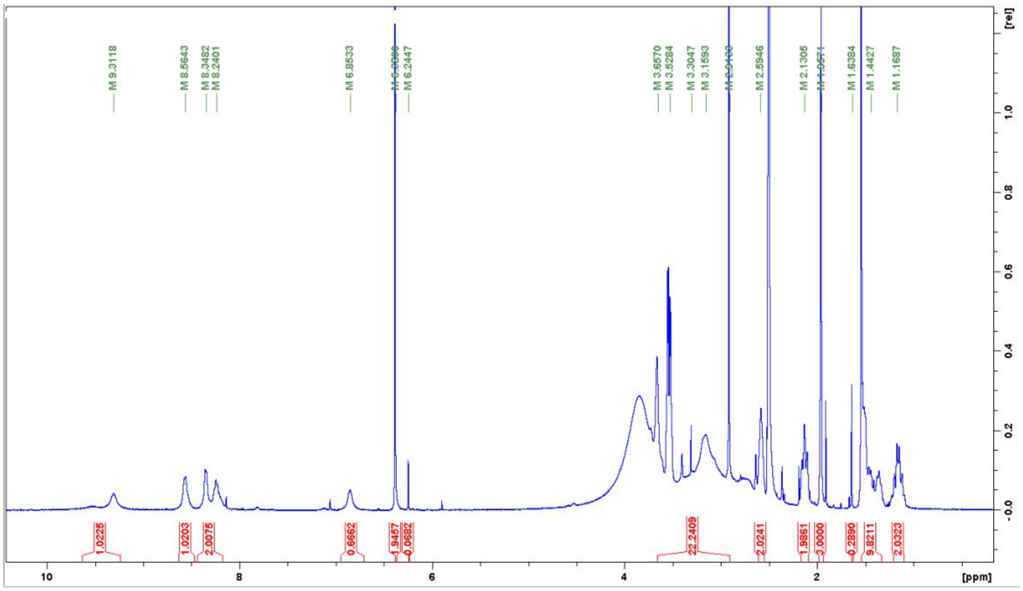

Figure S13. ^{1}H NMR spectrum of **Ox-OAc-PIP-O-PMal**, measured in DMSO-d6

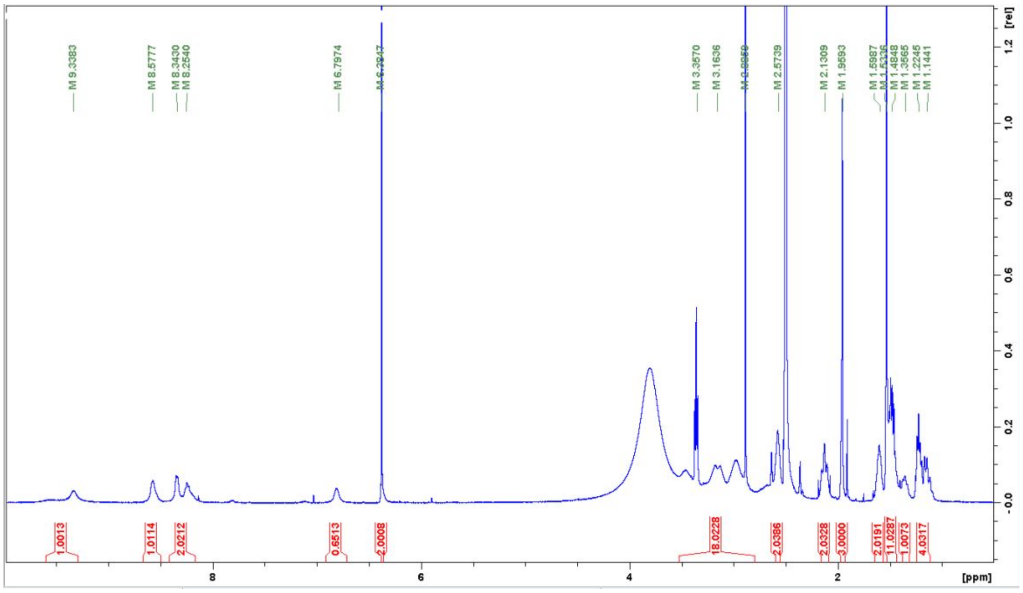

Figure S14. ^{1}H NMR spectrum of **Ox-OAc-PIP-C$_5$-PMal**, measured in DMSO-d6

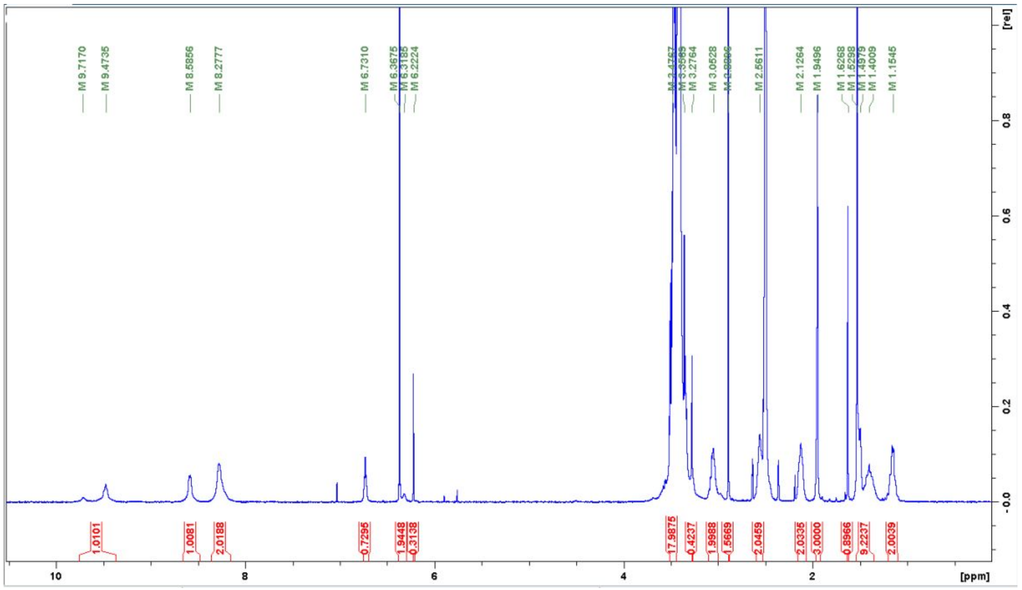

Figure S15. ^{1}H NMR spectrum of **Ox-OAc-PEG4-PMal**, measured in DMSO-d6.

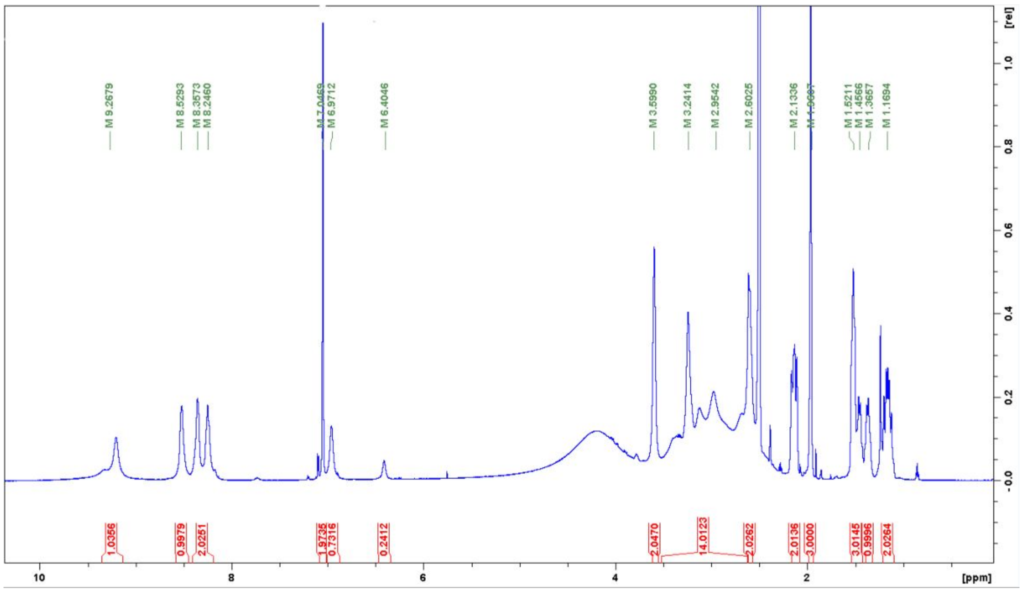

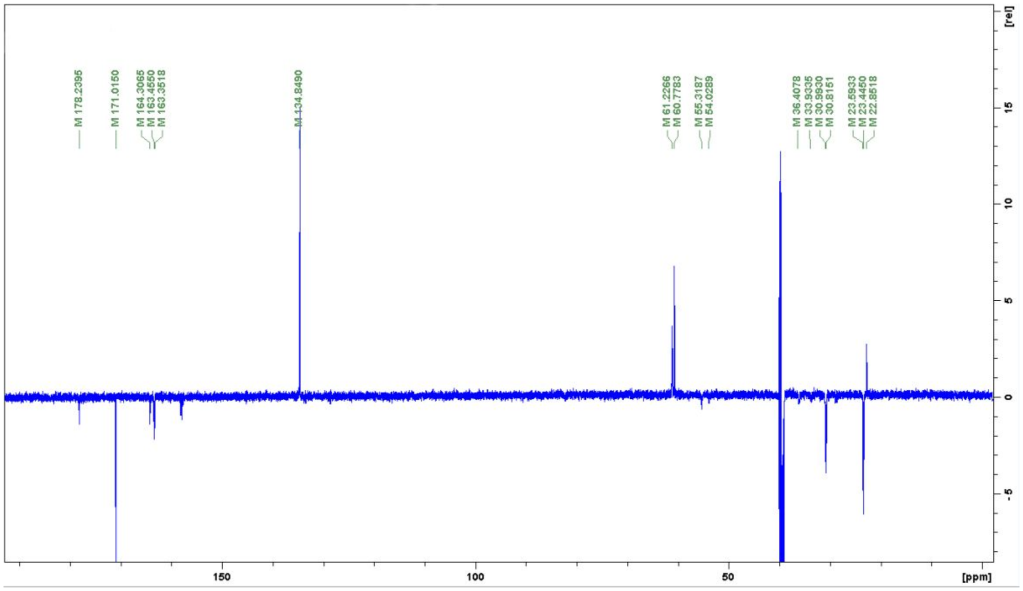

Figure S16. ^{1}H and ^{13}C-NMR spectra of **Ox-OAc-PIP-C_2-Mal**, measured in DMSO-d6.

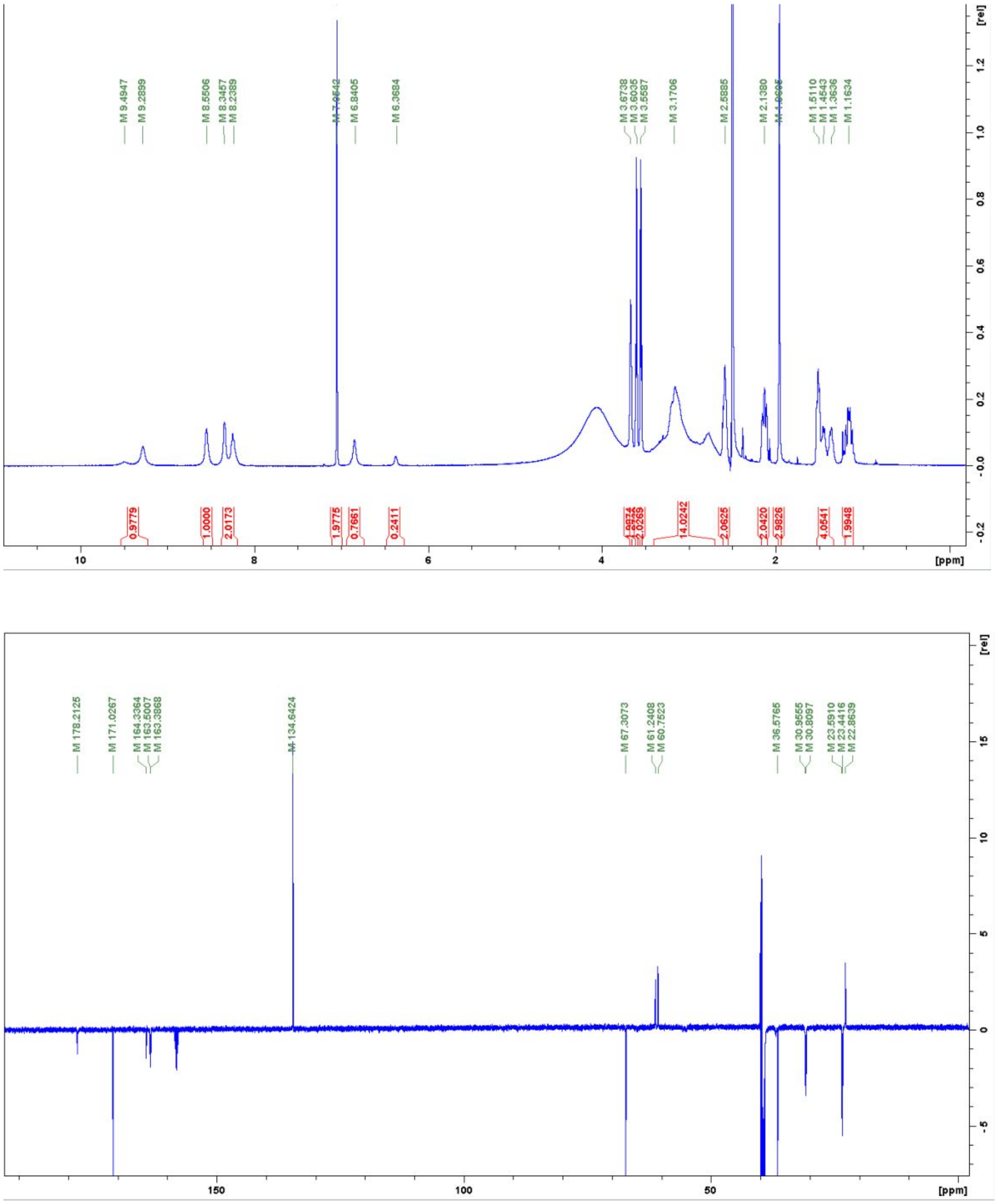

Figure S17. ^{1}H and ^{13}C-NMR spectra of **Ox-OAc-PIP-O-Mal**, measured in DMSO-d6.

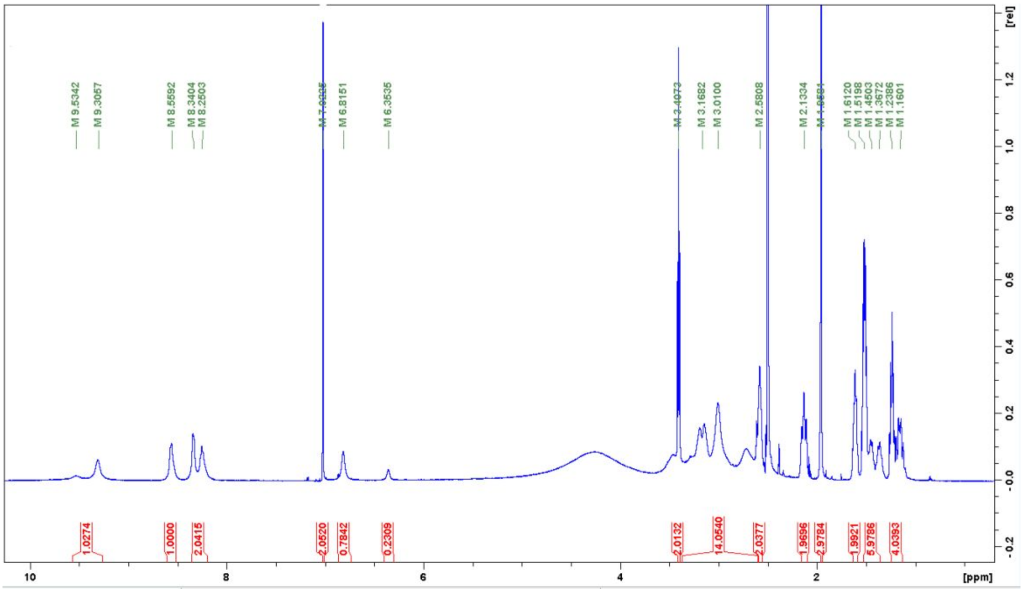

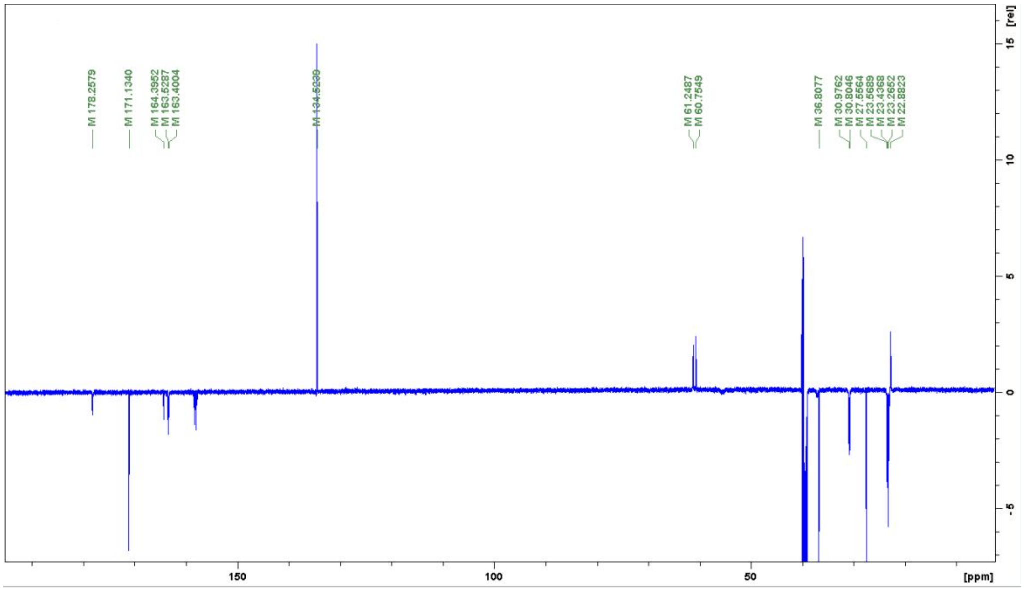

Figure S18. ^{1}H and ^{13}C-NMR spectra of **Ox-OAc-PIP-C$_5$-Mal**, measured in DMSO-d6.

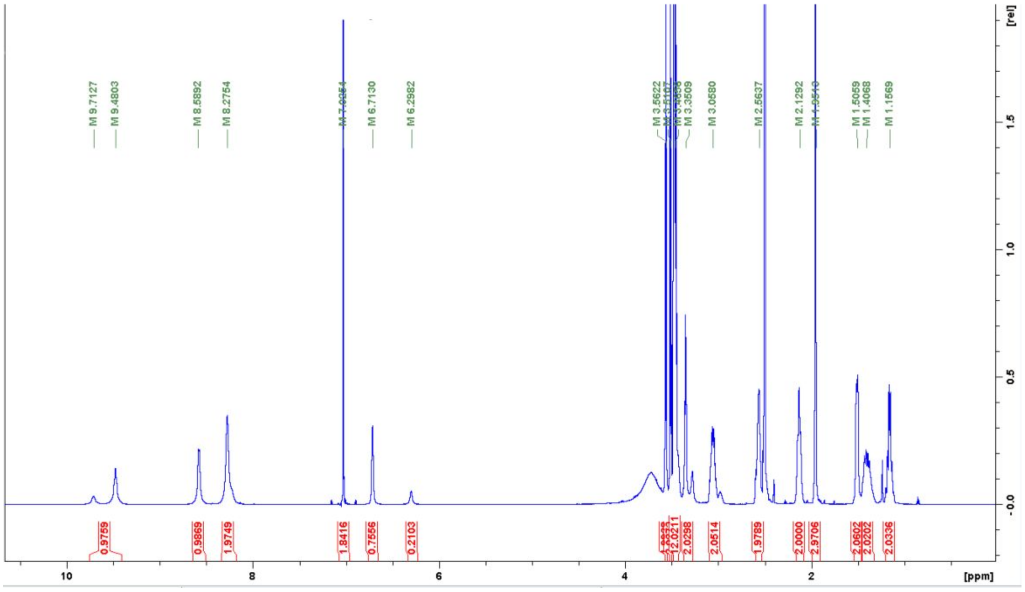

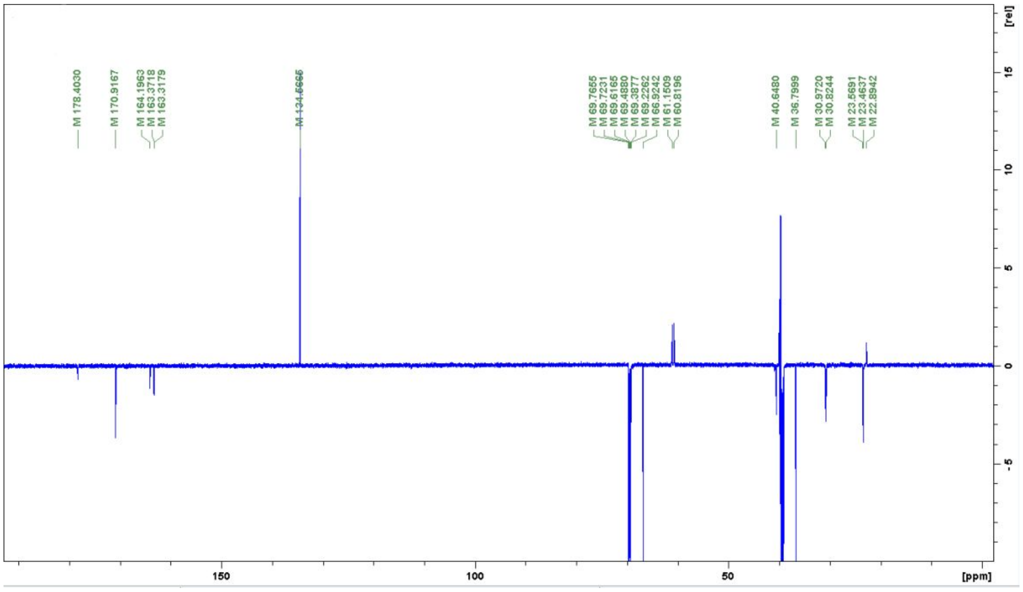

Figure S19. ^{1}H and ^{13}C-NMR spectra of **Ox-OAc-PEG4-Mal**, measured in DMSO-d6.

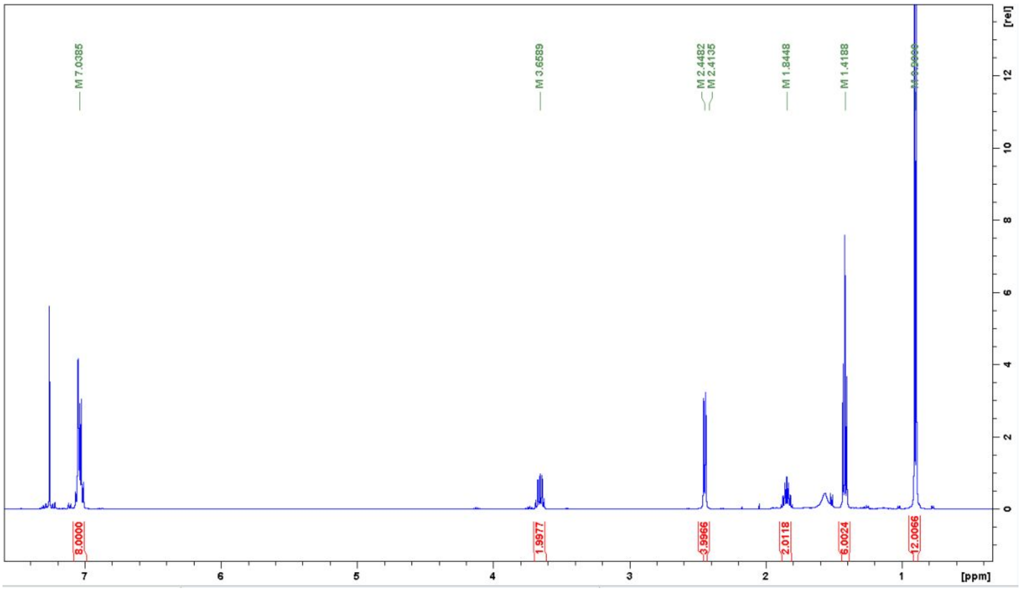

Figure S20. ^{1}H NMR spectrum of **ibuprofen anhydride**, measured in $CDCl_3$.

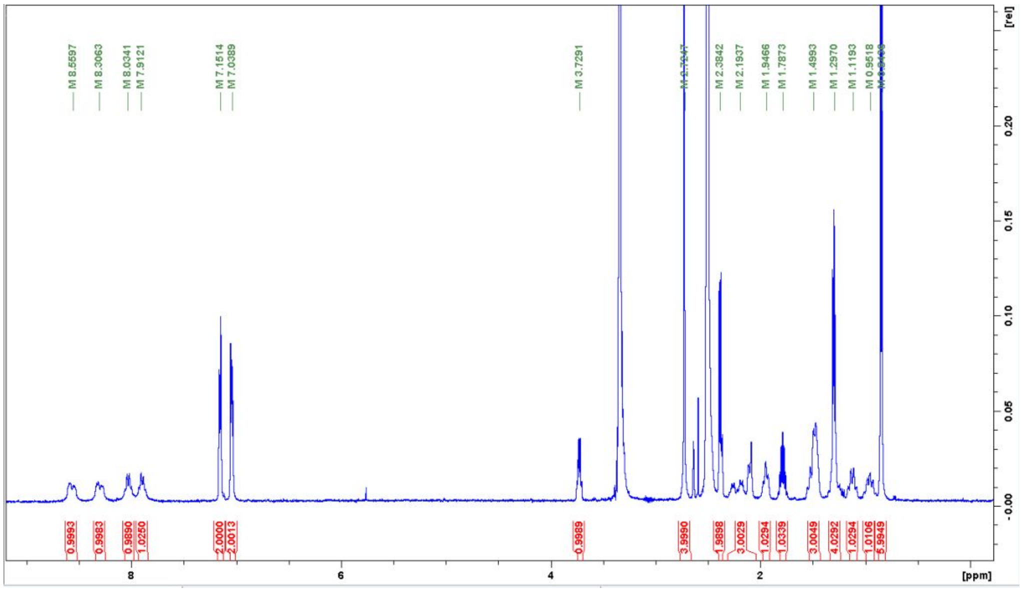

Figure S21. ^{1}H NMR spectrum of **Ox-Ibu-NHS**, measured in DMSO-d6

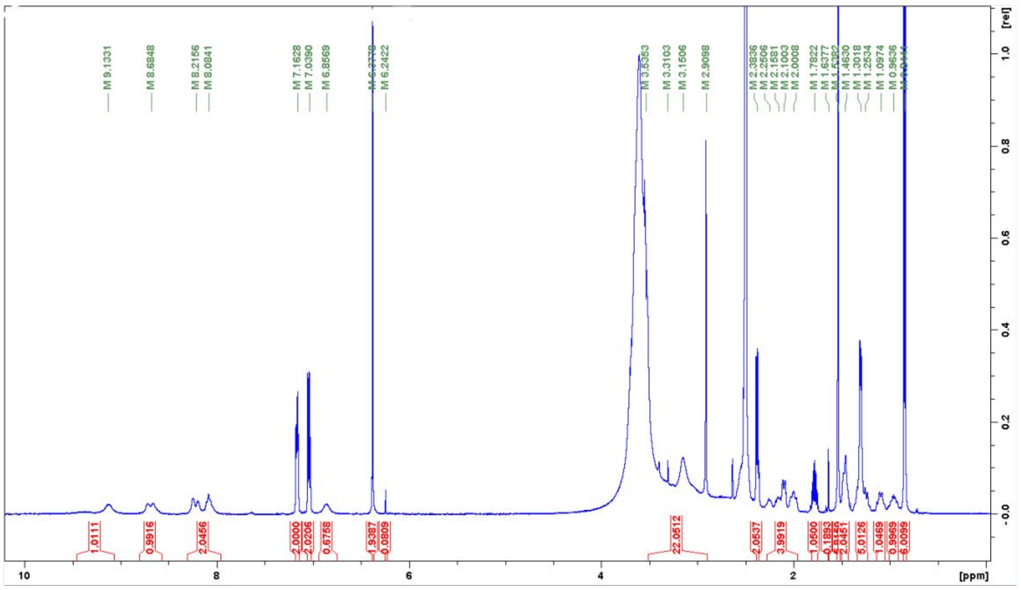

Figure S22. ^{1}H NMR spectrum of **Ox-Ibu-PIP-O-PMal**, measured in DMSO-d6

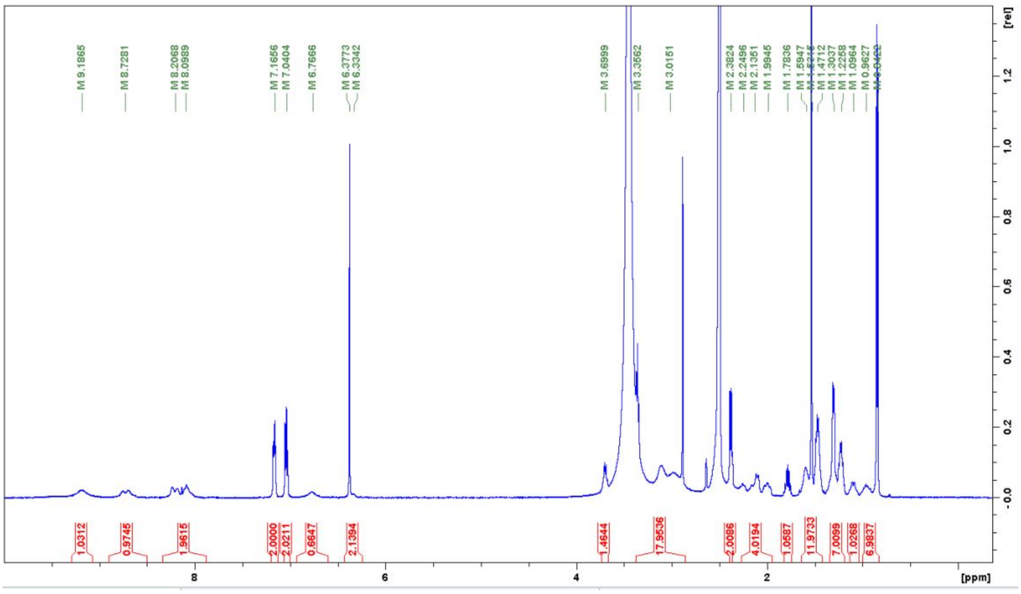

Figure S23. ^{1}H NMR spectrum of **Ox-Ibu-PIP-C$_5$-PMal**, measured in DMSO-d6

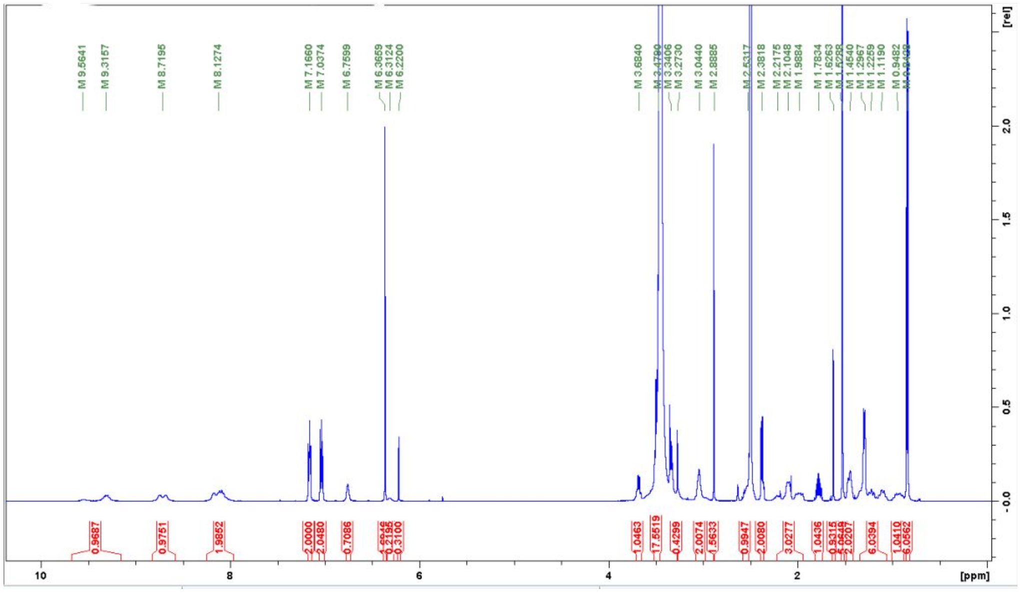

Figure S24. ^{1}H NMR spectrum of **Ox-Ibu-PEG4-PMal**, measured in DMSO-d6.

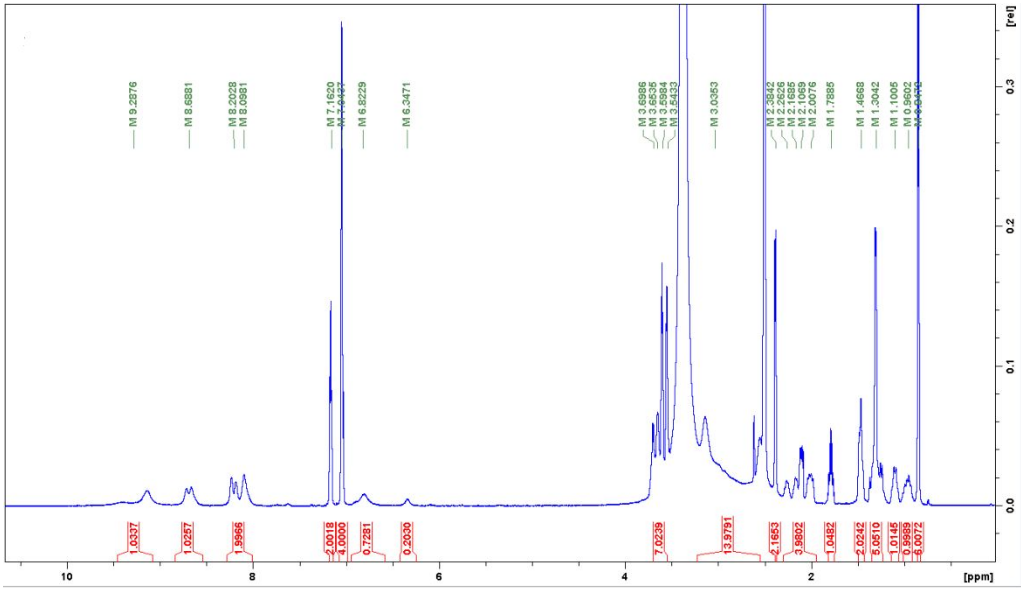

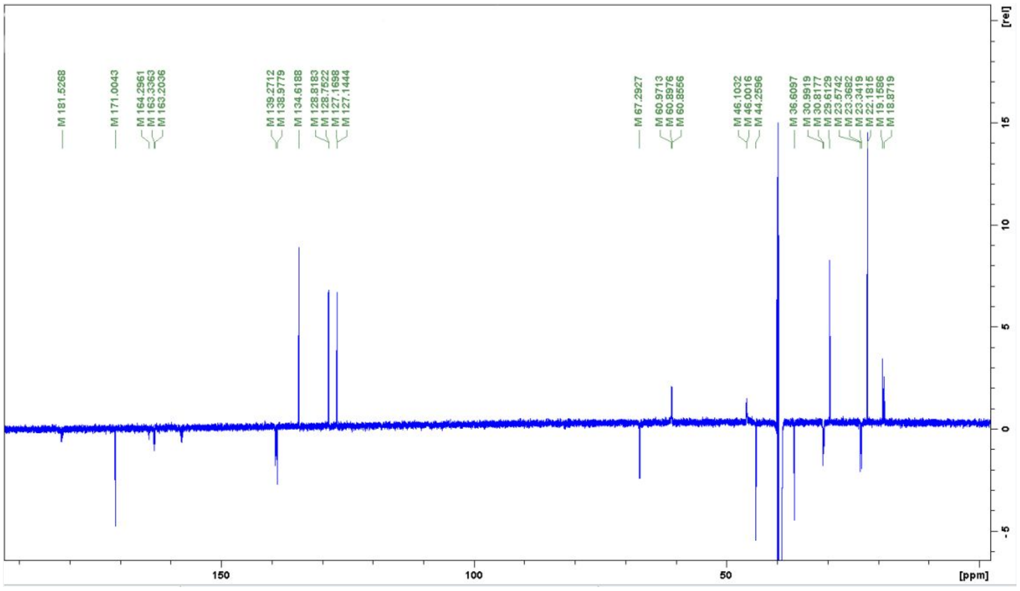

Figure S25. ^{1}H and ^{13}C-NMR spectra of **Ox-Ibu-PIP-O-Mal**, measured in DMSO-d6.

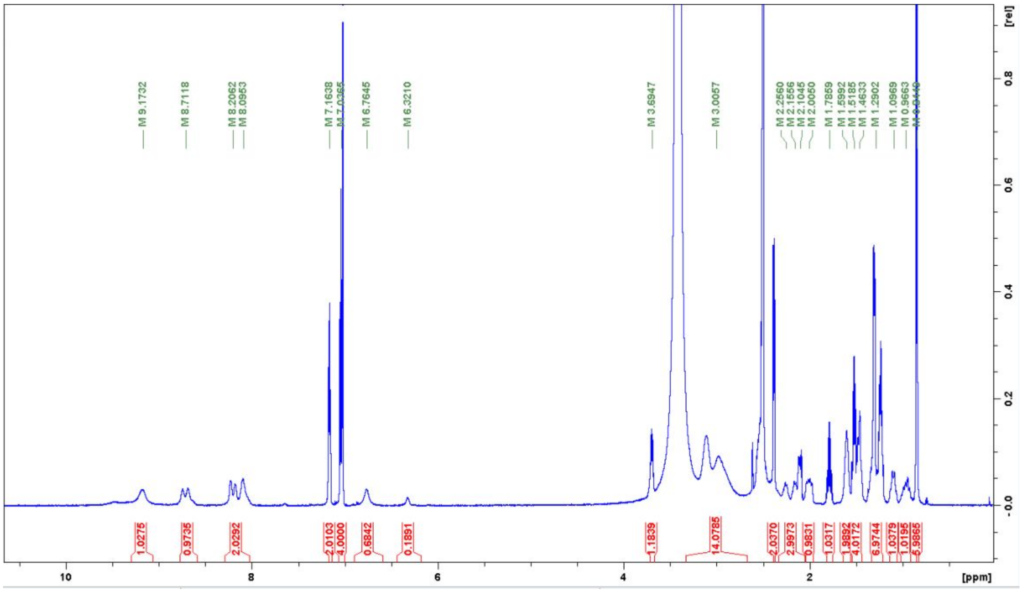

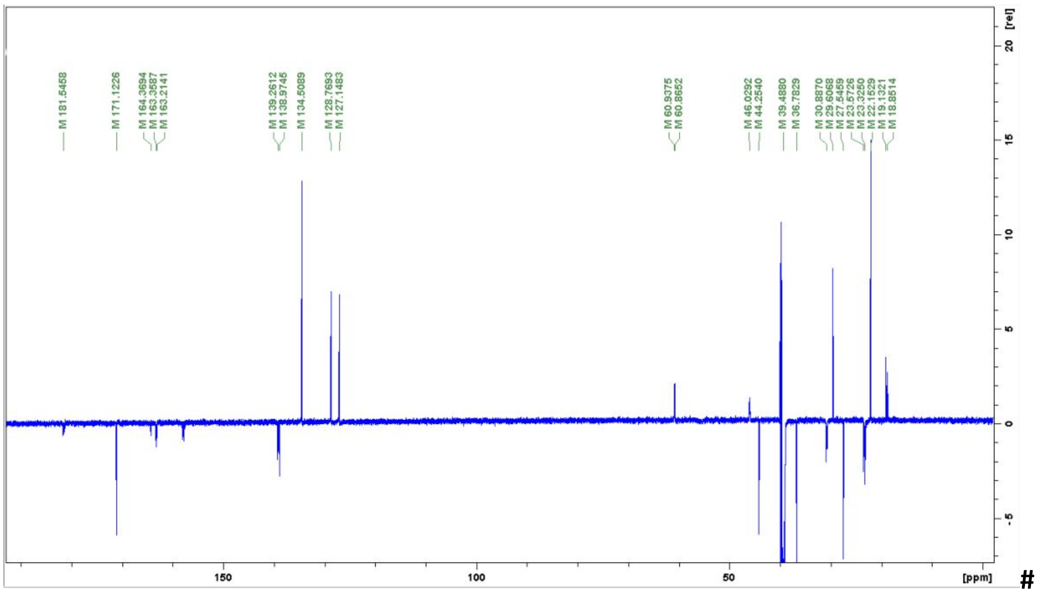

#

Figure S26. ^{1}H and ^{13}C-NMR spectra of **Ox-Ibu-PIP-C_5-Mal**, measured in DMSO-d6.

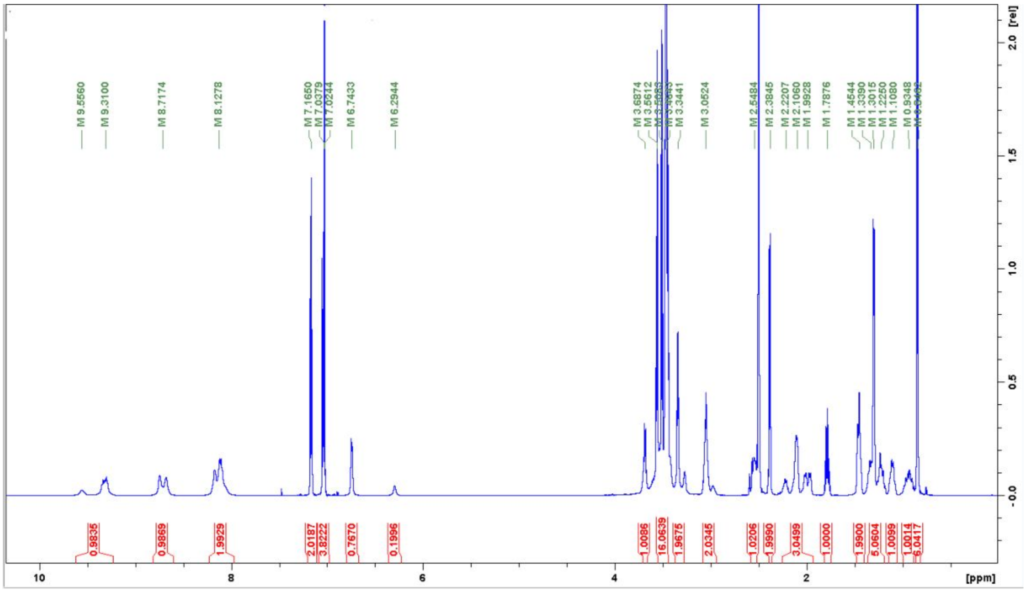

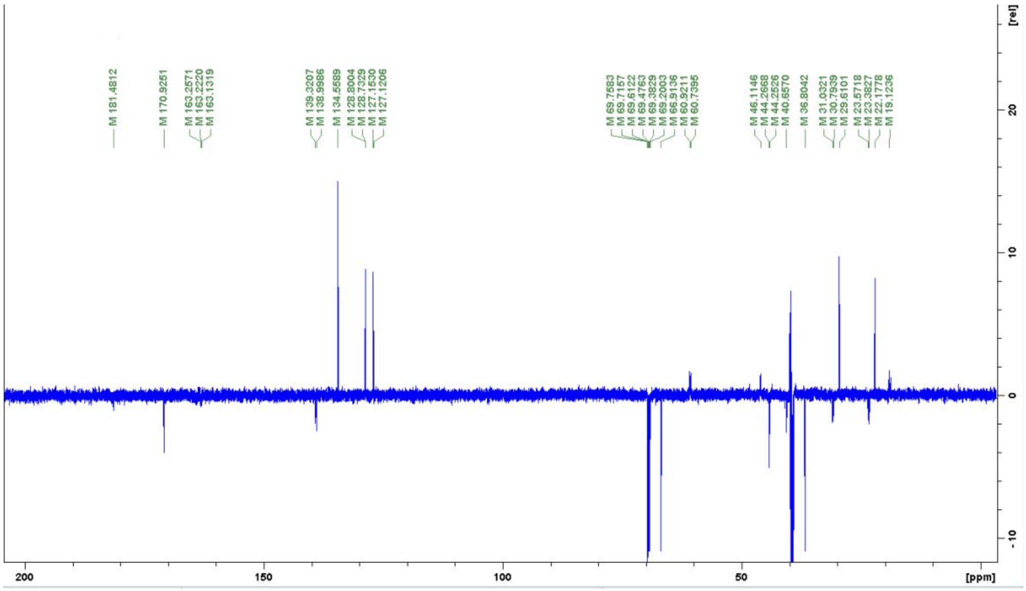

Figure S27. ^{1}H and ^{13}C-NMR spectra of **Ox-Ibu-PEG4-Mal**, measured in DMSO-d6.

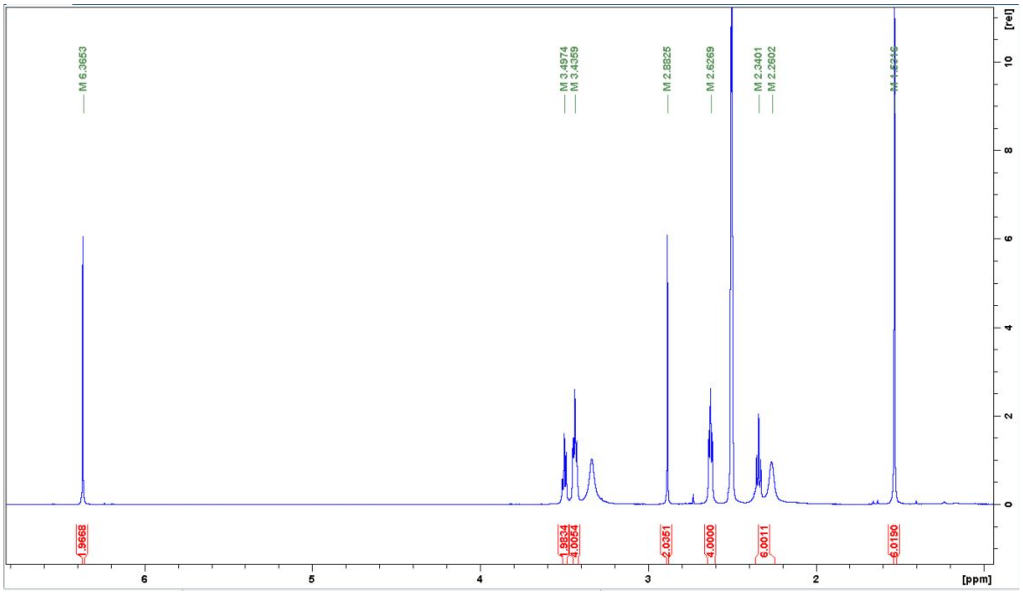

Figure S28. ^{1}H NMR spectrum of **PMal-O-PIP-H**, measured in DMSO-d6

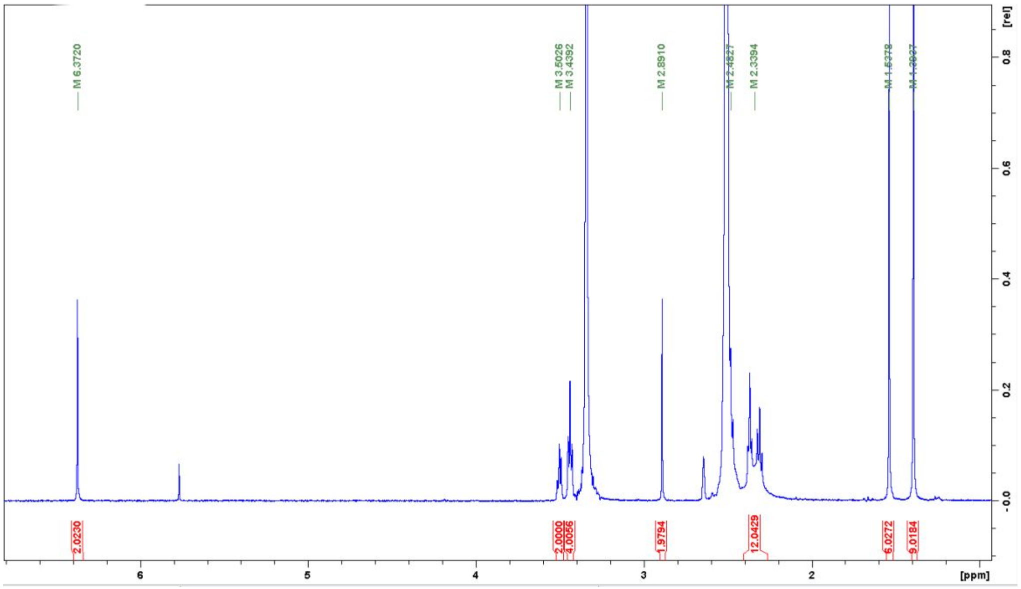

Figure S29. ^{1}H NMR spectrum of **PMal-O-PIP-COOtBu**, measured in DMSO-d6

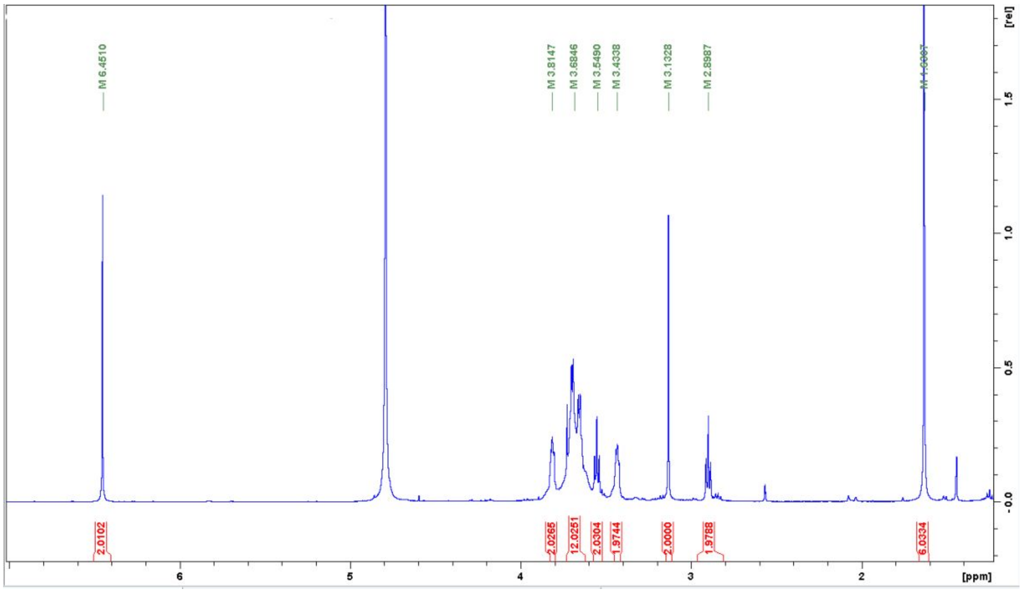

Figure S30. ^{1}H NMR spectrum of **PMal-O-PIP-COOH·2HCl**, measured in D_2O.

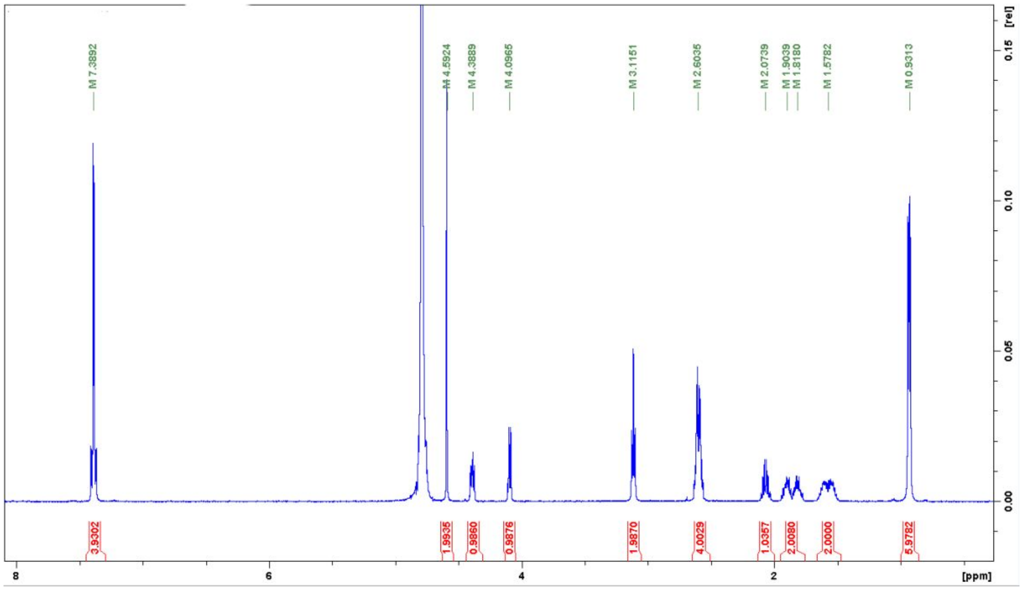

Figure S31. ^{1}H NMR spectrum of **HOOC-ValCitPAB-OH**, measured in D_2O

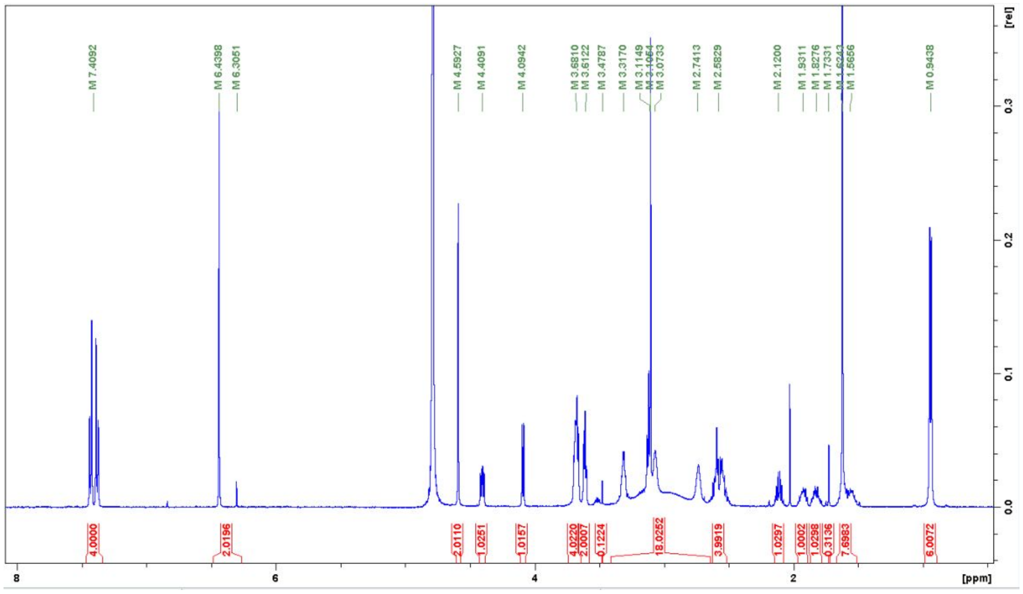

Figure S32. ^{1}H NMR spectrum of **PMal-O-PIP-Succ-ValCitPAB-OH**, measured in D_2O.

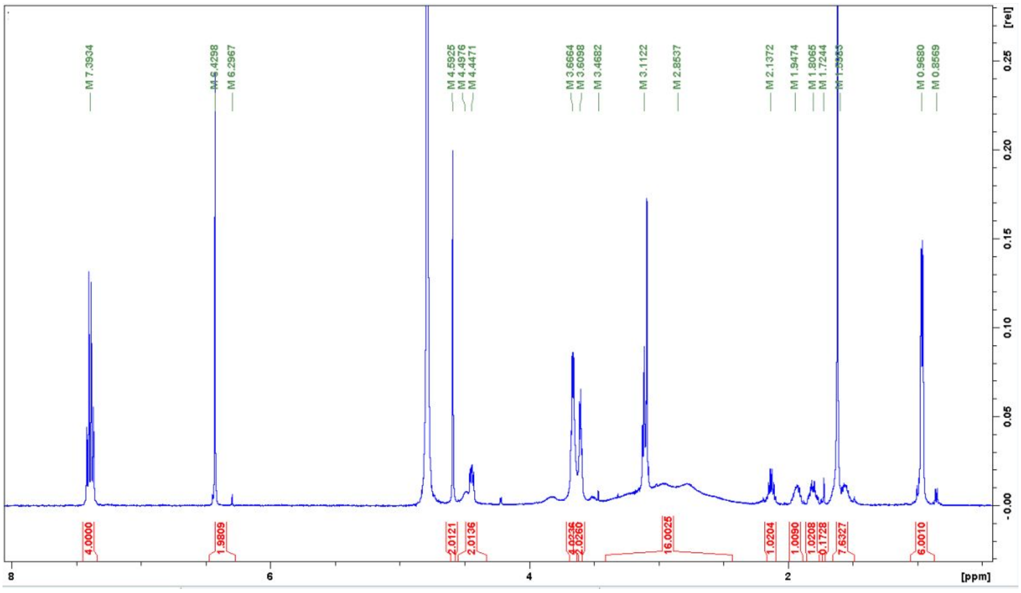

Figure S33. ^{1}H NMR spectrum of **PMal-O-PIP-ThioU-ValCitPAB-OH**, measured in D_2O.

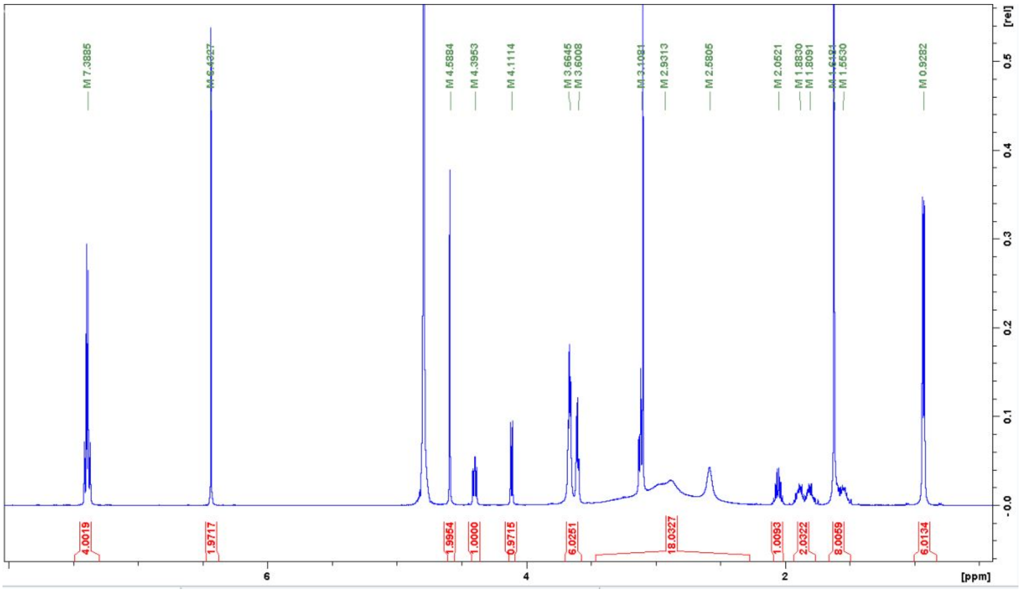

Figure S34. ^{1}H NMR spectrum of P**Mal-O-PIP-ValCitPAB-OH**, measured in D_2O.

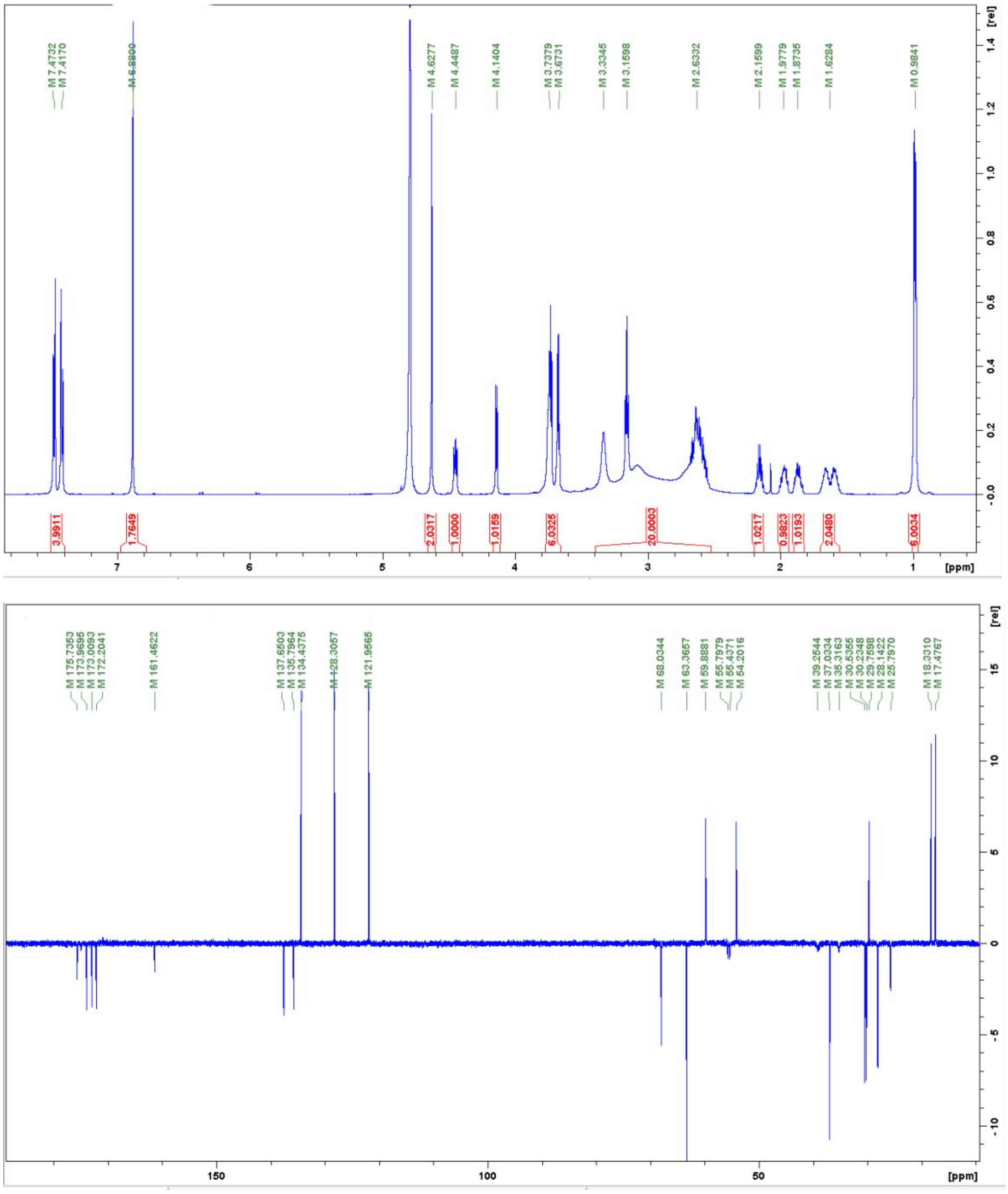

Figure S35. ^{1}H and ^{13}C NMR spectra of **Mal-O-PIP-Succ-ValCitPAB-OH**, measured in D_2O.

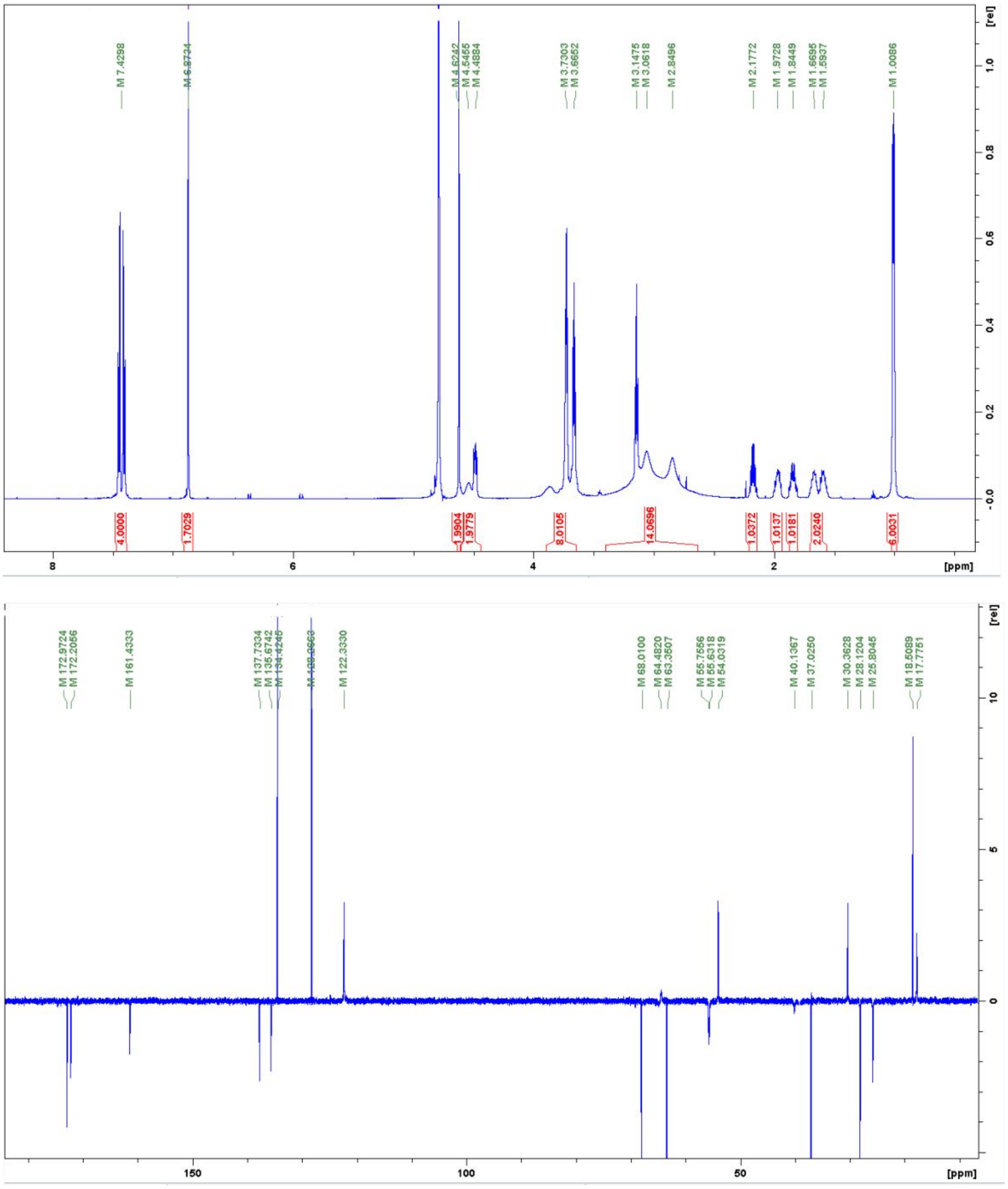

Figure S36. ^{1}H and ^{13}C NMR spectra of **Mal-O-PIP-ThioU-ValCitPAB-OH**, measured in D_2O

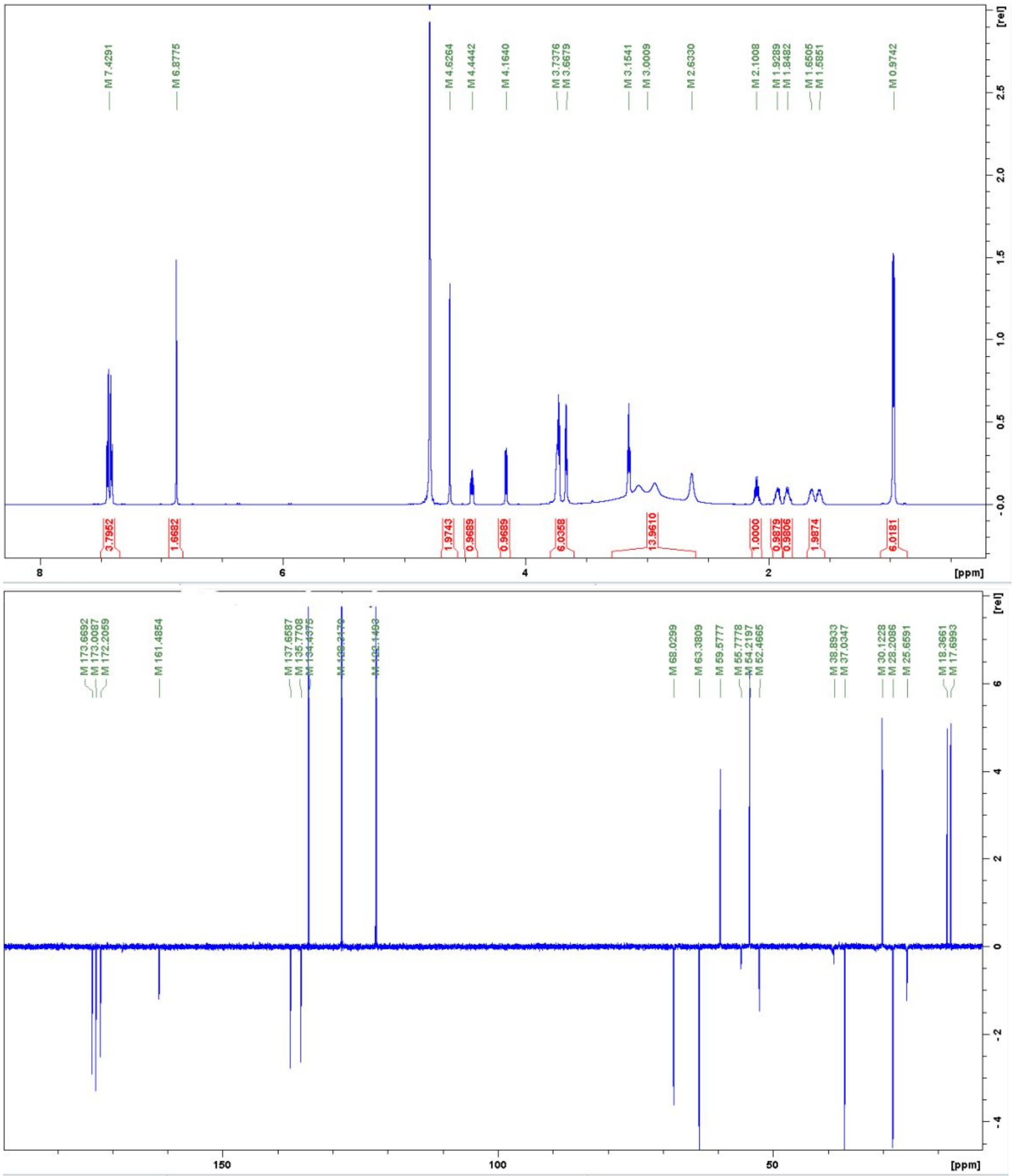

Figure S37. ^{1}H and ^{13}C NMR spectra of **Mal-O-PIP-ValCitPAB-OH**, measured in D_2O.
